# Identifying Health Economic Considerations to Include in the Research Protocol of a Randomized Controlled Trial (the REDUCE-RISK Trial): Systematic Literature Review and Assessment

**DOI:** 10.2196/13888

**Published:** 2021-01-25

**Authors:** Mattias Neyt, Annick Christiaens, Marina Aloi, Lissy de Ridder, Nicholas M Croft, Sibylle Koletzko, Arie Levine, Dan Turner, Richard K Russell, Frank M Ruemmele, Gigi Veereman

**Affiliations:** 1 Medical Evaluation and Technology Assessment (ME-TA) Merendree Belgium; 2 Pediatric Gastroenterology and Nutrition University Hospital Brussels Brussels Belgium; 3 Department of Maternal and Child Health Gastroenterology Unit Sapienza University of Rome Rome Italy; 4 Erasmus MC Sophia Childrens’ Hospital Rotterdam Rotterdam Netherlands; 5 Blizard Institute Barts and the London School of Medicine Queen Mary University of London London United Kingdom; 6 Department of Pediatrics Dr von Hauner Children's Hospital LMU Klinikum University of Munich Munich Germany; 7 Department of Pediatrics, Gastroenterology and Nutrition School of Medicine Collegium Medicum University of Warmia and Mazury Olsztyn Poland; 8 Pediatric Gastroenterology and Nutrition Unit Wolfson Medical Center Holon Israel; 9 Institute of Paediatric Gastroenterology Shaare Zedek Medical Center The Hebrew University of Jerusalem Jerusalem Israel; 10 Department of Paediatric Gastroenterology The Royal Hospital for Sick Children Edinburgh United Kingdom; 11 Hôpital Necker Enfants Malades Assistance Publique - Hôpitaux de Paris Service de Gastroentérologie Pédiatrique Paris France; 12 Faculté de Médecine Université de Paris Paris France; 13 Pediatric Gastroenterology and Nutrition Vrije Universiteit Brussel University Hospital Brussels Brussels Belgium

**Keywords:** Crohn disease, cost-benefit analysis, adalimumab, clinical trial, protocol, technology assessment, biomedical

## Abstract

**Background:**

The REDUCE-RISK trial was set up to compare the effectiveness of weekly subcutaneously administered methotrexate with daily oral azathioprine or 6-mercaptopurine in low-risk Crohn disease (CD) or subcutaneously administered adalimumab (ADA) in high-risk CD in a pediatric population (age 6-17 years).

**Objective:**

The aim of this study is to perform a systematic review to provide input into the research protocol to gather the necessary information to improve the performance of an evidence-based economic evaluation when the trial is finished.

**Methods:**

The Centre for Reviews and Dissemination (CRD) Health Technology Assessment (HTA) database, websites of HTA institutes, CRD’s National Health Service Economic Evaluation Database, MEDLINE (OVID), and Embase databases were consulted to retrieve (reviews of) relevant economic evaluations. Studies were eligible if they included a pediatric or adult population with inflammatory bowel diseases (CD and ulcerative colitis [UC]) treated with ADA (Humira). There were no restrictions on the comparator. Only economic evaluations expressing outcomes in life years gained or quality-adjusted life years gained were selected.

**Results:**

A total of 12 primary studies were identified. None of these studies included a pediatric population because of a lack of supporting trials. The economic evaluations identified in our systematic review indicate that ADA is an appropriate intervention for inclusion in such a trial. From a health economic point of view, it is important to make an incremental analysis comparing such an intervention with standard care and not immediately versus another (expensive) biological treatment. Information on the impact of children’s school attendance and parents’ productivity is currently lacking in economic evaluations, and none of the underlying trials measured quality of life (QoL) using a generic utility instrument.

**Conclusions:**

The review of the economic literature on ADA for the treatment of patients with CD supports the performance of a trial with biologicals in pediatric patients, including making a distinction according to disease severity. Conducting an economic literature review enabled us to decide which variables should be added to the research protocol from an economic point of view. Measurements for children’s and parents’ QoL (EuroQol 5-Dimension questionnaires), children’s school attendance, and parents’ productivity (WPAI-CD-CG questionnaire) were added to the research protocol. This will provide support for the calculation of the cost-effectiveness of the interventions evaluated in the REDUCE-RISK trial.

**Trial Registration:**

ClinicalTrials.gov NCT02852694; https://clinicaltrials.gov/ct2/show/NCT02852694

## Introduction

### The REDUCE-RISK Trial

Immunomodulators such as thiopurines (azathioprine [AZA] or 6-mercaptopurine [6-MP]), methotrexate (MTX), and biologicals such as adalimumab (ADA) are well established for the maintenance of remission in pediatric Crohn disease (CD). However, it remains unclear which maintenance medication should be used first line in specific patient groups. The REDUCE-RISK trial (*Risk-stratified randomized controlled trial in paediatric Crohn’s Disease: Methotrexate versus azathioprine or adalimumab for maintaining remission in patients at low or at high risk for aggressive disease course, respectively – a treatment strategy*) aims to compare the efficacy of maintenance therapies in newly diagnosed CD based on stratification into high- and low-risk groups for severe CD evolution: (1) MTX versus AZA/6-MP in low-risk patients and (2) MTX versus ADA in high-risk patients. Patients are eligible if aged 6 to 17 years with new-onset (<6 months) treatment-naïve active and/or perianal fistulizing CD and receiving steroids or exclusive enteral nutrition (EEN) for induction of remission. They are stratified into low- and high-risk groups based on phenotype and disease response to induction therapy. Individual informed consent is obtained before participation in the study. Patients are followed up for 12 months post randomization at prespecified intervals. The primary endpoint is sustained steroid or EEN-free remission at 12 months [1].

The REDUCE-RISK trial, an international multicenter open-label prospective randomized controlled trial (RCT), has received funding from the European Union’s Horizon 2020 research and innovation programme under grant agreement number 668023. This trial has been reviewed and approved by the National Ethics Services of participating countries and is prospectively registered (ClinicalTrials.gov Identifier: NCT02852694; date of registration: June 9, 2016; EudraCT Number: 2016-000522-18).

### Background on Health Technology Assessment and Economic Evaluations

When setting up the protocol for REDUCE-RISK, the research team prepared to allow the performance of a full health technology assessment (HTA). The European Network for HTA (EUnetHTA) describes HTA as “a multidisciplinary process that summarizes information about the medical, social, economic and ethical issues related to the use of a health technology in a systematic, transparent, unbiased, robust manner. Its aim is to inform the formulation of safe, effective, health policies that are patient focused and seek to achieve best value. Despite its policy goals, HTA must always be firmly rooted in research and the scientific method*.*” In HTA, an economic evaluation is performed to determine whether an intervention offers value for money in comparison with other alternatives. This economic consideration might provide support to policy makers when making decisions while trying to make efficient use of limited resources.

An economic evaluation is “the comparative analysis of alternative courses of action in terms of both their costs and consequences” [2]. In an economic evaluation, the incremental cost-effectiveness ratio (ICER) is calculated using the following general formula:

ICER=IC/IE=(C_Int_−C_Comp_)/(E_Int_−E_Comp_)

with C being the costs, Comp the comparator, E the effects, IC the incremental cost, IE the incremental effect, and Int the intervention.

This formula shows that the focus should be on the incremental elements, that is, those that differ between the compared alternatives. Economic evaluations can be performed from different perspectives. As mentioned in the EUnetHTA guideline on methods for health economic evaluations [3], “economic evaluations should at minimum be conducted from a health care perspective. However, several countries require a societal perspective.” To make the results of the international REDUCE-RISK trial useful for researchers and policy makers in different countries, incremental elements for both the health care payer and societal perspective will be taken into account.

### A Review of the Literature to Provide Input to the Research Protocol

In preparation of a future economic evaluation, we determine the most important incremental elements. The International Society for Pharmacoeconomics and Outcomes Research (ISPOR) guidelines state that “assessing relative value is rarely the primary purpose of an experimental study. Nevertheless, when the decision is made to conduct an economic evaluation alongside a clinical trial, it is important that the economic investigator contributes to the design of the study to ensure that the trial will provide the data necessary for a high-quality economic evaluation” [4]. Our research question is *which additional elements should we include in the research protocol of the REDUCE-RISK trial to provide support to a high-quality economic evaluation?* Therefore, a systematic search for economic literature about the cost-effectiveness of ADA (Humira) for the treatment of inflammatory bowel disease (IBD) was performed.

The purpose of this systematic review is to obtain more useful insights and knowledge from previous economic studies [5]. These previous economic evaluations guide us in finding the key variables that enable us to provide well-directed input for the research protocol. In this paper, the review of the economic literature is transparently presented. No official review protocol was established for the systematic review. The findings are used to provide input for the research protocol from a health economic point of view (eg, to decide which questionnaires should be added to the research protocol). On the other hand, we also want to avoid overloading the research protocol and only focus on the incremental elements that influence an intervention’s cost-effectiveness. The results of this systematic review help us to focus on gathering the right information in the REDUCE-RISK trial, which will support researchers at the end of the trial to make a high-quality economic evaluation.

## Methods

A systematic review of the literature was conducted using predefined selection criteria that included considerations of population, intervention, comparator, and design. As the goal of this study is to provide input for the research protocol, the applied selection criteria were not too restrictive. Studies were included if (1) the population included children or adults with IBD (CD and ulcerative colitis [UC]); (2) ADA was one of the included interventions; and (3) the design reflected a full economic evaluation, that is, studies comparing at least two alternative treatments in terms of costs and outcomes and expressing outcomes in life years gained or quality-adjusted life years (QALYs) gained. No restrictions were applied to the comparator. Studies were excluded if they only considered other treatments than ADA at the moment of randomization. Studies that only included switching to ADA in case of no response to the interventions under consideration (ie, not including ADA at the moment of randomization) were not selected. Cost analysis or cost-of-illness studies did not fulfill the aforementioned definition of an economic evaluation and were excluded. As summarized in an EUnetHTA guideline providing an overview of national guidelines for 25 countries, “all countries except four specify that the preferred outcome measure is QALYs or both QALYs and life years. Of the four countries with guidelines that do not announce QALYs as a preferred method, at least three accept QALYs in special circumstances or in complementary analyses” [3]. In this study, we focus on these preferred outcomes. Studies expressing results in disease-specific outcomes (eg, cost per remission [6,7], cost per responder [8], or cost per mucosal healing [9]) are thus excluded. *Before-after* analyses [10] comparing the costs before and after the start of treatment with ADA were also excluded, as they also do not fulfill the definition of an economic evaluation (ie, lack of a comparative intervention). Abstracts were excluded because of a lack of sufficient details to allow for a proper evaluation. No time restriction was imposed. English, French, German, and Dutch papers were eligible.

Various databases were consulted. In February 2016, before the final protocol was set up, reviews on this topic were searched by consulting the Centre for Reviews and Dissemination (CRD) HTA database and HTA institute websites listed on the International Network of Agencies for Health Technology Assessment website. The websites of ex- or nonmember HTA institutes such as the National Institute for Health and Care Excellence (NICE) were also consulted. In September 2016, CRD’s National Health Service Economic Evaluation Database, MEDLINE (OVID), and Embase databases were searched to retrieve both full economic evaluations and reviews of full economic evaluations of ADA for IBD treatment. To ensure reproducibility, further details of the search strategy are provided in Multimedia Appendix 1.

The selection of relevant papers was performed in a 2-step procedure: an initial assessment of the title, abstract, and keywords, followed by a full-text assessment of the selected references. When no abstract was available and the citation was unclear or ambiguous, consideration of the citation was made directly on the basis of a full-text assessment. Reference lists of the selected studies were checked for additional relevant citations. The procedure was performed by a health economist (MN), and in case of doubt for medical reasons, a medical specialist (GV) provided support.

The primary full economic evaluations were summarized in an in-house data extraction sheet listing all variables (eg, population, intervention, comparators, and quality of life [QoL] input) and summary measures (eg, ICERs and results of sensitivity analyses) for which data were sought (Multimedia Appendix 1). The information gathered in these sheets reflects the elements that are usually reported in an economic evaluation (eg, according to the Consolidated Health Economic Evaluation Reporting Standards [CHEERS] guidelines [11,12]). This information was used to set up summary tables that form the basis for a further critical assessment. On the basis of the results of this assessment, we judged whether from an HTA and economic perspective, elements in the research protocol of the REDUCE-RISK trial could be added.

## Results

### Article Selection

**Figure 1:**
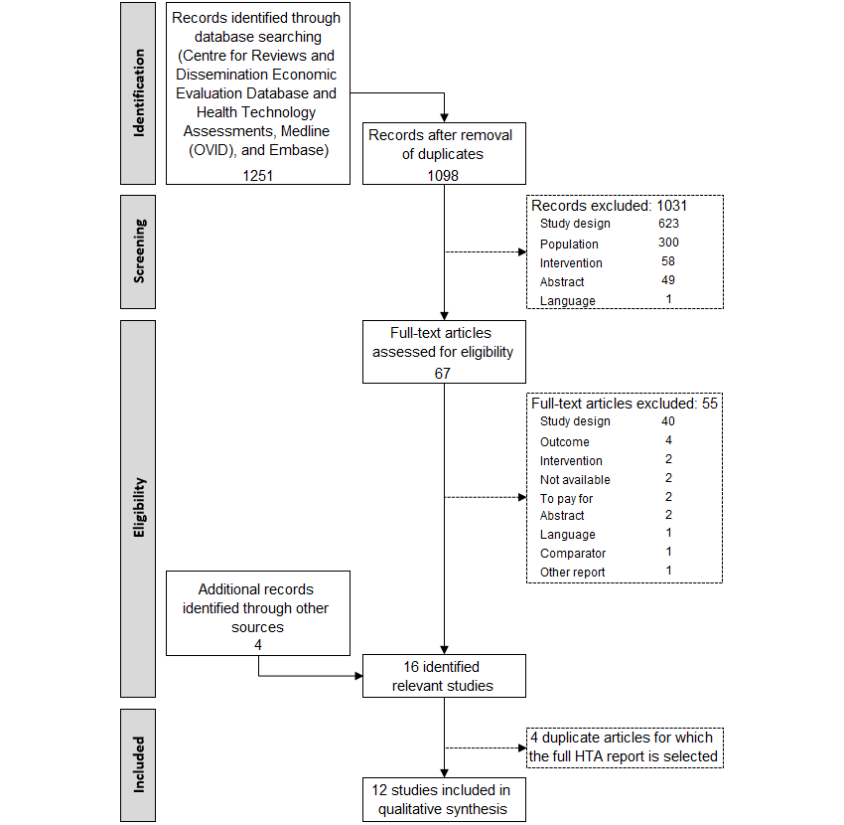
Selection of relevant articles.

Figure 1 presents the flowchart of the selection process. A total of 12 papers were identified in the electronic databases. Four additional references were identified by searching websites of HTA institutes. Information from 3 journal papers [13-15] and 1 report [16] were already published in an HTA report. To avoid overlap, only the 12 primary studies [17-28] will be further discussed. The list of the 16 identified economic evaluations and information on duplicates is provided in Multimedia Appendix 2.

### General Information

Half of the studies were performed for the United Kingdom (n=6; Table 1). Two studies conducted an analysis for Canada, another 3 for the United States, and 1 for Poland. All but one of the studies explicitly declared the presence or absence of conflicts of interest. All studies were cost-utility analyses. Most short-term models (1 year) applied a decision tree, whereas long-term analyses (5, 10, or 30 years or lifetime) are Markov models or a combination of an initial decision tree and a Markov component. For the long-term models, applied discount rates are in agreement with national recommendations in all but one of the analyses. In this study, the manufacturer assumed an annual discount rate of 3% for both health and cost outcomes, although the Canadian Agency for Drugs and Technologies in Health (CADTH) guidelines recommend a 5% discount rate [19]. However, a 5% discount was applied in the sensitivity analysis. For further details, we refer to section 2.3.1 of the Multimedia Appendix 1.

**Table 1:** General information on the identified economic evaluations.

Study		Country	CoI^a^	Analytic technique	Design	Time horizon	Discount rate^b^ (%)
**Archer et al [17]**		United Kingdom	No	CUA^c^	Markov	Lifetime	3.5
	AbbVie submission^d^	N/A^e^	Yes	CUA	Markov	10 years	3.5
	MSD^f^ submission^d^	N/A	Yes	CUA	Decision tree+Markov	10 years	3.5
Assasi et al [18]		Canada	No	CUA	Markov	5 years	5
Bodger et al [23]		United Kingdom	No	CUA	Markov	Lifetime (60 years)	3.5
CADTH [19]		Canada	Yes or no^g^	CUA	Markov	10 years	3
**Dretzke et al [20]**		United Kingdom	No	CUA	Markov	1 year	—^h^
	Abbott submission^i^	N/A	Yes	CUA	Markov	1 year (56 weeks)	—
Essat et al [21]		United Kingdom	Yes or no^j^	CUA	Decision tree+Markov	10 years	3.5
Kaplan et al [24]		United States	Yes	CUA	Decision tree	1 year	—
Loftus et al [25]		United Kingdom	Yes	CUA	Regression model	1 year	3.5
Rafia et al [22]		United Kingdom	Yes or no^j^	CUA	Decision tree+Markov	10 years	3.5
Stawowczyk et al [26]		Poland	No	CUA	Markov	30 years	Costs: 5; Effects: 3.5
Tang et al [27]		United States	Not declared	CUA	Decision tree	1 year (54 weeks)	—
Yu et al [28]		United States	Yes	CUA	Decision tree	1 year (56 weeks)	—

^a^CoI: conflict of interest.

^b^Discount rate for both costs and effects, unless otherwise mentioned.

^c^CUA: cost-utility analysis.

^d^The AbbVie and MSD submissions are part of the report published by Archer et al [17].

^e^N/A: not applicable. This submission is part of the UK report in which it was published.

^f^MSD: Merck Sharp & Dohme.

^g^Submission by the manufacturer reviewed by the CADTH team (Common Drug Review Analyses).

^h^No discount rate is applied because of the short time horizon of 1 year.

^i^Abbott submission is part of the report published by Dretzke et al [20].

^j^The manufacturer submitted a model-based health economic analysis as part of their submission, which was then evaluated by a team of researchers from the School of Health and Related Research.

### Population and Compared Interventions

None of the studies included a pediatric population. The primary economic evaluations investigated treatment strategies for adult patients (average age 35-40 years and average weight 69-77 kg) with moderate-to-severe UC or CD. In two studies, a secondary analysis is considered for the pediatric population [17,20]. The authors consider this as an exploratory analysis, as the efficacy data are drawn from trials undertaken within an adult UC population [17].

Most studies explicitly mention that patients failed (intolerance, inadequate response, or loss of response) to respond to standard therapy before ADA was considered. In all but 3 studies [24,27,28] and the MSD submission [17], conventional nonbiological therapy is considered as a comparator. This usually exists as a mix of 5-aminosalicylates (5-ASAs), corticosteroids, and immunosuppressants. In 2 studies, only biologicals are included [24,28]. The study by Kaplan et al [24] considered whether dose escalation of infliximab (IFX; to 10 mg/kg every 8 weeks) is a cost-effective strategy compared with ADA initiation after loss of response to 5 mg/kg of IFX. In addition, Yu et al [28] compared IFX and ADA. This study was also part of the Abbott submission, which contained 2 models: one comparing the cost-effectiveness of ADA as a maintenance therapy against standard care (SC) and the other comparing the cost-effectiveness of ADA and IFX as maintenance therapies [20]. The report of Dretzke et al [20], which made a critical assessment of Abbott’s submission, concentrates on the model including SC as a treatment option (refer to the *Discussion* section).

In 2 studies [17,21] and the MSD submission [17], surgery (colectomy) is considered as an initial treatment option. In these studies, surgery is included as an alternative treatment strategy and a downstream component of the pathway for patients in the other treatment strategies. In other models, such as the models discussed in the CADTH report [19] and from the AbbVie submission [17], surgery is not considered a direct comparator but only included as a treatment for patients who failed both biological and nonbiological drug treatments.

Next to ADA, the most frequently included biological treatments are IFX, golimumab, and vedolizumab. Certolizumab pegol and natalizumab are also included in individual studies. In most studies, ADA was administered as follows: induction—160 mg (week 0), 80 mg (week 2); maintenance—40 mg every other week (starting from week 4) [17-19,21,24,26-28]. In other studies, the following treatment schedule was applied: induction—80 mg (week 0), 40 mg (week 2); maintenance—40 mg every other week [20,22,23,25]. The duration of treatment might also be different but is not always clearly stated. Bodger et al [23] included 1 or 2 years of treatment with ADA or IFX, after which patients return to SC. For further information on the treatment schedule of the other interventions and dose escalation, we refer to section 2.3.2 of Multimedia Appendix 1.

### Costs

Most economic evaluations are performed from the perspective of the health care payer. Tang et al [27] took the perspective of a managed care organization in the United States and excluded patient co-payments. Only 2 studies applied a broader societal perspective, including costs related to lost productivity. Loftus et al [25] assumed that each CD-related hospitalization corresponds to a missed interval of work equal to the average duration of serious adverse events (AEs) leading to hospitalization (on average 16.55 days based on the Crohn’s Trial of the Fully Human Antibody Adalimumab for Remission Maintenance (CHARM) trial [29]). This was then multiplied by an 8-hour workday and an average hourly wage in the United Kingdom of £13.00 (US $17.6) [25]. Stawowczyk et al [26] included indirect costs based on an unpublished study carried out in Poland on 202 patients with UC. Indirect costs included absenteeism, presenteeism, and leaving the labor market earlier. Yearly indirect costs for remitted patients counted as PLN 6524 (US $1767) [26]. For patients with active disease, this was PLN 22,935 (US $6211). An overview of the perspective, currency, and year of costing is provided in Multimedia Appendix 1.

#### Costs of Biological Treatments

An overview of the unit costs of biological treatments is provided in Multimedia Appendix 1. It is remarkable that although the unit cost for 40 mg of ADA is lower than 100 mg of IFX in the studies for the United Kingdom [17,21,22] and Canada [18,19], this is the opposite in all US studies [24,27,28]. However, IFX is assumed to be administered in a day-case setting, whereas ADA can be self-administered subcutaneously. As a result, the total treatment costs with IFX are not always lower if administration costs are also taken into account. For example, in the study of Yu et al [28], the total therapy cost for ADA equals the drug costs of US $17,176. For IFX, the total therapy cost of US $18,214 consists of the drug costs (US $14,663), the drug administration costs (US $1605), and excess uninfused drug costs (US $1946). Differences in start-up dose or dose escalation probabilities further influence total treatment costs (Multimedia Appendix 1).

#### Costs of Standard Care

The costs of SC are substantially lower than those of biological treatment costs. In the study by Archer et al [17], SC in the induction phase of 8 weeks, consisting of 5-ASAs, AZA, 6-MP, and prednisolone, costs £167.6 (US $227.4). In the maintenance phase of 26 weeks, this is £343.8 (US $466.4). The same use of background therapies is assumed for all biological treatment arms. Standard care cost differences were small between the different treatment strategies. For example, in their model comparing golimumab with ADA, IFX, or SC, the background therapy costs were £251.43 (US $341.1) per cycle for the standard nonbiological treatment group versus £200.03 (US $271.4) per cycle for the biological treatments during the induction treatment (cycle=8 weeks). During maintenance treatment (cycle=2 months), this was £121.15 (US $164.4) versus £120.98 (US $164.1), respectively [17]. Assasi et al [18] include a total non-anti-TNF outpatient drug costs per cycle (8 weeks) of CAD 116.30 (US $91.6) for drug responsive patients and CAD 85.95 (US $67.7) for drug refractory patients. In the report of Essat et al [21], conventional treatment (balsalazide, mesalazine, olsalazine, sulfasalazine, budesonide, prednisolone, AZA, 6-MP, and MTX) costs £153.6 (US $208.3) per induction cycle (6 weeks) and £204.8 per maintenance cycle (8 weeks). The authors assume that while patients are receiving biological therapy, the costs of conventional therapies are halved (£102.4 [US $138.9]). The same logic is applied in the study of Rafia et al [22], with a cost of £52.62 (US $71.4) per induction cycle and £70.16 (US $95.2) per maintenance cycle, which is halved (£35.08 [US $47.6]) for patients while receiving biological treatment. In the Polish study, standard treatment costs per cycle (8 weeks) are PLN 204 (US $55.3) [26]. Thus, the incremental impact of these costs is minimal in comparison with the biological treatment costs.

#### Costs of Colectomy or Surgery

From the 3 studies including colectomy as an alternative treatment strategy, Archer and Essat refer to information from the study of Buchanan et al [30] to include a cost of £13,452 (US $18,242) [17] and £13,577 (US $18,412) [21] for surgery. In the MSD submission [17], the cost for colectomy is £8968 (US $12,160). Other studies include surgery as an event in their model, without providing further details on the type of hospitalization. A wide range of costs is mentioned: the surgery cost is PLN 12,480 (US $3380) in the Polish study of Stawowczyk et al [26] up to $31,923 for a hospital unit cost in the US study of Yu et al [28]. The cost of surgery was $11,341 in the US study of Kaplan et al [24], £10,581 (US $14,351) in the UK study of Rafia et al [22], and CAD 19,269 (US $15,171) in the CADTH study [19].

### Incremental Costs Related to AEs

Archer et al [17] report that serious and severe AEs were not considered in the AbbVie model. The manufacturer notes that most AEs experienced by patients were nonserious and considered to be unrelated to the study drugs (based on results from the ULTRA2 trial [31]) [17]. In addition, the manufacturer highlights that the exclusion of these events represents a conservative assumption as “the ULTRA2 trial reported slightly higher incidences of serious and severe AEs in the placebo arm than in the adalimumab arm of the trial; therefore, considering serious and severe AEs in the model would have increased medical costs and reduced health gains within the conventional management group” [17]. In addition, the Polish study refers to the ULTRA2 trial [31] to justify that certain AEs were not included in the model because ADA treatment was generally well tolerated and the overall safety profile of ADA was comparable with that of placebo [26]. Rafia et al [22] also indicate that the impact of AEs on the ICER is minimal. Finally, Tang et al [27] mention that they are not aware of evidence that demonstrates large differences in the proportion of adverse drug reactions across the 4 biological treatments (ADA, IFX, certolizumab pegol, and natalizumab), and the frequency of these complications is low. On the basis of their clinical judgment, they conclude that adverse drug reactions should not be included in the structure of the model. Progressive multifocal leukoencephalopathy occurrence with natalizumab was considered a rare but significant AE with a treatment cost between $14,544 (lower limit) and $22,725 (upper limit) [27].

### QoL

Archer et al [17] performed a systematic literature search for utility values. A total of 10 studies reported EuroQol 5-Dimension (EQ-5D) estimates for one or more health states relevant to their model (Multimedia Appendix 1). The authors considered the values reported by Woehl et al [32] and Swinburn et al [33] to be the most useful as “they are UK based, included a fairly large number of patients (n=180 and n=230, respectively) and have the greatest coverage of the health states in the model” [17]. Unfortunately, both studies are only published as an abstract. Swinburn et al [33] included 230 UC patients (postsurgery [n=30], remission [n=78], mild disease [n=47], moderate disease [n=31], and severe disease [n=44]). The EQ-5D utility scores were collected via an online survey. The results are presented in a figure in the abstract, without mentioning the exact utility values. Archer et al [17] extracted utility values from this graph: for patients who had not undergone surgery, the utility scores for each disease severity were as follows: remission 0.91 (95% CI 0.87-0.95), mild disease 0.80 (95% CI 0.70-0.85), moderate disease 0.68 (95% CI 0.58-0.78), and severe disease 0.45 (95% CI 0.35-0.55). Archer et al [17] extracted the following mean EQ-5D scores from the study of Woehl et al [32]: remission 0.87 (SD 0.15), mild disease 0.76 (SD 0.18), and moderate-to-severe disease 0.41 (SD 0.34).

None of the identified economic evaluations is based on an RCT, including a head-to-head comparison of the relevant intervention and comparator, in which utilities are measured with a generic utility instrument. Next to the previously mentioned studies of Woehl et al [32] and Swinburn et al [33], a variety of other sources and assumptions were used in the other identified economic evaluations. We refer to section 2.3.5 of Multimedia Appendix 1 for further details. Most economic evaluations refer to the study of Gregor et al [34] to retrieve relevant utility values. They used the time trade-off (TTO), Standard Gamble (SG), and Visual Analog Scale (VAS) methods in 180 consecutive patients with CD to obtain utilities. All but one of the studies referring to Gregor et al [34] mentioned the use of values from the SG approach [18,24,25,27,28]. Only Dretzke et al [20] used the TTO values. The results for the SG technique were as follows: mild disease, 0.82; moderate disease, 0.73; and severe disease, 0.54 [34]. For the TTO technique, these were 0.96, 0.88, and 0.71, respectively. A second table presented the following mean utility scores for the SG technique at the initial visits: chronically active therapy resistant, 0.74; chronically active therapy responsive, 0.86; acute disease exacerbation, 0.77; remission, 0.88; and overall, 0.81. With the TTO approach, these were 0.88, 0.98, 0.89, 0.96, and 0.92, respectively [34].

Bodger et al [23] mapped the midpoint CD Activity Index (CDAI) scores to EQ-5D utility scores. An algorithm developed by Buxton et al [35] was used (EQ-5D=0.9168−0.0012×CDAI). This algorithm was based on multiple observations from 905 patients with moderate-to-severe CD who participated in the Efficacy of Natalizumab as Active Crohn’s Therapy (ENACT-1) and Evaluation of Natalizumab as Continuous Therapy (ENACT-2) clinical trials [36]. We refer to our discussion on QoL for some critical remarks from the authors who developed this algorithm.

Finally, large differences are observed in the postsurgery remission utility values. Several studies assign a value equal [18] or similar [24,27] to the utility for (medical) remission. In contrast, several other studies assign a much lower value for postsurgery remission. For example, in the MSD submission, the utility value for postcolectomy remission (0.60) was assumed to be equal to the utility value for late complications (postcolectomy). Similar values for postsurgery remission were assumed in the study discussed in the CADTH report (0.67) [19] and in the studies by Essat et al (0.60) [21], Rafia et al (0.57) [22], and Stawowczyk et al (0.61) [26], whereas remission utility values were much higher—0.82, 0.86, 0.82, and 0.88, respectively. Multimedia Appendix 1 provides further details on the utility values assigned to (post-)surgery health states. We come back to the observed differences in the *Discussion* section.

### Treatment Effect

The treatment effect of the included studies was based on a wide range of sources. An overview of the trials is provided in Multimedia Appendix 1. Several authors conducted a network meta-analysis for both the induction and maintenance phases [17,21,22]. Other studies performed an indirect comparison. For example, in the study of Assasi et al [18], the initial remission and response rates for IFX were derived from the 12-week results of the 5 mg/kg arm that was reported by Targan et al [37]. For ADA, the 4-week results of the 160 mg and 80 mg arm of the CLASSIC 1 study were used [38]. For the usual care strategy, pooled rates from the placebo arms of these 2 trials were used to estimate remission and response rates. Bodger et al [23] and Dretzke et al [20] selected the ACCENT I trial [39] to model the IFX arm and the CHARM trial [29] for ADA. This was also the case in the study by Kaplan et al [24]. However, in the latter study, the initial response rate to ADA was retrieved from the GAIN study [40] that evaluated ADA induction following IFX failure. In addition, the CADTH report [19] referred to an indirect treatment comparison conducted by the manufacturer to estimate the efficacy of treatments for inducing response or remission. Although most studies refer to the CHARM trial [29] for information on ADA, Stawowczyk et al [26] referred to the ULTRA 2 study [31] for estimates of response and remission with ADA or SC.

Yu et al [28] relied on data from the CHARM [41] and ACCENT I [42] trials to model results for the ADA and IFX treatment arms. The authors remark that patient samples were not equivalent at baseline. The CHARM trial included patients with a maximum baseline CDAI score of 450 versus 400 for ACCENT I [28]. Therefore, the sample of 234 ADA-treated patients was weighted to have the same baseline median and the same 25th and 75th percentile CDAI values, sex distribution, and median age as those in the IFX arm of ACCENT I [28]. No such adjustments were made in the other indirect comparisons. Furthermore, strong assumptions are made. For example, Archer et al [17] point out that in the MSD submissions, “patients who have previously achieved a response can either maintain or lose that response, but they cannot improve (i.e. they cannot subsequently transit to the remission state). ...no additional patients can achieve remission after induction and no patients with remission can completely lose response during any given model cycle.”

### Incremental Cost-Effectiveness Ratios

In this overview, we focus on the results of treatment with ADA and other biologicals (IFX, golimumab, and vedolizumab). For a more detailed overview, we refer to sections 2.3.8 and 2.3.9 of Multimedia Appendix 1. Section 2.3.8 of Multimedia Appendix 1 also includes overview tables presenting the results of the identified economic evaluations.

In the study by Archer et al [17], when colectomy is an alternative, colectomy is expected to dominate IFX, ADA, golimumab, and conventional nonbiological treatments. When elective colectomy is not an acceptable or preferred option, IFX and golimumab are expected to be ruled out because of dominance. The ICER of ADA versus conventional nonbiological treatment is expected to be approximately £50,300 (approximately US $68,200) per QALY gained [17]. In the AbbVie submission (marketing ADA–Humira), ADA had an ICER of £34,590 (approximately US $46,900) per QALY gained. In contrast, in the MSD submission (marketing IFX or Remicade and golimumab or Simponi), ADA is expected to be dominated by golimumab [17].

Assasi et al [18] calculated that usual care created the lowest expected QALYs. However, the costs associated with ADA and IFX could be perceived as high with ICERs of about CAD 193,000 (approximately US $152,000) per QALY and CAD 451,000 (approximately US $355,000) per QALY, respectively.

According to Bodger et al [23], IFX was always more expensive and less effective than ADA, for which their model suggests acceptable ICERs of less than £14,000 (approximately US $19,000) per QALY [23].

CADTH evaluated the manufacturer’s pharmacoeconomic evaluation. According to the manufacturer’s calculations, golimumab has an ICER of about CAD 42,000 (approximately US $33,000) per QALY and other biosimilars are (extendedly) dominated. However, the Common Drug Review identified several issues in the indirect treatment comparison and assumptions that might bias the results in the favor of golimumab [19].

Dretzke et al [20] did not mutually compare ADA and IFX. For induction therapy, both biosimilars dominated SC in the management of severe CD. For moderate CD, only ADA was dominant relative to SC. Neither drug was considered cost-effective as maintenance therapy with ICERs of about £5 million (approximately US $6.8 million) per QALY and £14 million (approximately US $19 million) per QALY for ADA and IFX, respectively [20].

According to the manufacturer’s analysis reviewed by Essat et al [21], vedolizumab dominates surgery, IFX, and golimumab. Compared with ADA, the ICER of vedolizumab is estimated at £6634 (US $8990) per QALY. In contrast, according to the Evidence Review Group (ERG), surgery is likely to dominate all medical treatments. If surgery is not an option, the review group indicates that vedolizumab is expected to be dominated by ADA [21].

Kaplan et al [24] estimated that IFX dose escalation yielded 0.03 extra QALYs compared with the ADA strategy. However, in combination with an extra cost of more than $10,000, this results in an ICER of more than $330,000 per QALY [24].

In comparison with nonbiological pharmacotherapy, Loftus et al [25] calculated ADA has an ICER of about £16,000 (approximately US $21,700) per QALY and £34,000 (approximately US $46,000) per QALY in the treatment of severe or moderate-to-severe CD, respectively [25].

In the study by Rafia et al [22], ADA provided 0.21 additional QALYs in comparison with conventional nonbiological therapy for an additional cost of £4000 (approximately US $5400), resulting in an ICER of about £19,000 (approximately US $25,700) per QALY. Vedolizumab was extendedly dominated. IFX provides 0.0383 additional QALYs in comparison with ADA for an additional cost of approximately £4400 (approximately US $6000), leading to an ICER of almost £116,000 (approximately US $157,000) per QALY [22].

In addition, the study of Stawowczyk et al [26] indicated that ADA is more effective and more costly than SC. One-year ADA treatment results in an ICER of €71,000 (approximately US $86,700) or €76,000 (approximately US $92,800) per QALY, depending on the perspective [26].

Tang et al [27] compared several treatments after SC failed. No significant differences in efficacy were calculated between the 4 biological treatments (IFX, ADA, certolizumab pegol, and natalizumab). They produce similar QALYs with overlapping 95% CIs. On the basis of Monte Carlo simulations, IFX had the highest probability of being the most cost-effective therapy compared with the other biological treatment options [27].

In contrast with the previous study, Yu et al [28] calculated that ADA delivers more QALYs and saves approximately $4900 in comparison with IFX. On the basis of the probabilistic analysis, ADA dominates IFX in approximately 94% of the simulations and is the preferred biological treatment option [28].

### Uncertainty

Almost all studies performed both probabilistic sensitivity analysis and scenario analyses or one-way sensitivity analyses to estimate the uncertainty surrounding estimates of incremental costs, incremental effects, and ICERs. Only Kaplan et al [24] did not perform a probabilistic sensitivity analysis.

Multimedia Appendix 1 gives an overview of the most determining variables, as indicated by the authors of the original economic evaluations. The most often mentioned variables are the treatment effect [18,20,21,24,27], utilities [17,21,22,27], and time horizon [18-23,25]. Some authors also highlight the importance of the treatment duration [21,23,26], drug treatment costs [24,27], health state costs [17,21], and patient weight [18].

## Discussion

An overview of the economic literature allows us to identify important issues related to (the calculation of) the cost-effectiveness of ADA. A major strength is that this exercise was performed before the trial was started. This way, we avoid that important information to allow the performance of a high-quality economic evaluation was not measured in the trial. In the first part of this discussion, issues identified from the review of the economic literature relevant to the REDUCE-RISK trial are discussed. On the basis of these issues and expert opinion, input is provided to ameliorate the protocol of the REDUCE-RISK trial from a health economic point of view. The added questionnaires are discussed in the second part of this discussion.

### Issues Identified in the Economic Literature Review Related to the REDUCE-RISK Trial

#### Pediatric Population

All the identified economic evaluations performed an analysis for an adult population. Two studies also included a secondary analysis for the pediatric population [17,20]; however, efficacy data still relied on trials that included only an adult population. The analysis also did not include youngest children. Archer et al [17] reported that patients’ starting age in their pediatric population was 15 years. The lack of information related to the treatment effect of biologicals in pediatric patients means that the results of such secondary analyses should be interpreted with caution. Archer et al [17] suggested RCTs assessing the clinical effectiveness of biologicals in pediatric patients as a research priority. This makes the conduct of the REDUCE-RISK trial, which includes patients aged 6 to 17 years, very worthwhile.

#### Severity of Disease

Almost all studies explicitly include a population with moderate-to-severe CD or UC disease. Only two reports differentiate results according to disease severity. Dretzke et al [20], inclusive of the Abbott submission discussed in the same report, distinguish between severe and moderate disease. Loftus et al [25] performed calculations for severe CD and moderate-to-severe CD. They did not perform a separate analysis for the moderate CD patients. Such a distinction is important in economic evaluations as applying the same relative treatment effect to a higher baseline risk for a specific event results in a larger absolute treatment effect. The severity of disease might have a significant impact on an intervention’s ICER. Making an explicit distinction in the REDUCE-RISK trial between patients at low or high risk for aggressive disease course is desirable from both clinical and economic points of view.

#### Adalimumab Versus Other Biological Treatment Options

In most of the identified economic evaluations, ADA has a better cost-effectiveness than the other biologicals. In the study by Archer et al [17], IFX and golimumab are expected to be ruled out in the economic analysis because of dominance (less effective and more expensive), whereas the ICER of ADA versus conventional nonbiological treatment is expected to be approximately £50,300 (approximately US $68,200) per QALY gained [17]. In the study by Assasi et al [18], the ICER of ADA versus usual care is relatively high (approximately CAD 193,000 [US $152,000] per QALY); however, this is even higher for IFX versus ADA (CAD 451,000 [approximately US $355,000] per QALY). In the study by Bodger et al [23], IFX is dominated by ADA.

In the study by Dretzke et al [20], IFX and ADA are not mutually compared. However, the findings of the economic model were in favor of ADA: for induction, both ADA and IFX were cost-effective (dominant relative to SC) in the management of severe CD, and ADA was cost-effective for moderate CD (dominant relative to SC) [20].

In the study by Essat et al [21], according to the ERG group, vedolizumab is expected to be dominated by ADA if surgery is not an option [21].

The results of the study by Rafia et al [22] indicate that, assuming a cost-effectiveness threshold of £30,000 (approximately US $40,000) per QALY, ADA has the highest probability of being the most cost-effective intervention (78%). Similarly, in the study by Yu et al [28] based on the probabilistic analysis, ADA dominates IFX in approximately 94% of the simulations.

In the US study of Tang et al [27], IFX and ADA are about equally effective and IFX is cheaper. Finally, Loftus et al [25] and Stawowczyk et al [26] only compared ADA with conventional nonbiological treatment.

On the basis of the aforementioned information, most of the economic studies were in favor of ADA in comparison with other biologicals. Only in the manufacturer’s submissions, the conclusion is different. For example, in the MSD submission [marketing IFX or Remicade and golimumab or Simponi), ADA is expected to be dominated by golimumab [17]. However, the manufacturer’s submission includes a discount for the drug. If this discount is not taken into account, golimumab is ruled out because of extended dominance. Similarly, in a Canadian study, according to the manufacturer’s calculations, golimumab has an ICER of approximately CAD 42,000 (approximately US $33,000) per QALY and IFX and ADA are (extendedly) dominated [19].

Most of the identified studies indicate that ADA has a better cost-effectiveness than the other biologicals included in the analyses, and thus, from a health economic point of view, it seems to be a justified biological intervention in future trials. Nevertheless, in economic evaluations, it is important to work on the efficiency frontier, that is, comparing treatments with the next best non-(extendedly) dominated intervention. From a health economic point of view, it is important to make an incremental analysis and include SC in the analysis and not immediately compare biologicals with each other. As mentioned by Dretzke et al [20], this would only be relevant “where both adalimumab and infliximab have been first justified as maintenance therapies versus standard care (SC). Where one or both maintenance therapies are not cost-effective versus SC, this comparison provides no information to decision-makers.” Therefore, from a health economic point of view, we considered it justified to compare methotrexate with ADA in the high-risk patient group of the REDUCE-RISK trial.

#### Treatment Effect

The input for the conventional nonbiological, ADA, and IFX treatment is often based on the CLASSIC I [43], CHARM [29], and ACCENT I [39,42] trials, respectively. However, comparing outcomes from individual treatment arms of separate trials might bias the results, and the direction of this bias is unknown. Head-to-head RCTs are needed to allow an unbiased comparison of biological therapy with SC. It is also necessary to set up reliable health economic models to estimate the intervention’s cost-effectiveness. The REDUCE-RISK trial is an example of such a head-to-head RCT.

#### QoL

The EUnetHTA guideline for methods for health economic evaluations recommends that results be presented in terms of both a cost-effectiveness analysis and a cost-utility analysis [44]. The primary outcome measures should, where appropriate, be presented as natural units (including life years) and as QALYs [44]. The health-related quality of life (HRQoL) aspects of the QALY were captured in a HRQoL weight. On the basis of the review of guidelines used by EUnetHTA partners, EQ-5D is the most commonly recommended instrument for deriving HRQoL weights, although other instruments are also mentioned (eg, Health Utility Index, Short-Form 6-Dimension, or 15-dimensional) [44].

A major limitation is that none of the underlying trials measured QoL with a generic utility instrument. As a result, the authors of the economic evaluations have to make many assumptions in their models. Previous reviewers also noticed strange assumptions regarding utility values that are linked to health states. For example, Essat et al [21] reported that the utility value in postsurgical remission was lower than for moderate or severe disease (0.60 vs 0.68), which appears to be inconsistent. Bodger et al [23] transformed CDAI scores to utilities, based on an algorithm developed by Buxton et al [35]. In this study, the correlation between CDAI and EQ-5D is −0.62, and 29% of the variability in EQ-5D scores is explained by CDAI [35]. However, Buxton et al [35] mention in their discussion that “based on the variance explained, the relationships between the CDAI and utilities in the simple models are weaker than those for the IBDQ [Inflammatory Bowel Disease Questionnaire] and suggest that the CDAI provides a poorer basis for estimating utilities. Again its relatively poor performance as a predictor of utility reflects its main role as clinical indicator of disease activity, rather than of HRQoL*.”* In the absence of utility values for surgery, Dretzke et al [20] assumed that this health state is represented by the EQ-5D state 22222, which has a UK utility weight of 0.516. Such assumptions are arbitrary and not very reliable. As the model results are sensitive to such utility assumptions, better evidence-based input is desirable. In the REDUCE-RISK trial, this is taken into account by including the EQ-5D questionnaire in the research protocol (see the second part *EQ-5D* of this discussion).

#### Indirect Costs

Finally, the studies are performed from a health care payer’s perspective, which excludes indirect nonhealth care related costs, such as costs related to lost productivity. In contrast, indirect costs would represent a substantial portion of the costs of CD. A US study indicates that this accounts for 28% of the total CD cost in the United States [45]. Only 2 studies [25,26] included a scenario with the inclusion of these costs. In the Polish study, based on an unpublished study, yearly indirect costs for remitted patients counted to PLN 6524 (US $1767). This was PLN 22,935 (US $6211) for patients with active disease. Loftus et al [25] indicate that “including indirect costs related to lost productivity due to hospitalization improved the cost-effectiveness of adalimumab therapy. However, the estimate of indirect costs was likely substantially underestimated because only work missed during hospitalization was included. Other indirect costs, such as decreased productivity at work and labor force nonparticipation, were not included*.*” Assasi et al [18] also reported that if a societal perspective was taken and indirect costs were included in the model, the cost-effectiveness of anti-TNFs compared with that of usual care likely would have been lower. In addition, Yu et al [28] claim that reliable data sources to include the impact on indirect costs are lacking. Efforts should be taken to gather reliable information about the impact of different treatments on indirect costs. In the next part of this discussion, we discuss how measures for school attendance and parents’ productivity are included in the research protocol of the REDUCE-RISK trial.

### Added Elements in the REDUCE-RISK Trial Research Protocol

As recommended by the EUnetHTA guidelines on HRQoL [46], future studies should include a generic utility instrument in complement to disease-specific questionnaires to adequately capture the impact of a disease on daily life. Including a generic utility instrument in further research is also suggested as a research priority by the reviewers in the study by Archer et al [17] and Dretzke et al [20] and the underlying NICE report [16]. The disease-specific IMPACT-3 HRQoL measure was already included in the protocol. On the basis of the aforementioned data, a generic utility instrument (EQ-5D) is added to the research protocol. The ISPOR guidelines also recommend “prioritization of high-cost resources as well as those that are expected to differ between treatment arms, without distinction as to whether they are related to disease or intervention [47]. The scope of resources considered should include direct medical and nonmedical resources and indirect or productivity costs across patients and caregivers.” [4]. Similar to previous economic evaluations, differences in treatment costs and costs related to AEs will be taken into account. In addition, in the REDUCE-RISK trial, children’s school attendance and parents’ productivity will also be measured.

#### EQ-5D

The economic literature review identified the lack of QoL data that could be expressed as utilities and also indicated this as a research priority. Following the EUnetHTA guidelines on HRQoL [46], such information will be included in the REDUCE-RISK trial through the inclusion of the generic EQ-5D questionnaire.

In patients with CD and UC, a study by Stark et al [48] showed that both the EQ-VAS (EuroQol-visual analog scale) and EQ index scores correlate well with disease activity indices and differ significantly between active disease and remission groups. The authors concluded that the EQ-5D generates valid, reliable, and responsive preference-based evaluations of health in CD and UC. The EQ-VAS scores were more responsive than EQ-5D index scores, and thus, small health differences that are important from the patient's perspective may not be reflected in the EQ index [48]. This is in line with the results from a previous study from this research group that also concluded the EQ-5D to be “reasonably valid, reliable and responsive in patients with inflammatory bowel disease. It can be used to generate preference-based valuations of health-related quality of life in inflammatory bowel disease.” [49].

From a practical point of view, the time for completion is less than 2 min for the EQ-5D [50]. From a financial point of view, the EQ-5D could be used free of charge for this study.

Three EQ-5D questionnaires are included: the EQ-5D-Y (youth version), EQ-5D-Y proxy1, and EQ-5D-5L.

1. The EQ-5D-Y was administered to measure the children’s QoL. Following the user’s guidelines, the youth version is used for all patients included in the REDUCE-RISK trial: “A study only with children up to 18 years, in this case EQ-5D-Y for older children would be recommended in order to have only one EQ-5D version in the study. The switch-over to the adult version could bring discontinuity as the adult and child versions are two different instruments.” [51].
2. In the youngest children (<8 years), it is not possible to apply a self-completing questionnaire. We ask one of the parents to fill in the proxy version. The proxy rates how he or she rates the child’s health. “The use of proxies, such as caregivers or family, should be avoided where possible. However, the use of proxies for the measurement of HRQoL is unavoidable in some cases, e.g. cognitively impaired patients, small children.” [46]. By asking this for all patients, we will be able to evaluate the agreement between self- and proxy-reports of the EQ-5D-Y questionnaire.
3. The EQ-5D-5L was used to measure parents’ QoL. It is important to find ways of incorporating relatives’ costs and effects when these might be substantial and may influence the ICERs [52]. Parents’ QoL of children with CD or UC might be such an example. This has not been included in any of the identified studies, and thus, the impact is unclear. Davidson et al [52] stated that the most relevant outcome measure to use for relatives’ effects would be their affected utility. Therefore, we also included the measurement of parents’ QoL through the use of the EQ-5D questionnaire. There is a choice between the 3L and 5L versions. The EQ-5D-5L version might be more sensitive to changes in health status in comparison with the 3L version [53,54]. Schwenkglenks et al [55] expect that the 5L version will gradually replace the 3L version, because of reduced ceiling effects and more appropriate responsiveness. Goldsmith et al [56] also referred to the increased ability to discriminate health states, which may improve the prediction of EQ-5D index values. Therefore, the EQ-5D-5L version was used.

The QoL measurements were made at baseline and all following planned study visits (months 2, 4, 6, 9, and 12). More information about the EQ-5D questionnaires, the included language version, the available value sets, and a sample version is available in section 3.1 of Multimedia Appendix 1.

#### School Attendance

A review of the economic literature indicates that indirect costs might represent a substantial portion of costs related to CD and UC but that the impact of different treatment options on such costs is lacking. As the patient population in the REDUCE-RISK trial is restricted to children and adolescents aged 6 to 17 years, indirect costs do not immediately relate to the patient’s productivity. Instead, we try to measure the impact on a patient’s school attendance.

Three studies were identified measuring the impact of CD or UC on school performance [57-59]. One study used a semistructured questionnaire for both children and parents and found that significant psychosocial and academic difficulties are faced by children with chronic diseases such as IBD [57]. Children with CD and UC missed significantly more school days than age-matched healthy controls [57]. Another study [58] created an online survey that included a Student Adaptation to College Questionnaire (SACQ). The results show that “disease activity in students with CD was associated strongly with their self-reported ability to keep up with academic work (*P*<.0089) and confidence in their ability to meet future academic challenges (*P*<.0015). Students with active IBD reported feeling as if they were not academically successful (*P*<.018), and students with ulcerative colitis reported irregular class attendance (*P*<.043).” [58]. The third study obtained report cards and school absence information from schools. Children with IBD had poorer school functioning and significantly more absences [59]. None of these studies used a structured questionnaire that was validated for use in children with CD or UC. The SACQ questionnaire is a 67-item, self-report questionnaire that is for college students and is mainly used at universities for routine freshman screening. This is considered inappropriate for our research.

An additional nonsystematic Google search was performed to identify other potentially relevant questionnaires. However, these questionnaires are very general. For example, the *School Attendance Questionnaire* mentions that these questionnaires are generally designed by school authorities to find out the reasons for missing school. However, the questions posed clearly indicate that this questionnaire of school attendance is not well placed to apply in a population of sick children (eg, “Are your parents aware of this attendance percentage?” or “Are you aware that ...can lead to your suspension from school?”). Other researchers proposed a novel method for measuring class attendance by using location and Bluetooth data collected from smartphone sensors [60]. This is not applicable for the youngest children in our population because they usually do not have a smartphone.

No well-suited questionnaire was thus identified that can be used for the international REDUCE-RISK trial. Therefore, a de novo school attendance questionnaire was set up. Limitations of this questionnaire are that it is nonvalidated and that we cannot rely on the experience of other researchers with this questionnaire. Nevertheless, the choice was made to use this new instrument because we preferred to take the initiative to try to measure the impact with a nonvalidated instrument instead of not trying to measure this important aspect. The school attendance questionnaire consists of a version that is used at the first visit and a version to be used at the follow-up visits.

The parents filled in the questionnaire. First, we asked them to give a general picture of a typical school week to be able to have a view on the number of days the child goes to school in a typical week (exclusive home education) and the presence of home schooling (or home education). The aim of the questionnaire is to estimate the impact of IBD (CD and UC) and its treatment on school attendance and home education. The questions are related to the following: the presence and amount of home education; whether home education is because of IBD; the percentage of school days that children could not attend; in the case of home education, the percentage of home schooling days that children could not attend; and for both school days and home education, the part of absence that is because of IBD (in the opinion of the parents).

To assist participants with accurate recall, the ISPOR guidelines [4] recommend economic investigators to consider using memory aids such as diaries to record medical visits and events. Investigators should inform participants that they will be asked to report this information throughout the trial [61]. In line with this recommendation, the last page of the questionnaire, entitled *Information for parents to take home to help in collecting information for the next follow-up visit*, contains an overview of the questions.

Further details on the timing of the measurements and a sample version of these questionnaires are available in section 3.2.1 of Multimedia Appendix 1.

#### Parents’ Productivity

To improve the quality and uniformity of data generated from trials, the ISPOR guidelines [4] recommend using validated instruments when incorporating productivity costs [62-64]. The Work Productivity and Activity Impairment (WPAI) questionnaire [65] is a self-administered questionnaire assessing the impact of a disease on a patient's ability to work and/or perform nonwork activities. A version exists specifically for CD (WPAI:CD), and a caregiver version (WPAI:CD-CG) exists in which the effect of a child's specific health problem on the parent's work productivity is measured. This WPAI:CD-CG questionnaire is included in the REDUCE-RISK trial. The included questions are related to the following: Q1: current employment, Q2: hours missed because of problems associated with the child’s CD, Q3: hours missed for other reasons, Q4: hours actually worked, Q5: degree child’s CD affected productivity while working, and Q6: degree child’s CD affected regular activities. The questionnaire is available at no charge in several languages. Details about the scores that will be calculated from these questions and the timing of measurements are available in section 3.2.2 of Multimedia Appendix 1.

### Conclusions

This paper addresses an important and progressive issue: including health economic considerations in the design of clinical trials. At the end of the trial, when all information on the intervention’s efficacy and safety has been gathered, the important incremental variables will be combined in a trial-based economic evaluation calculating the intervention’s incremental costs, effects, and ICERs, both from a health care payer and societal perspective. Guidelines for performing economic evaluations will be followed. For example, parameter uncertainty will be included by performing a probabilistic sensitivity analysis. Following the ISPOR guidelines, reporting of the methods and results of the economic evaluation will be performed according to the CHEERS guidelines [11,12].

In conclusion, we are of the opinion that performing a systematic literature review supports researchers in setting up a research protocol. In our case, the results of the literature review helped us to identify important variables for which evidence should be gathered in the REDUCE-RISK trial to allow the performance of a high-quality economic evaluation.

## Appendix

Multimedia Appendix 1 Paediatric inflammatory bowel diseases network for safety, efficacy, treatment and quality improvement of care (PIBD-SETQUALITY): economic evaluation considerations.

Multimedia Appendix 2 Included and excluded studies (and reasons for exclusion).

Multimedia Appendix 3 PRISMA-2009-Checklist (literature review economic part REDUCE-RISK trial).

## Abbreviations

6-MP: 6-mercaptopurine
ADA: adalimumab
AE: adverse event
AZA: azathioprine
CADTH: Canadian Agency for Drugs and Technologies in Health
CD: Crohn disease
CHEERS: Consolidated Health Economic Evaluation Reporting Standards
CRD: Centre for Reviews and Dissemination
EEN: exclusive enteral nutrition
EQ-VAS: EuroQol-visual analog scale
ERG: Evidence Review Group
HTA: health technology assessment
IBD: inflammatory bowel disease
ICER: incremental cost-effectiveness ratio
IFX: infliximab
ISPOR: International Society for Pharmacoeconomics and Outcomes Research
MTX: methotrexate
NICE: National Institute for Health and Care Excellence
QALY: quality-adjusted life years
QoL: quality of life
SC: standard care
UC: ulcerative colitis
