# Supplementary material for: Identifying Health Economic Considerations to Include in the Research Protocol of a Randomized Controlled Trial (the REDUCE-RISK Trial): Systematic Literature Review and Assessment
Source: JMIR Form Res. 2021 Jan 25;5(1):e13888. doi: 10.2196/13888 (PMC7870354; doi:10.2196/13888)
Supplement: Multimedia Appendix 1 [file formative_v5i1e13888_app1.pdf]

PAEDIATRIC INFLAMMATORY BOWEL DISEASES NETWORK FOR SAFETY, EFFICACY, TREATMENT AND QUALITY IMPROVEMENT OF CARE / PIBD-SETQUALITY

ECONOMIC EVALUATION CONSIDERATIONS

MATTIAS NEYT, ANNICK CHRISTIAENS, GIGI VEEREMAN

Mattias Neyt, senior health economist, ME-TA Medical Evaluation and Technology Assessment (Merendree, Belgium) and Belgian Health Care Knowledge Centre (Brussels, Belgium)

Annick Christiaens, study nurse, UZ Brussel – University Hospital Brussels (Brussels, Belgium)

Gigi Veereman, Pediatric Gastroenterology and Nutrition, UZ Brussel (University Hospital Brussels) - Vrije Universiteit Brussel (Brussels, Belgium)

ME-TA
Medical Evaluation
Technology Assessment

Contact details:
Mattias Neyt
Jan Burssensstraat 23
9850 Merendree
Belgium
+32 (0)477/90.13.17
mattias.neyt@me-ta.eu

Short title

Contents

1 INTRODUCTION 10
1.1 HEALTH PROBLEM 10
1.2 REDUCE-RISK TRIAL 11
1.2.1 RCT 11
1.2.2 Inception cohort and safety registry 13
1.3 IMPORTANCE OF ECONOMIC CONSIDERATIONS 14
2 ECONOMIC LITERATURE REVIEW 16
2.1 LITERATURE SEARCH 16
2.1.1 Search strategy 16
2.1.2 Selection criteria 16
2.2 RESULTS OF THE ECONOMIC SEARCH STRATEGY 17
2.3 OVERVIEW OF ECONOMIC EVALUATIONS 21
2.3.1 General information 21
2.3.2 Population and compared interventions 23
2.3.3 Costs 27
2.3.4 Adverse events 32
2.3.5 Quality of life 32
2.3.6 Treatment effect: underlying trials 40
2.3.7 Uncertainty 44
2.3.8 Results 45
A) Base-case results 45
B) Sensitivity analysis 51
2.3.9 Authors' conclusions 55
2.4 DISCUSSION 57
3 ECONOMIC INPUT FOR THE RESEARCH PROTOCOL 63
3.1 EQ-5D 64
3.1.1 EQ-5D-Y 65
3.1.2 EQ-5D-Y proxy1 66

3.1.3 EQ-5D-5L 67
3.1.4 Languages 67
3.1.5 timing 69
3.1.6 Presenting EQ-5D results and value sets 69
A) EQ-5D-5L 70
B) EQ-5D-Y (proxy1) 71
3.2 School attendance and productivity questionnaires 72
3.2.1 School attendance 72
3.2.2 Parents' productivity 75
3.3 Treatment costs 79
3.4 Adverse events and related costs 80
3.5 Statistical analysis 81
3.6 Economic modelling 82

4 REFERENCES 84

5 APPENDICES 93

Appendix 1. Search strategy economic evaluations 93
Appendix 2. Data extraction sheet 100
Appendix 3. EQ-5D questionnaires – sample versions 101
Appendix 4. De novo school attendance questionnaire 104
Appendix 5. Abandoned productivity questionnaire 106
Appendix 6. WPAI:CD-CG 108

List of figures

Figure 1: the REDUCE-RISK randomised controlled trial design 12
Figure 2: Selection of relevant articles 18

List of tables

Table 1: Economic evaluation selection criteria 17
Table 2: List of selected economic evaluations 19
Table 3: General information on the identified economic evaluations 22
Table 4: Population and compared interventions in the identified economic evaluations 26
Table 5: Study perspective, currency and year of costing in the identified economic evaluations 27
Table 6: Unit costs of biological treatments 30
Table 7: Biological treatment costs 31
Table 8: Characteristics and findings of studies included in the systematic review of utility values 34
Table 9: Utility values assigned to different health states in the identified economic evaluations 38
Table 10: Utility values assigned to (post-)surgery health states and complications/AEs 40
Table 11: Sources for the modelled treatment effect 43
Table 12: Type of analysis used to handle uncertainty 44
Table 13: Results presented in the identified economic evaluations (part 1/3) 49
Table 14: Results presented in the identified economic evaluations (part 2/3) 50
Table 15: Results presented in the identified economic evaluations (part 3/3) 51
Table 16: Most determining variables according to performed sensitivity analyses in the identified economic evaluations 54
Table 17: Overview of available EQ-5D questionnaires 68
Table 18: Overview of the EQ-5D measurements (RCT and inception cohort) 69
Table 19: Overview of the school attendance measurements (RCT and inception cohort) 74

Table 20: Overview of available WPAI:CD-CG questionnaires (Crohn's Disease, for caregivers) 76
Table 21: Overview of available WPAI:UC-CG questionnaires (Ulcerative Colitis, for caregivers) 77
Table 22: Overview of the WPAI:CD-CG measurements (RCT and inception cohort) 78
Table 23: List of INAHTA member websites searched for HTA reports 93
Table 24: Search strategy and results for CRD HTA 95
Table 25: Search strategy and results for CRD NHS EED 96
Table 26: Search strategy and results for Medline (OVID) (part I) 96
Table 27: Search strategy and results for Medline (OVID) (part II) 97
Table 28: Search strategy and results for EMBASE 97
Table 29: Results of search strategy 99
Table 30: Data extraction sheet 100

Abbreviations

5-ASAs	5-aminosalicylates
6-MP	6-mercaptopurine
ADA	Adalimumab
AE	Adverse event
AZA	Azathioprine
BSA	Body surface area
CAD	Canadian dollars
CADTH	Canadian Agency for Drugs and Technologies in Health
CD	Crohn's disease
CDAI	Crohn's Disease Activity Index
CDR	Common Drug Review
CEAC	Cost-effectiveness acceptability curve
CER	Certolizumab pegol
CHEERS	Consolidated Health Economic Evaluation Reporting Standards
CoI	Conflict of interest
Conv.	Conventional non-biologic therapies
CRD	Centre for Reviews and Dissemination
CRP	C reactive protein
CUA	Cost-utility analysis
ECCO	European Crohn's and Colitis Organization
eCRF	Electronic case report forms
EED	Economic Evaluation Database
EEN	Exclusive enteral nutritional
EN	Enteral nutritional
eow	Every other week
EQ-5D	EuroQol 5-Dimension Quality of Life Questionnaire
ERG	Evidence Review Group
ESPGHAN	European Society for Paediatric Gastroenterology, Hepatology, and Nutrition
GOL	Golimumab
HRQoL	Health-related quality of life
HTA	Health Technology Assessment

IBD	Inflammatory bowel disease
IBDQ	Inflammatory Bowel Disease Questionnaire
IC	Incremental cost
ICER	Incremental cost-effectiveness ratio
ICUR	Incremental cost-utility ratio
IE	Incremental effect
IFX	Infliximab
INAHTA	International Network of Agencies for Health Technology Assessment
IPAA	Ileal pouch anal anastomosis
iPCQ	iMTA Productivity Cost Questionnaire
IRB	Institutional Review Board
ISPOR	International Society for Pharmacoeconomics and Outcomes Research
ITC	Indirect treatment comparison
ITT	Intention-to-treat
LOCF	Last-observation-carried-forward
MeSH	Medical Subject Headings
MTX	Methotrexate
NA	Not available
NAT	Natalizumab
NHS	National Health Service
NICE	National Institute for Health and Care Excellence
NMA	Network meta-analysis
PCDAI	Paediatric Crohn's Disease Activity Index
PEDDCReN	Paediatric European Digestive Diseases Clinical Research Network
PGA	Physician global assessment
PIBD	Paediatric onset Inflammatory Bowel Diseases
PIBD-NET	Pediatric inflammatory bowel diseases network
PLN	Polish zloty
PML	Progressive multifocal leukoencephalopathy
PROBE	Prospective randomized open blind end-point
PSA	Probabilistic sensitivity analysis

PSS	Personal Social Services
QALY	Quality-adjusted life year
RCT	Randomized controlled trial
REA	Relative effectiveness assessment
SACQ	Student Adaptation to College Questionnaire
SAE	Serious adverse events
SAP	Statistical Analysis Plan
SC	Standard care
ScHARR	School of Health and Related Research
SG	Standard gamble
SME	Small/medium-size enterprise
Surg.	Surgery
TAI	Total activity impairment
TNF-α	Tumour necrosis factor-alpha
TPMT	Thiopurine-methyl transferase
TTO	Time trade-off
TWPI	Total work productivity impairment
UC	Ulcerative colitis
VAS	Visual analogue scale
VED	Vedolizumab
WePP	Western preference pattern
WPAI	Work Productivity and Activity Impairment questionnaire
WPAI:CD	WPAI: Crohn’s Disease
WPAI:CD-CG	WPAI:CD-Caregiver version
wPCDAI	Weighted Paediatric Crohn's Disease Activity Index
WTP	Willingness-to-pay

1 INTRODUCTION

The “*Risk-stratified randomized controlled trial in paediatric Crohn’s Disease: Methotrexate versus azathioprine or adalimumab for maintaining remission in patients at low or at high risk for aggressive disease course, respectively – a treatment strategy (REDUCE-RISK)*” trial has received funding from the European Union’s Horizon 2020 research and innovation programme under grant agreement No 668023. This trial has been reviewed and approved by the national Institutional Review Boards (IRB) of participating countries and is prospectively registered (ClinicalTrials.gov Identifier: NCT02852694[a], date of registration: 09/06/2016, EudraCT Number: 2016-000522-18).

The aim of this report is to transparently provide information about the economic part that is added to this study so that patients, researchers, policy makers, and other stakeholders already know what they can expect in the future from the economic evaluation that will be performed alongside this trial.

Prior to providing further details about the economic section, a brief overview regarding the disease is given (part 1.1) and the REDUCE-RISK trial (part 1.2). We note that this information is copied unchanged from the full protocol and also refer to this protocol for more detailed information.

> “*The days of deciding whether or not summary results are worth reporting are over: all such trials will have summary results information posted publicly on ClinicalTrials.gov. The time to decide whether a trial is worth doing is before the trial is started, not after participants have been put at risk.*”[1]

1.1 HEALTH PROBLEM[b]

Crohn’s disease is a chronic recurrent inflammatory disorder, which can cause tissue and bowel damage leading to major disability if not treated adequately. The recent guidelines from the European Crohn's and Colitis Organization and the European Society for Paediatric Gastroenterology, Hepatology, and Nutrition (ECCO-ESPGHAN) indicate that children/adolescents with a moderate to severe form of Crohn’s disease (CD) should receive a more potent treatment regimen allowing to positively influence the subsequent evolution of the disease.[2] The ultimate aim of treatment is the control of all inflammation, including at the mucosal level (mucosal healing). Recent studies suggest that obtaining mucosal healing offers a unique chance for patients to stop the natural evolution and progression of the disease.[3] This may translate to a new way of treating CD (in children as well as adult patients). We suppose that a more “intensive” treatment at disease onset increases the likelihood of deep remission and thereby may improve long-term outcomes. Currently, no

---

[a] https://clinicaltrials.gov/show/NCT02852694 or
http://apps.who.int/trialsearch/Trial2.aspx?TrialID=NCT02852694

[b] Source: protocol REDUCE-RISKinCD-PIBD-TRIAL, Version 4.0 (27 June 2018).

treatment strategy trials have been conducted in paediatric inflammatory bowel disease (IBD). Experience with immunomodulators exists for more than 40 years in the treatment of IBD, and over 15 years with anti-TNF drugs. However, it is unclear which drug should be used as first-line maintenance therapy and for which patient. A treatment strategy-based clinical trial using a risk-algorithm to identify high-risk patients for progressive disease could address this question.

1.2 REDUCE-RISK TRIAL[b]

Chronic and uncontrolled intestinal inflammation results in poor outcome in patients with Crohn's disease (reduced quality of life, more resection surgery, more hospitalization, etc.). To prevent disease progression and to avoid complications current treatment efforts concentrate on intervention strategies with immunosuppressant and biologic therapy early after diagnosis.[4-6]

Data from adult CD patients indicate that the early introduction of biologics and/or combination therapy has an advantage over conventional step-up therapy, both on remission rates and on the reduction of complications on the long term.[4-6] However, it is not conceivable to treat all CD patients with biologics and/or combination therapy: 1) for economic reasons and 2) it is not appropriate to expose patients with an indolent disease course to unnecessary risks or side effects of potent therapy.

The challenge is to identify those patients who will benefit most from an early top-down strategy. No randomized controlled trial (RCT) in adult CD patients stratified patients according to a risk profile or tested a top-down approach in stratified high-risk patients. the PIBDnet consortium identified the urgent need to answer the question if a top-down approach (early anti-TNF therapy) is efficient for children/adolescents with CD at high risk for aggressive disease progression.

1.2.1 RCT

The primary objective of the REDUCE-RISK trial is to compare the effectiveness of weekly subcutaneously administered methotrexate (MTX) for maintaining relapse-free sustained steroid/enteral nutritional (EN)-free 1-year remission compared with:[c]

---

[c] Product(s) to be tested:

- Subcutaneous MTX once weekly 15mg/m2 body surface area (BSA), with a maximal dose of 25mg/week
- Oral AZA/6MP at a dose of 2.5 mg/kg once daily rounded to the nearest multiplication of 12.5mg or oral 6MP at a dose of 1.5mg/kg once daily rounded to the nearest multiplication of 12.5mg. Heterozygote patients for thiopurine-methyl transferase (TPMT) (or with low TPMT activity (6-9nmol/h/ml erythrocytes or <9nmol 6MTG/g Hb/h) will receive half the calculated dose.
- Subcutaneous adalimumab started at a dose of 160mg followed by 80mg 2 weeks later then 40mg every 2 weeks in patients over 40kg . In patients <40kg sc doses of adalimumab are as follows: induction 160mg/1,73m2 BSA (max 160mg), followed by 80mg/1,73m2 BSA (max 80mg) 2 weeks later and maintenance of 40mg/1,73m2 BSA (max 40mg) every 2 weeks

- daily oral AZA/6MP (Azathioprine/6-mercaptopurine) in low risk paediatric CD
- subcutaneously administered adalimumab in high risk paediatric CD

The primary outcome is the rate of sustained steroid/exclusive enteral nutritional (EEN)-free remission at Month 12, where sustained remission is defined as wPCDAI (weighted Paediatric Crohn's Disease Activity Index) ≤12.5 and C reactive protein (CRP) ≤1.5 fold the normal upper limit without a relapse since week 12. Secondary outcomes contain, amongst others, the following: time to first relapse, remission at 12 weeks, protocol drug's toxicity and health-related quality of life (measured with the disease-specific IMPACT-III and generic EQ-5D questionnaires). For more details we refer to the full protocol (Version 4.0, 27 June 2018).

The patients are divided into a low- and high-risk group for aggressive disease evolution. We hypothesize that MTX is superior to AZA/6MP for maintaining remission in CD in the low-risk strata and adalimumab is superior to MTX in the high-risk strata based on real-life cohort data and the recent analysis from the RISK study.[7]

The design of this trial is a multicentre, phase IV, prospective, randomized treatment strategy with PROBE (prospective randomized open blind end-point) evaluation. Physicians completing the Paediatric Crohn's Disease Activity Index (PCDAI), weighted PCDAI (wPCDAI) and physician global assessment (PGA) must be blinded to the treatment allocation. The estimated number of patients needed for each arm in the high risk group is 68 children per arm and for each arm in the low risk group is 88 children per arm (total sample size: 312 patients). The duration of participation of each patient is 12 months.

Figure 1: the REDUCE-RISK randomised controlled trial design

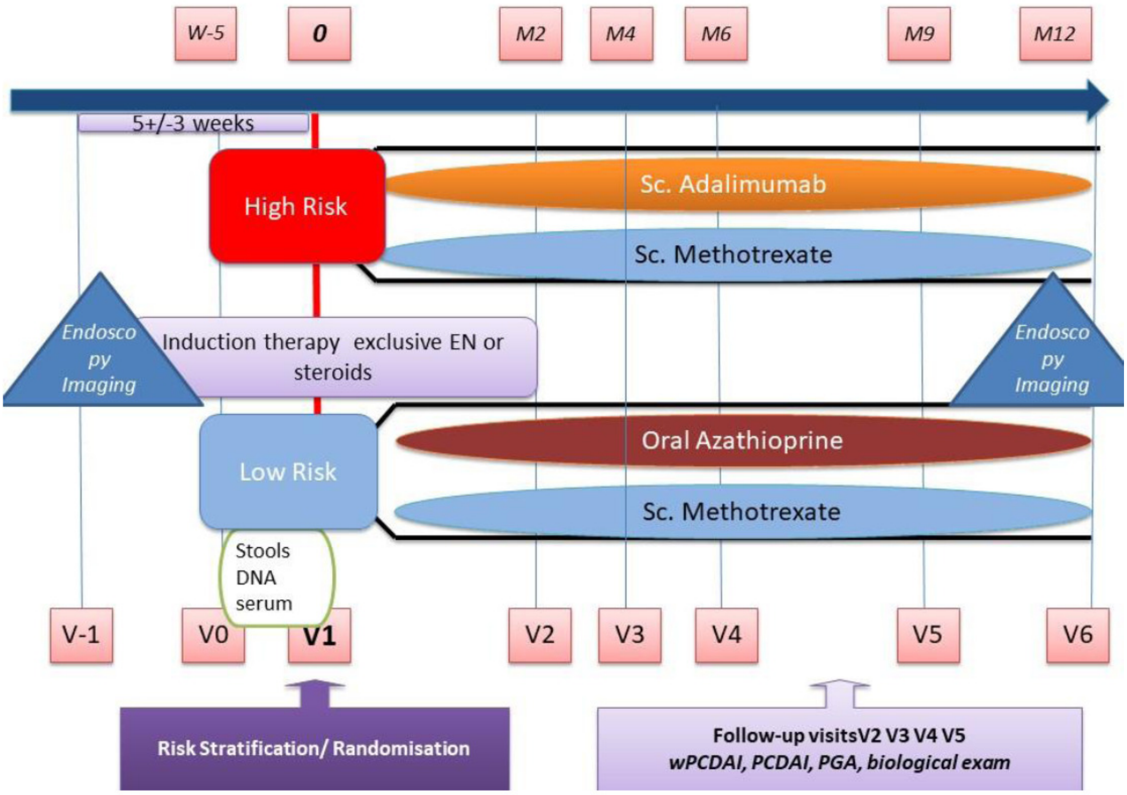

Source: protocol REDUCE-RISKinCD-PIBD-TRIAL, Version 4.0 (27 Jun 2018).
EN: enteral nutrition; M: month; (w)PCDAI: (weighted) Paediatric Crohn's Disease Activity Index; PGA: physician global assessment; Sc.: subcutaneous; V: visit; w: week.

The inclusion criteria mentioned in the protocol are as follows:

- Children 6-17, with a new-onset CD diagnosed <6 months using established criteria,[8, 9] requiring a steroid-based or EN based induction therapy
- At initial diagnosis, wPCDAI >40 or CRP>2 times upper limit at diagnosis
- all wPCDAI scores (0-120) are possible at inclusion (patients in remission and patients with active disease)
- Luminal active CD (B1) with or without B2 and/or B3 disease behaviour
- Initial exposure to 5-ASA and derivate is tolerated
- Exposure to antibiotics is tolerated
- If one of the following criteria is present, patients are allocated to the high risk group prior randomization:
    - Complex fistulizing perianal disease
    - Panenteric disease phenotype (defined as L3 with L4b per Paris classification or L3 with deep ulcers in duodenum, stomach or oesophagus (not HP- or NSAID-related))
    - Severe growth impairment (height z-score <-2 or crossing 2 percentiles or more) likely related to CD
    - Significant hypoalbuminemia (<30g/l), elevated C reactive protein (CRP) (at least 2 times above normal range), or wPCDAI >12.5 despite 3 weeks of optimized induction therapy with steroids or EEN
    - B2, B3 or B2B3 disease behaviour
    - Overall cumulative disease extend of ≥60 cm
- Informed and signed consent.

We refer to the full protocol for details of the exclusion criteria (protocol REDUCE-RISK in CD-PIBD-TRIAL, Version 4.0, 27 June 2018).

1.2.2 INCEPTION COHORT AND SAFETY REGISTRY

The incidence of paediatric-onset inflammatory bowel diseases (PIBD) has risen dramatically in recent decades. Compared to adult forms, PIBD reflects a more severe disease. Paediatric patients more often require aggressive treatment with immuno-modulators. Thereby children are exposed to a life-long risk of both serious disease and treatment-related adverse events, such as infections and malignancies. In addition, the risk profile for severe adverse events might differ between children/adolescents and adults with IBD. Therefore, there is an urgent need to generate a prospective large long term real-world inception cohort designed to analyse effectiveness and safety signals and correlate them to individual risk factors in well phenotyped patients. However, many side effects and complications are rare. To also identify and study these rare complications, there is a requirement to establish a European wide paediatric IBD safety registry in addition to the inception cohort.

The primary objective of the PIBD-NET inception cohort is to search for factors predictive for outcome, specific serious adverse events (SAEs) and for predictors of response or non-response to therapy. The secondary objective is the identification of rare complications of disease or treatment in PIBD.
The study design consists of two parts:

- An observational registry including a subcohort of patients in which biological specimen will be collected will be set up and collection of safety signalling on a wide scale will be performed.

  A robust and highly secured prospective multicenter long-term database tool (PIBD-cloud) for PIBD will be created in collaboration with a highly experienced IT small/medium-size enterprise (SME). Newly diagnosed patients will be identified and carefully phenotyped. Patients will be closely monitored for disease progression during preferably twenty years of follow up.

- A pan-European safety registry of rare complications of drugs and the disease will be created.

The generation of a pan-European prospective large long term real-world inception cohort in a registry will be designed to analyse effectiveness and safety signals and to correlate them to individual risk factors. The inception cohort in combination with a pan-European safety registry will enable us to estimate both incidence and prevalence of severe and rare complications of the disease. Moreover, the causes of these complications will be examined with the long-term aim of both predicting and reducing them in the future.

The inception cohort will include a total of 1000 children (age 0-18 years), with new-onset IBD. Per year 200 CD patients and 100 ulcerative colitis (UC) patients will be included during a 3 year period. Moreover, within these three years, 150 children (age 0-17 years), with new-onset IBD will be included for collection of biological specimens in specific centres with the capacity to perform these immunological techniques (amongst whom the Erasmus MC).

In the safety registry, patients with rare complications or side effects will be recruited via the European networks of paediatric gastroenterologists (PIBD-NET (Pediatric inflammatory bowel diseases network – www.pibd-net.org) and PEDDCReN (Paediatric European Digestive Diseases Clinical Research Network – www.peddcren.qmul.ac.uk)). Paediatric gastroenterologist connected to the networks will be invited to report new cases of a defined list of rare and serious complications seen in patients with inflammatory bowel disease.

1.3 IMPORTANCE OF ECONOMIC CONSIDERATIONS

A benefit-risk assessment is performed for the registration of pharmaceutical products. This does not necessarily include a comparative effectiveness assessment comparing the product with best available alternatives. Costs are also not taken into account when deciding about a product's registration. This is all the more important when making reimbursement decisions and is considered in so-called health technology assessments (HTA).

> HTA is "*a multidisciplinary process that summarises information about the medical, social, economic and ethical issues related to the use of a health technology in a systematic, transparent, unbiased, robust manner. Its aim is to inform the formulation of safe, effective, health policies that are patient focused*

> *and seek to achieve best value. Despite its policy goals, HTA must always be firmly rooted in research and the scientific method.*"(www.eunethta.eu)

HTA aims to provide support to decision makers in taking good decisions to keep the health care system accessible, of the highest quality as possible and durable. In HTA, an economic evaluation is performed to see whether an intervention offers value for money in comparison to other alternatives. This economic consideration supports the efficient use of limited resources. In theory, economic evaluations "*tend to guide decision makers towards the maximisation of health gains within a resource constraint, regardless of which individuals or population groups may benefit from a health intervention or perhaps be penalised by that intervention*".[10] Economic evaluations do not just consider costs. The safety and efficacy of intervention is of utmost importance and is taken into account in an economic evaluation. The link with the medical aspect is clear from the general definition of an economic evaluation: "*the comparative analysis of alternative courses of action in terms of both their costs and consequences*".[11] In economic evaluations, the incremental cost-effectiveness ratio (ICER) is calculated applying the following general formula:

- $ICER = IC/IE = (C_{Int} - C_{Comp}) / (E_{Int} - E_{Comp})$

  With C: costs; Comp: comparator; E: effects; IC: incremental cost; IE: incremental effect; Int: intervention.

The focus should be on the incremental elements, i.e. those that differ between the compared alternatives. In preparation of a future economic evaluation, we try to find out which are these most important incremental elements. The ISPOR (International Society for Pharmacoeconomics and Outcomes Research) guidelines state that "*assessing relative value is rarely the primary purpose of an experimental study. Nevertheless, when the decision is made to conduct an economic evaluation alongside a clinical trial, it is important that the economic investigator contributes to the design of the study to ensure that the trial will provide the data necessary for a high-quality economic evaluation*".[12]

A systematic review of the economic literature is performed by a health economist. The purpose is to get more useful insights and knowledge from previous economic studies.[13] The previous economic evaluations guide us in finding the key variables which enables us to provide well-directed input for the research protocol. Gathering the right information will support us at the end of the trial to make a high-quality economic evaluation.

> "*Embarking on research without reviewing systematically what is already known, particularly when the research involves people or animals, is unethical, unscientific, and wasteful.*"[14]

In chapter 2, the review of the economic literature is transparently presented. In chapter 3, based on the results of this review and from a health economist's point of view, the input for the research protocol is described.

2 ECONOMIC LITERATURE REVIEW

In this chapter, we provide information about the systematic literature search (part 2.1), the identified relevant economic evaluations (part 2.2) and a summary of findings (part 2.3). In part 2.4, we conclude with a brief discussion.

2.1 LITERATURE SEARCH

2.1.1 SEARCH STRATEGY

A systematic search for economic literature about the cost-effectiveness of adalimumab for the treatment of inflammatory bowel disease (IBD) was performed by consulting various databases. First of all, reviews on this topic were searched by consulting the Centre for Reviews and Dissemination (CRD) Health Technology Assessment (HTA) database and websites of HTA institutes mentioned on the International Network of Agencies for Health Technology Assessment (INAHTA) website. Websites of ex- or non-member HTA institutes such as the National Institute for Health and Care Excellence (NICE) were also consulted.

CRD's National Health Service Economic Evaluation Database (NHS EED), Medline (OVID), and EMBASE databases were searched to retrieve both full economic evaluations and reviews of full economic evaluations of adalimumab for IBD treatment. No language restrictions were imposed.

The search strategy started in December 2015 when first a quick and dirty search was performed to prepare the first kick-off meeting of the Horizon 2020 project in Paris (14-15 January 2016). In February 2016 HTA reports were identified on websites of HTA institutes and by consulting CRD's HTA database. In September 2016, CRD's databases, Medline (OVID), and EMBASE were searched. An overview of this search strategy and results is provided in Appendix 1 The search strategy and results provided input for the trial protocol and was discussed during the second meeting in Paris (15-16 December 2016).

2.1.2 SELECTION CRITERIA

All retrieved references were assessed against pre-defined selection criteria, in terms of population, intervention, comparator, and design (Table 1). A first 'quick and dirty' search suggested no studies with adalimumab were available in the paediatric population. Therefore, since the goal of this literature review was to provide input for the research protocol, applied selection criteria were not too restrictive. For example, the population included both adults and children and no restrictions were applied for the comparator. Adalimumab needed to be one of the compared interventions. Studies comparing other treatments and switching to adalimumab in case of no response were not included (i.e.

adalimumab is not one of the compared treatment strategies).[d] Studies comparing adalimumab and other treatments after no response to previous treatments were eligible.

The design was restricted to full economic evaluations, i.e. studies comparing at least two alternative treatments in terms of costs and outcomes. Cost-minimization, cost-effectiveness, and cost-utility analyses were eligible. Cost analysis or cost-of-illness studies were excluded, as well as studies expressing outcomes in disease-specific outcomes (e.g. cost per remission,[16, 17] cost per responder,[18] or cost per mucosal healing[19]). 'Before-after' analyses[20] comparing the costs before and after the start of treatment with adalimumab were also excluded. Studies only presented as an abstract were not considered due to a lack of sufficient details to allow a proper evaluation. English, French, German and Dutch articles were eligible.

Table 1: Economic evaluation selection criteria

	**Inclusion criteria**	**Exclusion criteria**
Population	Inflammatory bowel diseases (Crohn's disease (CD) and ulcerative colitis (UC)). Both adults and children.	Others
Intervention	Adalimumab (Humira®)	Others
Comparator	No restrictions	/
Design	Full economic evaluations expressing outcomes in life-years gained or QALYs gained	Others (e.g. cost analysis)

QALY: quality-adjusted life year

The selection of relevant articles was performed in a two-step procedure: initial assessment of the title, abstract, and keywords, followed by a full-text assessment of the selected references. When no abstract was available and the citation was unclear or ambiguous, consideration of the citation was directly made on the basis of a full-text assessment. Reference lists of the selected studies were checked for additional relevant citations. The procedure was performed by a health economist (MN) and in case of doubt for medical reasons, a medical specialist (GV) provided support.

The primary full economic evaluations were summarized in an in-house data extraction sheet (see Appendix 2). This in-house document is used as a reporting checklist to gather all relevant information. The data extraction sheets of all identified studies are working documents that provide the basis for the summary tables. These tables and a description of input variables and values used in the identified economic evaluations are presented in part 2.3. Finally, a critical assessment and discussion is presented in part 2.4.

2.2 RESULTS OF THE ECONOMIC SEARCH STRATEGY

Figure 2 presents the flow chart of the selection process. Twelve articles were identified in electronic databases. Four additional references were identified through searching websites

[d] For example, Xie et al.[15] make an analysis of initial and maintenance therapy with infliximab and then switch to adalimumab if there is no response to the initial therapy or response is lost during maintenance therapy.

of HTA institutes. The list of the 16 selected economic evaluations is provided in Table 2. Finally, due to an overlap between published full HTA reports and journal articles, 12 primary studies will be discussed in part 2.3.

Figure 2: Selection of relevant articles

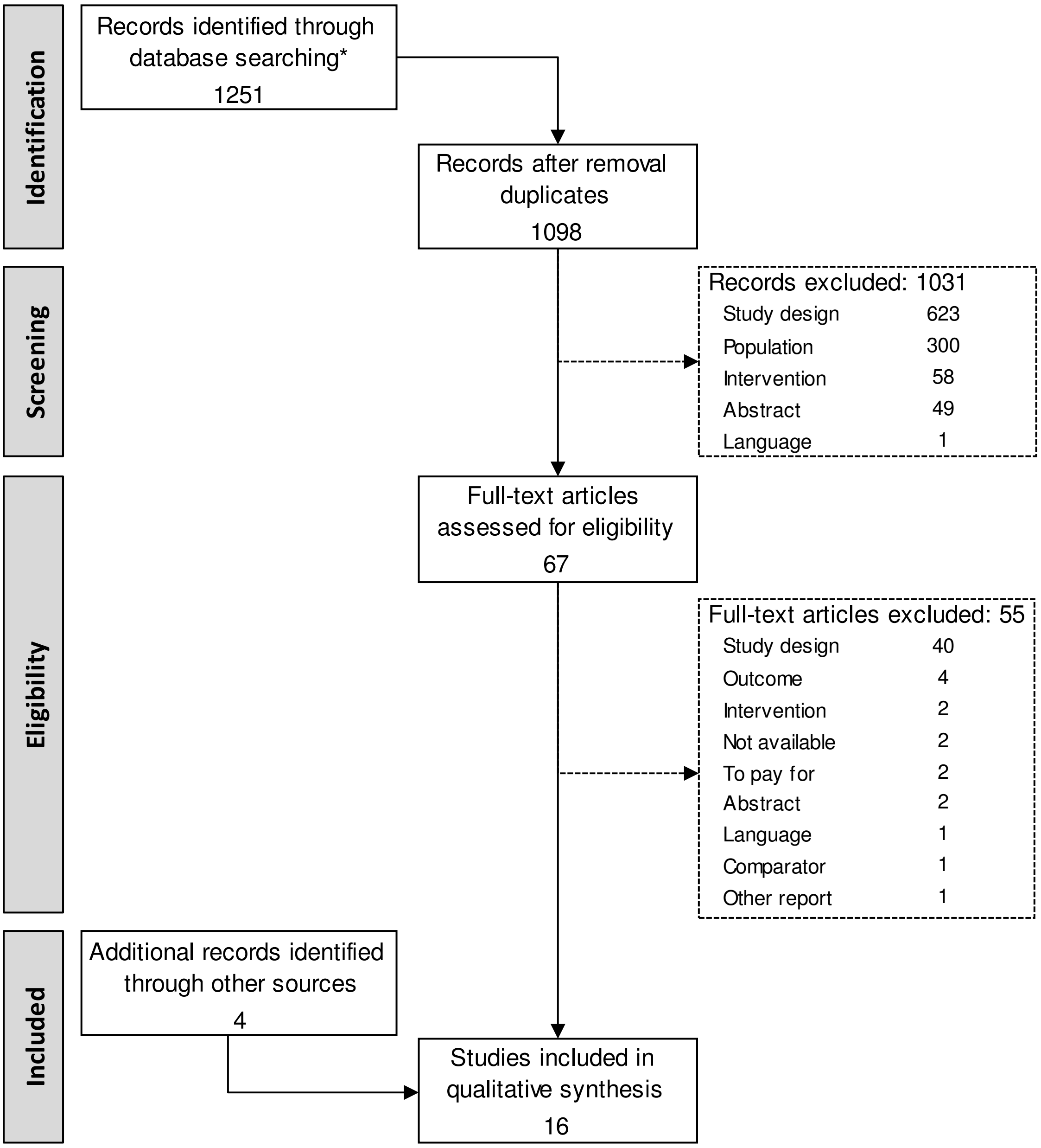

** Databases searched: Centre for Reviews and Dissemination (CRD) databases (NHS Economic Evaluation Database (NHS EED) and Health Technology Assessments (HTA)), Medline (OVID), and Embase.*

Table 2: List of selected economic evaluations

**HTA reports**
1) Archer R, Tappenden P, Ren S, Martyn-St James M, Harvey R, Basarir H, et al. Infliximab, adalimumab and golimumab for treating moderately to severely active ulcerative colitis after the failure of conventional therapy (including a review of TA140 and TA262): clinical effectiveness systematic review and economic model. Health Technol Assess 2016;20(39).[21] (study 1/16*)<br>• AbbVie. Adalimumab, golimumab and infliximab, for the treatment of ulcerative colitis (subacute). Submission to NICE; 2014. → reported on page 149-163 in the report of Archer et al.[21]<br>• MSD. Manufacturer submission of evidence: infliximab (Remicade). Submission to NICE; 2014 and MSD. Manufacturer submission of evidence: golimumab (Simponi). Submission to NICE; 2014. → reported on page 130-149 in the report of Archer et al.[21]<br>• Tappenden P, Ren S, Archer R, Harvey R, James MM, Basarir H, et al. A model-based economic evaluation of biologic and non-Biologic options for the treatment of adults with moderately-to-severely active ulcerative colitis after the failure of conventional therapy. Pharmacoeconomics. 2016 Oct;34(10):1023-38.[22] (study 2/16)<br>*Remark: this journal article is based on the full HTA report of Archer et al.[21] and is therefore not included separately in our overview.*
2) Assasi N, Blackhouse G, Xie F, Gaebel K, Marshall J, Irvine EJ, Giacomini M, Robertson D, Campbell K, Hopkins R, Goeree R. Anti-TNF-α drugs for refractory inflammatory bowel disease: Clinical- and cost-effectiveness analyses [Technology report number 120]. Ottawa: Canadian Agency for Drugs and Technologies in Health; 2009.[23] (study 3/16)<br>• Blackhouse G, Assasi N, Xie F, Marshall J, Irvine EJ, Gaebel K, et al. Canadian cost-utility analysis of initiation and maintenance treatment with anti-TNF-alpha drugs for refractory Crohn's disease. Journal of Crohn's and Colitis. 2012 21 Jul 2012;6(1):77-85.[24](study 4/16)<br>*Remark: this journal article is based on the full HTA report of Assasi et al.[23] and is therefore not included separately in our overview.*
3) Canadian Agency for Drugs and Technologies in Health (CADTH). Common drug review pharmacoeconomic review report for Simponi. November 2014.[25] (study 5/16)

4) Dretzke J, Edlin R, Round J, Connock M, Hulme C, Czeczot J, et al. A systematic review and economic evaluation of the use of tumour necrosis factor-alpha (TNF-α) inhibitors, adalimumab and infliximab, for Crohn's disease. Health Technol Assess 2011;15(6).[26] (study 6/16)<br>• Critique of the submission on adalimumab by Abbott. → reported on page 109-120 in the report of Dretzke et al.[26]<br>• National Institute for Health and Care Excellence (NICE). Infliximab and adalimumab for the treatment of Crohn's disease's (TA187); May 2010.[27] (www.nice.org.uk/guidance/ta187/resources/infliximab-review-and-adalimumab-for-the-treatment-of-crohns-disease-82598501180869) (study 7/16)<br>*Remark: the results mentioned in the economic part of this report are based on the full HTA report of Dretzke et al.[26] and is therefore not included separately in our overview.*
5) Essat M, Tappenden P, Ren S, Bessey A, Archer R, Wong R, Hoque S, Lobo A. Vedolizumab for the treatment of adults with moderately to severely active ulcerative colitis: A Single Technology Appraisal. School of Health and Related Research (ScHARR), 2014.[28] (study 8/16)<br>• Essat M, Tappenden P, Ren S, Bessey A, Archer R, Wong R, et al. Vedolizumab for the treatment of adults with moderate-to-severe active ulcerative colitis: an evidence review group perspective of a NICE single technology appraisal. PharmacoEconomics. 2016;34(3):245-57.[29](study 9/16)<br>*Remark: this journal article is based on the full HTA report of Essat et al.[28] and is therefore not included separately in our overview.*
6) Rafia R, Scope A, Harnan S, Stevens JW, Stevenson M, Sutton A, Dickinson K, Parkes M, Mayberry J, Lobo A. Vedolizumab for the treatment of adults with moderately to severely active Crohn's disease: A Single Technology Appraisal. School of Health and Related Research (ScHARR), 2014.[30] (study 10/16)
**Journal articles**
7) Bodger K, Kikuchi T, Hughes D. Cost-effectiveness of biological therapy for Crohn's disease: Markov cohort analyses incorporating United Kingdom patient-level cost data. Alimentary Pharmacology and Therapeutics. 2009;30(3):265-74.[31] (study 11/16)
8) Kaplan GG, Hur C, Korzenik J, Sands BE. Infliximab dose escalation vs initiation of adalimumab for loss of response in Crohn's disease: a cost-effectiveness analysis. Alimentary Pharmacology and Therapeutics. 2007;26(11-12):1509-20.[32] (study 12/16)
9) Loftus EV, Johnson SJ, Yu AP, Wu EQ, Chao J, Mulani PM. Cost-effectiveness of adalimumab for the maintenance of remission in patients with Crohn's disease. European Journal of Gastroenterology and Hepatology. 2009;21(11):1302-9.[33] (study 13/16)<br>• Remark: Dretzke et al.[26] refer to this study that was funded and supported by the Abbott Laboratories. However, since this article reports somewhat different results, we also include it in our overview.

10) Stawowczyk E, Kawalec P, Pilc A. Cost-utility analysis of 1-year treatment with adalimumab/standard care and standard care alone for ulcerative colitis in Poland. European Journal of Clinical Pharmacology. 2016((Stawowczyk E.) StatSoft Polska Sp. z o.o., Krakow, Poland):1-7.[34] (study 14/16)
11) Tang DH, Armstrong EP, Lee JK. Cost-utility analysis of biologic treatments for moderate-to-severe Crohn's disease. Pharmacotherapy:The Journal of Human Pharmacology & Drug Therapy. 2012;32(6):515-26.[35] (study 15/16)
12) Yu AP, Johnson S, Wang ST, Atanasov P, Tang J, Wu E, et al. Cost utility of adalimumab versus infliximab maintenance therapies in the United States for moderately to severely active Crohn's disease. PharmacoEconomics. 2009;27(7):609-21.[36] (study 16/16)

** The 16 studies identified in our search strategy (see Figure 2) are numbered in this table (study x/16). Due to an overlap between full HTA reports and journal articles, the summary tables in part 2.3 refer to 12 primary studies.*

2.3 OVERVIEW OF ECONOMIC EVALUATIONS

2.3.1 GENERAL INFORMATION

Table 3 provides an overview with general information of the included published studies. Most studies were performed for the UK (n=6). Two studies made an analysis for Canada, another three for the US, and one for Poland. Almost all studies explicitly declared conflicts of interest. All studies are cost-utility analysis. This is related to the inclusion criteria of our selection since disease-specific outcomes were excluded. Some short-term models applied a decision tree, while most models are Markov models. In some cases, an initial decision tree is used (e.g. to determine the probabilities of induction response or remission for biological drug treatments[21, 28, 30]), while a Markov component is used to estimate long-term outcomes (e.g. for maintenance drug therapy).

Several models use a short-term horizon, reflecting the follow-up period of the underlying trials, while others extrapolate results to longer time horizons of 5, 10, 30 years or lifetime (see Table 3). The applied discount rates are in agreement with national recommendations, except for one study where the manufacturer assumed an annual discount rate of 3% for both health and cost outcomes, although the CADTH guidelines recommend a 5% discount rate.[25] However, a 5% discount was applied in a sensitivity analysis. In studies with a 1-year time horizon, costs and effects were not discounted. In these cases, if sensitivity analyses were performed with longer-term horizons, national recommended discount rates were applied.

Table 3: General information on the identified economic evaluations

Study	Country	CoI	Analytic technique	Design	Time horizon	Discount rate
**Archer et al., 2016 (21)**	UK	No	CUA	Markov	lifetime	C&E: 3.5%
AbbVie submission		Yes	CUA	Markov	10 years	C&E: 3.5%
MSD submission		Yes	CUA	Decision tree + Markov	10 years	C&E: 3.5%
**Assasi et al., 2009 (23)**	Canada	No	CUA	Markov	5 years	C&E: 5%
**Bodger et al., 2009 (31)**	UK	No	CUA	Markov	lifetime (60 years)	C&E: 3.5%
**CADTH, 2014 (25)**	Canada	Yes/No*	CUA	Markov	10 years	C&E: 3%
**Dretzke et al., 2011 (26)**	UK	No	CUA	Markov	1 year	/
Abbott submission		Yes	CUA	Markov	1 year (56 weeks)	/
**Essat et al., 2014 (28)**	UK	Yes/No**	CUA	Decision tree + Markov	10 years	C&E: 3.5%
**Kaplan et al., 2007 (32)**	US	Yes	CUA	Decision tree	1 year	/
**Loftus et al., 2009 (33)**	UK	Yes	CUA	Regression model	1 year	C&E: 3.5%
**Rafia et al., 2014 (30)**	UK	Yes/No**	CUA	Decision tree + Markov	10 years	C&E: 3.5%
**Stawowczyk et al., 2016 (34)**	Poland	No	CUA	Markov	30 years	C: 5%; E: 3.5%
**Tang et al., 2012 (35)**	US	Not declared	CUA	Decision tree	1 year (54 weeks)	/
**Yu et al., 2009 (36)**	US	Yes	CUA	Decision tree	1 year (56 weeks)	/

C: costs; CoI: conflict of interest; CUA: cost-utility analysis; E: effects.
** Submission by manufacturer reviewed by CADTH team (Common Drug Review Analyses)*
*** The manufacturer submitted a model-based health economic analysis as part of their submission, which was then evaluated by a team of researchers from ScHARR (School of Health and Related Research (ScHARR)).*

2.3.2 POPULATION AND COMPARED INTERVENTIONS

The primary economic evaluations investigated treatment strategies for adult patients with moderate to severe UC or CD (Table 4). Small differences in the description (if any) of moderate to severe disease could exist, e.g. a Crohn's Disease Activity Index (CDAI[e]) ≥200,[23] or between 220 and 450.[35] The average age is between 35-40 years.[f] Patients weigh on average 69-77kg. The studies published in 2016 mention 56% or 57% are male, while in the study of Bodger et al.[31] this is 40%. In two studies, the base-case analysis relates to an adult UC or CD population, while a secondary analysis is considered for the paediatric population.[21, 26] The authors consider this as an exploratory analysis as the efficacy data are drawn from trials undertaken within an adult UC population.[21]

One study included both an analysis for CD and UC patients.[23] However, in the latter, no separate adalimumab strategy was included and therefore not included in this overview.[g] Similarly, in the report of Essat et al.[28] and Rafia et al.[30] reference was made to a model submitted by the manufacturer for three populations. Anti-TNF-α agents (infliximab, adalimumab, golimumab and vedolizumab) were included only in the analysis of the anti-TNF-α naïve population and were excluded from the analyses of the mixed intention-to-treat[h] (ITT) and anti-TNF-α failure populations. Therefore, only the analysis of patients who are anti-TNF-α naïve were considered relevant for this review.

As indicated in Table 4, most studies explicitly mention patients failed[i] to respond to standard therapy before adalimumab is considered. In all but three studies[32, 35, 36] and the MSD submission,[21] conventional non-biological therapy is considered as a comparator. This usually exist of a mix of 5-aminosalicylates (5-ASAs), corticosteroids and immunosuppressants. In two studies, only biologicals are included.[32, 36] The study of Kaplan et al.[32] considered whether dose escalation of infliximab (to 10 mg/kg every 8

---

[e] The CDAI measures the disease severity. It uses a recall period of 7 days. "*Variables measured include number of liquid stools, abdominal pain, general well-being, extraintestinal complications, use of antidiarrhoeal drugs, abdominal mass, haematocrit and body weight. Scores range from 0 to approximately 600, with higher scores corresponding to more severe disease. … Values of below 150 are suggestive of quiescent disease (remission) and values above 450 are associated with very severe disease.[37] Severe disease is thought to be above 300. Some investigators, however, have arbitrarily labelled CDAI scores of 150–219 as mildly active disease and scores of 220–450 as moderately active disease.[38]*"[26]

[f] We remark that we only looked at the description of the population in the economic part of the included reports. It is possible that more detailed information was available in the medical part of the reports or in the underlying primary trials.

[g] The three compared management strategies in the UC populations were: usual care (strategy A); 5 mg/kg infliximab plus adalimumab (strategy B); and 5 mg/kg and 10 mg/kg infliximab plus adalimumab (strategy C).[23]

[h] The mixed ITT population included both patients who have previously received anti-TNF-α therapy and those who are anti-TNF-α naïve.

[i] Failure includes intolerance, inadequate response or loss of response.

weeks) is a cost-effective strategy compared with adalimumab initiation after loss of response to 5mg/kg of infliximab. Also the study of Yu et al.[36] compares infliximab and adalimumab. This study was also part of the Abbott submission, which contained two models: one comparing the cost-effectiveness of adalimumab as a maintenance therapy against standard care (SC) and one comparing the cost-effectiveness of adalimumab and infliximab as maintenance therapies.[26] The report of Dretzke et al.[26] which made a critical assessment of Abbott's submission argued that "*the latter model will be relevant only where both adalimumab and infliximab have been first justified as maintenance therapies versus standard care (SC). Where one or both maintenance therapies are not cost-effective versus SC, this comparison provides no information to decision-makers.*"[26] Therefore, Dretzke et al.[26] concentrate on the model including standard care as a treatment option. For details of the other model, we refer to the study of Yu et al.[36]

In two studies[21, 28] and the MSD submission,[21] surgery (colectomy) is taken into account as an initial treatment option. In these studies, surgery is included both as one of the alternative treatment strategies as well as a downstream component of the pathway for patients in the other treatment strategies. In other models, like the models discussed in the CADTH report[25] and from the AbbVie submission,[21] surgery is not considered a direct comparator but only included as a treatment for patients who failed both biological and non-biological drug treatments.

Next to adalimumab, the most frequently included biological treatments are infliximab, golimumab and vedolizumab. Certolizumab pegol and natalizumab are also included in individual studies (see Table 4). Treatment schedules for these biologicals are as follows:

- Adalimumab: induction: 160mg (week 0), 80mg (week 2); maintenance: 40mg every other week (starting from week 4).[21, 23, 25, 28, 32, 34-36]
  OR induction: 80mg[j] (week 0), 40mg (week 2); maintenance: 40mg every other week.[26, 30, 31, 33]
- Infliximab: induction: 5mg/kg (week 0, 2 and 6); maintenance: 5mg/kg every 8 weeks. [21, 23, 25, 28, 31, 35, 36]
  OR induction: 5mg/kg (week 0 and 2); maintenance: 5mg/kg every 8 weeks.[30]
- Golimumab: induction: 200mg (week 0), 100mg (week 2); maintenance: 50mg every 4 weeks (body weight <80kg) or 100mg every 4 weeks (body weight ≥80kg).[21, 25]
- Vedolizumab: induction: 300mg (week 0 and 2); maintenance: 300mg every 8 weeks.[28, 30]

---

[j] Rafia et al mention that "*the recommended Humira induction dose regimen for adult patients with moderately to severely active Crohn's disease is 80 mg at week 0 followed by 40 mg at week 2. In case there is a need for a more rapid response to therapy, the regimen 160 mg at week 0 (dose can be administered as four injections in one day or as two injections per day for two consecutive days), 80 mg at week 2, can be used with the awareness that the risk for adverse events is higher during induction.*"[30]

- Certolizumab pegol: induction: 400mg (week 0, 2 and 4); maintenance: 400mg every 4 weeks.[35]
- Natalizumab: 300mg every 4 weeks.[35]

Some differences in doses/dose escalation or duration exist between the economic evaluations. Concerning golimumab, the report of Essat et al.[28] mentions the manufacturer's model did not include the 100mg dose for patients with body mass >80kg. As mentioned above, the model of Kaplan et al.[32] includes a dose escalation for infliximab (10mg/kg). For adalimumab, based on data reported in the AbbVie submission, Archer et al. include a dose escalation in maintenance treatment in 27% of cases from a dose of 40mg every other week to 40mg weekly.[21] In the MSD submission, it is stated that "*22.9% patients in the ULTRA2 trial require dose escalation but also states that experts advising on the submission suggested that the actual proportion of patients in clinical practice may be as high as 80%. The manufacturer argues that the assumption that 50% patients dose escalate is conservative.*"[21] The duration of treatment might also be different but is not always clearly stated. Bodger et al.[31] include 1 or 2 years of treatment with adalimumab or infliximab after which patients return to standard care.

Finally, the report of Dretzke et al. includes, next to standard care, both an induction and a maintenance therapy option for adalimumab. The maintenance therapy is as described above. However, their induction therapy only involves a loading dose of 80mg at week 0 and 40mg at week 2, with no further treatment.[26]

Table 4: Population and compared interventions in the identified economic evaluations

Study	Population	Failure	Age*	Weight*	Sex	Interventions							
						ADA	IFX	GOL	VED	CER	NAT	Conv.	Surg.
**Archer et al., 2016 (21)**	Patients with moderate to severe UC who have failed at least one prior therapy.<br>- base-case analysis: adult UC population;<br>- secondary analysis: paediatric population	Y	40y	77kg	57%	x	x	x				x	x
AbbVie submission	Moderate to severe UC<br>- base case: previously exposed to anti-TNF-α therapy (excl. ADA);<br>- secondary analysis: naive to anti-TNF-α agents.	Y		75kg		x						x	
MSD submission	Moderate to severe UC who have failed previous drug treatment.	Y	40y		56%	x	x	x					x
**Assasi et al., 2009 (23)**	adult patients with CD who were refractory to conventional non-anti-TNF therapy with a CDAI ≥200.	Y	37y	73kg		x	x					x	
**Bodger et al., 2009 (31)**	Moderate to severely active CD.		35-40y		40%	x	x					x	
**CADTH, 2014 (25)**	Moderately to severely active UC (Mayo score of 6 to 12 and endoscopic subscore ≥2) with inadequate response to or failed to tolerate pharmacotherapies or demonstrated corticosteroid dependence.	Y				x	x	x				x	
**Dretzke et al., 2011 (26)**	Moderate-to-severe CD patients who were resistant to standard therapy.<br>- Scenario analysis for children.	Y				x	x					x	
Abbott submission	Moderate and severe CD.					x	(x)**					x	
**Essat et al., 2014 (28)**	Moderately to severely active UC.	Y				x	x	x	x			x	x
**Kaplan et al., 2007 (32)**	Moderate to severely active CD who achieved remission following induction by 5 mg/kg of infliximab at weeks 0, 2 and 6, but lost their response during maintenance therapy dosed every 8 weeks.	Y	35y			x	x						
**Loftus et al., 2009 (33)**	Maintenance treatment of CD.					x						x	
**Rafia et al., 2014 (30)**	Moderately to severely active CD.	Y		69kg		x	x		x			x	
**Stawowczyk et al., 2016 (34)**	Moderate to severe UC despite concurrent therapy with steroids and/or azathioprine or 6-mercaptopurine.	Y	39.6y	75.37kg	57.3%	x						x	
**Tang et al., 2012 (35)**	Moderate-to-severe CD (CDAI score of 220–450) that failed to respond to standard therapy and who were treatment naive to biologics.	Y	35y	70kg		x	x			x	x		
**Yu et al., 2009 (36)**	Moderately to severely active CD.			70kg		x	x						

** Age and weight is only included in this table if explicitly mentioned in the journal article or economic part of the HTA report.*
*** for details of the comparison between ADA and IFX: see Yu et al.*
ADA: adalimumab; CD: Crohn's disease; CDAI: Crohn's disease activity index; CER: certolizumab pegol; Conv.: conventional non-biologic therapies; eow: every other week; GOL: golimumab; IFX: infliximab; NAT: natalizumab; Surg.: surgery; UC: ulcerative colitis; VED: vedolizumab; w: week.

2.3.3 COSTS

Most economic evaluations are performed from the *perspective* of the healthcare payer (Table 5). In the case of Tang et al.,[35] this was a managed care organization in the US. As a consequence, this study excluded patient co-payments for biologic prescriptions of infliximab and natalizumab and costs due to co-payments of self-injectable biologic products (adalimumab and certolizuamb pegol). Two studies also include a scenario including costs related to lost productivity. Loftus et al.[33] assume that each CD-related hospitalization corresponds to a missed interval of work equal to the average duration of serious adverse events leading to hospitalization (on average 16.55 days based on the CHARM trial[39]). This was then multiplied with an 8-h workday and an average hourly wage in the UK of £13.00.[33] Stawowczyk et al.[34] included indirect costs based on an unpublished study carried out in Poland on 202 patients with UC. Indirect costs included absenteeism, presenteeism, and leaving earlier the labour market. Yearly indirect costs for remitted patients counted to PLN6523.75 (~€1553[k]). For patients with active disease, this was PLN22 934.58 (~€5461).

Table 5: Study perspective, currency and year of costing in the identified economic evaluations

Study	Perspective	currency and year of costing
**Archer et al., 2016 (21)**	Payer's perspective (NHS and PSS)	£ (2013-2014)
AbbVie submission	UK NHS	£ (2013-2014)
MSD submission	UK NHS	£ (NA)
**Assasi et al., 2009 (23)**	Publicly funded health care system	CAD (2008)
**Bodger et al., 2009 (31)**	UK NHS	£ (2006-2007)
**CADTH, 2014 (25)**	Public payer perspective	CAD (2013)
**Dretzke et al., 2011 (26)**	Payer's perspective (NHS and PSS)	£ (2006)
Abbott submission	Payer's perspective (NHS and PSS)	£ (2006)
**Essat et al., 2014 (28)**	UK NHS	£ (2011-2012)
**Kaplan et al., 2007 (32)**	Not explicitly mentioned - Payer's perspective	$ (2006)
**Loftus et al., 2009 (33)**	UK NHS	£ (2006)
**Rafia et al., 2014 (30)**	UK NHS	£ (2012-2013)
**Stawowczyk et al., 2016 (34)**	Public payer	PLN (2015)
**Tang et al., 2012 (35)**	Payer's perspective (managed care organization)	$ (2010)
**Yu et al., 2009 (36)**	Private payer perspective	$ (2006-2007)

CAD: Canadian dollars; NA: not available; NHS: National Health Service; PLN: Polish zloty; PSS: Personal Social Services.

Biological treatments

An overview of the *unit costs of biological treatments* is provided in Table 6. It is remarkable that while the unit cost for 40mg of adalimumab is lower than 100mg of infliximab in all the studies for the UK and Canada, this is the opposite in all US studies.

---

[k] The authors mention that €1=4.2PLN, based on the average exchange course from the year 2015.[34]

Infliximab is assumed to be administered in a day-case setting, while adalimumab and golimumab can be self-administered subcutaneously. As a result, extra drug administration costs are taken into account for infliximab treatment, while all except one of the analyses assume no administration costs for adalimumab. No costs are included for training patients to self-inject the pre-filled pen or pre-filled syringe.[21] Tang et al.[35] indicate not everybody might be able to self-inject adalimumab, which is assumed to be the case in 5.5% of patients (see Table 7). While unit costs are lower for infliximab in comparison with adalimumab in the US studies, *total treatment costs* with infliximab are not always lower if administration costs are also taken into account. In the study of Yu et al.,[36] total therapy cost for adalimumab equals the drug costs of $17 176 (see Table 7). For infliximab, the total therapy cost of $18 214 consists of the drug costs ($14 663) + the drug administration costs ($1605) + excess uninfused drug costs ($1946).

The UK report of Archer et al.[21] shows the importance of the percentage of dose escalation for the relative total treatment cost of adalimumab versus infliximab. In the MSD manufacturer submission, an assumed 50% of dose escalation results in higher maintenance treatment costs for adalimumab in comparison with infliximab (Table 7). In contrast, applying the same unit costs and treatment schedule for both drugs, the analysis of Archer et al.[21] has a lower average maintenance treatment cost with adalimumab in comparison with infliximab by incorporating a dose escalation for about 27% of patients (27.4%x£9187.08 + 72.6%x£4593.54 = £5852.17 versus £6444.73 (Table 7)).

While the unit costs for adalimumab and infliximab are the same, the total drug costs for these biologicals during the induction cycle are higher in the UK study from Essat et al.[28] in comparison with Rafia et al.[30] (see Table 7). This is due to the higher start-up dose in the first study (see above in part 2.3.2).

For some studies, no disaggregated information on treatment costs was provided and could thus not be included in Table 7. For example, Bodger et al.[31] provided the mean lifetime discounted cost per treatment arm, and Dretzke et al.[26] provided total costs by health state (remission, relapse, surgery or post-surgery remission).

Next to adalimumab, infliximab and golimumab, three additional biological treatments were analysed in two other studies. The total drug treatment cost with vedolizumab in the study of Essat et al.[28] and Rafia et al.[30] was kept confidential. Next to adalimumab and infliximab, the US study of Tang et al.[35] also considered treatment with certolizumab pegol and natalizumab. The total drug cost of $24 830 and $39 101, respectively, was higher in comparison with drug costs for adalimumab ($22 750) or infliximab ($20 607).

Standard care

Costs for standard care are substantially lower in comparison with biological treatment costs. In Archer et al.[21] standard care in the induction phase of 8 weeks, consisting of 5-ASAs, AZA (azathioprine), 6-MP (6-mercaptopurine) and prednisolone, costs £167.6. In the maintenance phase of 26 weeks, this is £343.8. In their report they refer to a total weighted conventional therapy cost per 2-week cycle of £18.6 in the AbbVie submission.[21] In the MSD submission,[21] patients in the standard non-biological treatment group are assumed to receive mesalazine, AZA, 6-MP, ciprofloxacin and prednisolone. The same use of background therapies is assumed for all biological treatment arms. In the colectomy group, patients are assumed to undergo immediate colectomy and the actual drug acquisition cost

for this group is zero within their model. Standard care cost differences were small between the different treatment strategies. For example, in their model comparing golimumab with adalimumab, infliximab or standard care, the background therapy costs were £251.43 per cycle for the standard non-biological treatment group, versus £200.03 per cycle for the biological treatments during the induction treatment (cycle = 8 weeks). During maintenance treatment (cycle = 2 months), this was £121.15 versus £120.98, respectively.[21]

Assasi et al.[23] include a total non anti-TNF outpatient drug costs per cycle (8 weeks) of CAD116.30 for drug responsive patients and CAD85.95 for drug refractory patients. In the report of Essat et al.,[28] conventional treatment (balsalazide, mesalazine, olsalazine, sulfasalazine and budesonide, prednisolone, azathioprine, 6-MP and methotrexate) costs £153.6 per induction cycle (6 weeks) and £204.8 per maintenance cycle (8 weeks). The authors assume that whilst patients are receiving biologic therapy, the costs of conventional therapies are halved (£102.4). The same logic is applied in the study of Rafia et al.[30] with a cost of £52.62 per induction cycle and £70.16 per maintenance cycle, which is halved (£35.08) for patients whilst receiving biologic treatment. In the Polish study, standard treatment costs per cycle (8 weeks) are PLN204.32 (~€48.6).[34] In other models, the costs for standard care could not always be separated since e.g. aggregated type-specific health-state costs (for remission, relapse, surgery, post-surgery) are provided.[26]

Colectomy/surgery

From the three studies including colectomy as an alternative treatment strategy, Archer and Essat refer to information from the study of Buchanan et al.[40] to include a cost of £13 452[21] and £13 577[28] for surgery. In the MSD submission, the cost for colectomy is £8968.

Other studies include surgery as an event in their model, without providing further details on the type of hospitalisation. A wide range of costs is mentioned from a surgery cost of PLN12 480 (~€2971) in the Polish study of Stawowczyk et al.[34] to a hospital unit costs of $31 923 in the US study of Yu et al.[36] The cost of surgery was $11 341 in the US study of Kaplan et al.,[32] £10 581 in the UK study of Rafia et al.,[30] and CAD19 269 in the CADTH study.[25]

Costs related to complications after surgery are mentioned separately in several studies: PLN4160 (~€990);[34] late complications (postcolectomy): £2542.64;[21] early complications (intra-abdominal sepsis: CAD22 082; wound infection: CAD3937; small bowel obstruction: CAD6399) and late complications (pouchitis: CAD191.64; small bowel obstruction: CAD6399; anal fistula: CAD9795).[25]

Table 6: Unit costs of biological treatments

Study	Country	Biological drug unit costs			
		**ADA**	**IFX**	**GOL**	**Others**
**Archer et al., 2016 (21)**	UK	40mg: £352.14	100mg: £419.62	50mg: £762.97<br>100mg: £1525.94	
AbbVie submission		40mg: £352.14	-	-	
MSD submission		NA	NA	NA	
**Assasi et al., 2009 (23)**	Canada	40mg: CAD772.42*	100mg: CAD1027.80*	-	
**Bodger et al., 2009 (31)**	UK	NA	NA	-	
**CADTH, 2014 (25)**	Canada	40mg: CAD740.36	100mg: CAD968.20	50mg: CAD1490.41	
**Dretzke et al., 2011 (26)**	UK	40mg: £357.50	-	-	
Abbott submission		40mg: £357.50	-	-	
**Essat et al., 2014 (28)**	UK	40mg: £352.14	100mg: £419.62	50mg: £762.97	VED: 300mg: £2050**
**Kaplan et al., 2007 (32)**	US	40mg: $944	700mg: $4639<br>(100mg: about $663)	-	
**Loftus et al., 2009 (33)**	UK	40mg: £357.50	-	-	
**Rafia et al., 2014 (30)**	UK	40mg: £352.14	100mg: £419.62		VED: 300mg: £2050**
**Stawowczyk et al., 2016 (34)**	Poland	1mg: PLN54.55<br>(40mg: €519.5***)	-	-	
**Tang et al., 2012 (35)**	US	2x40mg: $1820<br>(40mg: $910)	100mg: $735.96		CER: 2x200mg: $1909.99<br>NAT: 300mg: $3554.66
**Yu et al., 2009 (36)**	US	40mg: $660.11	100mg: $580.94	-	

** Unit Cost (including 8% pharmacy markup), €1 = CAD1.5 (exchange rate 16 November 2017)*
*** In these studies,*[28, 30] *the basic NHS list price of vedolizumab was £2050 per 300mg vial. The manufacturer's model included a lower drug acquisition cost (Patient Access Scheme). This negotiated price was kept confidential in the report.*
**** In Stawowczyk et al.: €1 = 4.2PLN*
ADA: adalimumab; CER: Certolizumab pegol; GOL: golimumab; IFX: infliximab; NAT: Natalizumab; VED: Vedolizumab.

Table 7: Biological treatment costs

Study	Country	Biological drug treatment costs			
		**ADA**	**IFX**	**GOL**	**Others**
**Archer et al., 2016 (21)**	UK	Induction phase (8-week duration):			
		£2817.12<br>(4×40mg + 2×40mg (self-administered))	£5928.44<br>(12×100mg (3 outpatient appointments) = 5035.44 (acquisition) + 893.18 (administration))	£4577.82<br>(4×50mg + 2×50mg (self-administered))	-
		Maintenance phase (26-week duration):			
		£4593.54 (13.04×40mg: 72.6%)<br>£9187.08 (26.08×40mg: 27.4%)	£6444.73<br>(13.04×100mg of IFX (3.26 outpatient appointments)	£9952.67 (13.04×50mg: 31.6%)<br>£4976.34 (6.52×50mg: 68.4%)	-
AbbVie submission		NA	-	-	-
MSD submission		Induction phase (8-week duration):			
		£3169.26<br>(1×160mg + 1×80mg + 3×40mg)	£5497.44<br>(12×100mg over three administrations)	£3051.88<br>(2×100mg + 1×100mg)	-
		Maintenance phase (2-month cycles):			
		£2288.91<br>50% 40mg eow (4.33 doses/cycle);<br>50% 40mg ew (8.67 doses/cycle).	£1985.19<br>(5mg/kg every 8 weeks)	£1653.10<br>31.6% 100mg every 4 weeks;<br>68.4% 50mg every 4 weeks.	-
**Assasi et al., 2009 (23)**	Canada	Induction: CAD7801<br>Maintenance (per 8w-cycle): CAD3088	Induction: CAD$12,410<br>Maintenance (per 8w-cycle): CAD4111	-	-
**Bodger et al., 2009 (31)**	UK	NA	NA (Administration cost of £168 per infusion)	-	-
**CADTH, 2014 (25)**	Canada	First Cycle: CAD7404<br>Subsequent Cycle: CAD4442<br>Year 1: CAD22 210<br>Thereafter: CAD19 249	First Cycle: CAD10 892<br>Subsequent Cycle: CAD5446<br>Year 1: CAD29 046<br>Thereafter: CAD23 600	First Cycle: CAD7854<br>Subsequent Cycle: CAD4470<br>Year 1: CAD22 356<br>Thereafter: CAD19 375	-
**Dretzke et al., 2011 (26)**	UK	NA	-	-	-
Abbott submission		NA	-	-	-
**Essat et al., 2014 (28)**	UK	Induction cycle: £2817.12<br>Maintenance cycle: £1408.56	Induction cycle: £5035.44<br>Maintenance cycle: £1678.48	Induction cycle: £4577.82<br>Maintenance cycle: £1525.94	Vedolizumab: confidential
**Kaplan et al., 2007 (32)**	US	NA	NA (Cost of administrating i.v. infusion: $193)	-	-
**Loftus et al., 2009 (33)**	UK	NA	-	-	-
**Rafia et al., 2014 (30)**	UK	Induction cycle: £1760.70<br>Maintenance cycle: £1408.56	Induction cycle: £3356.96<br>Maintenance cycle: £1678.48	-	Vedolizumab: confidential
**Stawowczyk et al., 2016 (34)**	Poland	NA	-	-	-
**Tang et al., 2012 (35)**	US	Total drug cost by initial evaluation:		**Others**	
		$5460.00	$2943.84	Natalizumab: $10 663.98	Certolizumab pegol: $3819.98
		Total drug cost between initial and final evaluations:			
		$22 750.00	$20 606.88	Natalizumab: $39 101.26	Certolizumab pegol: $24 829.87
		Total administration cost by initial evaluation:			
		$946	$512.28	Natalizumab: $1218	Certolizumab pegol: $652
		Total administration cost between initial and final evaluations:			
		If no disability (94.5%): $255.36<br>If have disability (5.5%): $4875	$3585.96	Natalizumab: $4466	Certolizumab pegol:<br>If no disability (94.5%): $255.36<br>If have disability (5.5%): $4238
**Yu et al., 2009 (36)**	US	Total therapy cost: $17 176	Total therapy cost: $18 214	-	-

eow: every other week; ew: every week

2.3.4 ADVERSE EVENTS

The report of Archer et al.[21] mentions serious and severe adverse events (AEs) were not considered in the AbbVie model. The manufacturer notes that most AEs experienced by patients were non-serious and considered to be unrelated to the study drugs (based on results from the ULTRA2 trial[41]).[21] In addition, the manufacturer highlights the exclusion of these events represents a conservative assumption since "*the ULTRA2 trial reported slightly higher incidences of serious and severe AEs in the placebo arm than in the adalimumab arm of the trial; therefore, considering serious and severe AEs in the model would have increased medical costs and reduced health gains within the conventional management group*".[21]

Also the Polish study refers to the ULTRA2 trial[41] to justify that certain adverse events were not included in the model because adalimumab treatment was generally well tolerated and the overall safety profile of adalimumab was comparable with that of placebo.[34]

In the UK reports of Rafia et al.[30] and Essat et al.,[28] the company presents a table with unit costs associated with managing adverse events: serious infection (£1470), tuberculosis (£2272), lymphoma (£14 975), hypersensitivity (£3188) and injection site reactions (£1363). However, the ERG notes that the costs associated with treating tuberculosis and lymphoma are not used in the model since the incidence rate for these events is zero for all treatment options,[28] or that the impact of AEs on the incremental cost-effectiveness ratio (ICER) is minimal.[30]

Finally, Tang et al.[35] mentions they are not aware of evidence that demonstrates large differences in the proportion of adverse drug reactions across the four biologic treatments (adalimumab, infliximab, certolizumab pegol and natalizumab), and the frequency of these complications are low. Based on their clinical judgment, they concluded that adverse drug reactions should not be included in the structure of the model. Only progressive multifocal leukoencephalopathy (PML) occurrence with natalizumab was considered a rare but significant adverse event. The treatment cost for this event was between $14 544 (lower limit) and $22 725 (upper limit).

2.3.5 QUALITY OF LIFE

Literature review of Archer et al.

Archer et al.[21] performed a systematic literature search for utility values. Ten studies reported EQ-5D (EuroQol 5-Dimension Quality of Life Questionnaire) estimates for one or more health states relevant to their model (see Table 8). The authors considered the values reported by Woehl et al.[42] and Swinburn et al.[43] to be the most useful as "*they are UK based, included a fairly large number of patients (n = 180 and n = 230, respectively) and have the greatest coverage of the health states in the model*".[21]

Both studies are only published as an abstract. Swinburn et al. examined the impact of surgery on patients' QoL. 230 UC patients (including 30 post-surgery patients) and 100 age- and gender-matched controls were recruited into the study. EQ-5D utility scores, collected via an online survey, were compared across disease severity, among post-surgery patients versus non-surgery patients, and among post-surgery patient vs controls.[43] Results were

presented on a figure in the abstract, without mentioning the exact utility values. Archer et al.[21] extracted utility values from this graph:

- "*Seventy-eight patients had remission, 47 patients had mild disease, 31 patients had moderate disease and 44 patients had severe disease. The utility for patients post surgery was reported to be 0.59 (95% CI 0.55 to 0.63). For patients who had not undergone surgery, the scores for each disease severity are: remission utility = 0.91 (95% CI 0.87 to 0.95), mild disease utility = 0.80 (95% CI 0.70 to 0.85), moderate disease utility = 0.68 (95% CI 0.58 to 0.78) and severe disease utility = 0.45 (95% CI 0.35 to 0.55). Across the total UC pre-surgery population, the mean EQ-5D utility was reported to be 0.75 (95% CI 0.71 to 0.79). Similarly, for the matched controls, the mean EQ-5D utility was estimated to be 0.79 (95% CI 0.75 to 0.83). Swinburn et al.[43] report that, on average, post-surgery patients reported lower HRQoL* [health-related quality of life] *scores than non-surgery patients (p = 0.016) and matched controls (p = 0.03).*"[21]

The study of Woehl et al.[42] is also an abstract that we could not identify in PubMed or Google. Archer et al. provide the following details about this study:

- "*Woehl et al.[42] collected EQ-5D utility scores from 180 patients with active UC. Within this study population, the mean age was 55.0 years (SD 14.2) and the mean age at diagnosis was 34.1 years (SD 14.6). UC disease severity groups were categorised by SCAI-2 and were compared with patients with IPAA* [ileal pouch anal anastomosis] *and ileostomy. The mean EQ-5D score was 0.73 (SD 0.29). Mean EQ-5D utilities were reported to be 0.87 (SD 0.15) for remission, 0.76 (SD 0.18) for mild disease, and 0.41 (SD 0.34) for moderate to severe disease. Patients who had undergone IPAA reported an EQ-5D utility of 0.71 (SD 0.29) while patients with an ileostomy reported an EQ-5D score of 0.72 (SD 0.35). Therefore, the health utility scores for these surgery states were slightly below a mild disease severity. The difference between these five groups was statistically significant (p = 0.001).*"[21]

Table 8: Characteristics and findings of studies included in the systematic review of utility values

Study	ACT1 and ACT2 [44, 45]	PURSUIT [44, 45]	*Swinburn et al.[43]	Woehl et al.[42]	*Casellas [46]	*Leidl [47]	Vaizey [48]	Van der Valk[49]	Richards [50]	Kuruvilla [51]
**Study characteristics**										
Sample size	486**	464	230	180	528	232	173	982	56	59
Country	Various	Various	UK	UK	Spain	Germany (UK tariff)	UK	NL	UK	US
**Health state valuations**										
Remission (ranges of utilities reported by the company)	0.84–0.88	0.86–0.89	0.91	0.87	1.00	0.91	0.86	NR	NR	NR
Response (ranges of utilities reported by the company)	0.79–0.82	0.80	0.80	0.76	0.70	0.74	0.77	NR	NR	NR
Active UC (utilities)	NR	NR	0.55	0.41	0.55	0.63	0.66	NR	NR	NR
Post surgery (utilities)	NR	NR	0.59	0.71–0.72	NR	NR	NR	0.85***	0.85	0.90***

Remark: this table is copied from the report of Archer et al.[21] The authors also searched for utility values related to post-surgery complications, but none of the studies reported in this table included such information.
NL: The Netherlands; NR: not reported.
* Archer et al.[21] mention this are approximate estimates based on a graph reported in Swinburn et al.[43]
** Licensed arms only.
*** Same value reported for pouch and for ileostomy.

QoL in the identified economic evaluations

The studies from which the utility values for moderate to severe, mild, and remission or response/no response health states were retrieved are as follows:

- Archer et al.: Woehl et al.[42]
- AbbVie submission: Swinburn et al.[43]
- MSD submission: PURSUIT trial[52] and ACT1 trial[53]
- Assasi et al.: Gregor et al.[54]
- Bodger et al.: calculated from mid-point CDAI scores, based on the algorithm developed by Buxton et al.,[55]
- CADTH: PURSUIT trial[52, 56]
- Dretzke et al.: Gregor et al.[54]
- Abbott submission: Gregor et al.[54]
- Essat et al.: GEMINI1[57]
- Kaplan et al.: Gregor et al.[54]
- Loftus et al.: Gregor et al.[54]
- Rafia et al.: GEMINI II[58] and GEMINI III[59]
- Stawowczyk et al.: Woehl et al.[60]
- Tang et al.: Gregor et al.[54]
- Yu et al.: Gregor et al.[54]

Most economic evaluations refer to the study of Gregor et al.,[54] published in 1997, to retrieve relevant utility values. They used the Time Trade-off (TTO), Standard Gamble (SG), and Visual Analog Scale (VAS) methods in 180 consecutive patients with CD to obtain utilities. All methods yielded lower mean scores in patients with more severe disease. The utility values for remission versus chronically active, therapy resistant disease were: 0.96 versus 0.88 (TTO); 0.88 versus 0.74 (SG); 0.84 versus 0.61 (VAS).[54] One table presented mean utility scores for three hypothetical disease-severity states. The results for the SG technique were as follows: mild disease: 0.82, moderate disease: 0.73, or severe disease: 0.54.[54] For the TTO technique this was 0.96, 0.88 and 0.71, respectively. A second table presented the following mean utility scores for the SG technique at the initial visits: chronically active-therapy resistant: 0.74; chronically active-therapy responsive: 0.86; acute disease exacerbation: 0.77; remission: 0.88; overall: 0.81. With the TTO approach this was 0.88; 0.98; 0.89; 0.96 and 0.92, respectively.[54]

All but one of the studies referring to Gregor et al. mention to use the values from the SG approach. Only Dretzke et al.[26] make use of the TTO values. Assasi et al.[23] assigned the SG values for 'mild', 'moderate' and 'severe' disease to the remission, drug responsive, and drug refractory health states. Also the Abbott submission,[26] the study of Yu et al.[36] (which was also one of the authors of the Abbott submission), Kaplan et al.[32] and Tang et al.,[35] refer to the same study of Gregor et al. to use somewhat different utility values for specific health states. For example, Tang et al.[35] refer to both Gregor et al.[54] and Yu et al.[36] to apply the same utility value for non-remission, but a higher utility for the remission

health state (see Table 9). Upon their request, Yu et al. received from the investigators the average health utilities by CDAI interval, including utility values for patients in remission (CDAI <150) and non-remission (CDAI >150).[36] Similarly, although the utility scores were not reported based on the disease states specified in the model of Loftus et al., the authors were able to provide them with the means and standard errors of SG-calculated utility scores corresponding to the four CDAI states in their model.[33]

In their base-case analysis, Archer et al. preferred to selected the utility values from the abstract published in 2008 by Woehl et al.,[42] because the valuation for the surgery state (0.71 to 0.72) was more consistent with the other post-surgery valuations identified[49-51] as compared with the Swinburn et al.[43] study.[21]

Archer et al. note that the AbbVie submission could have mapped SF-6D utility estimates from the ULTRA2 trial onto the EQ-5D. However, the manufacturer preferred not to do this and to use the results reported by Swinburn et al.[43]

The two values that are mentioned for the different health states in Table 9 for the MSD submission refer to the values that were used in two separate models. These were based on EQ-5D valuations derived from the PURSUIT trial[52] for the golimumab model and the ACT1 trial[53] for the infliximab model.[21] Archer et al.[21] remark that different utilities are assumed in the MSD submission for achieving the same outcome at induction and maintenance. Also the utility value of remission after colectomy (0.60 – see Table 10) is much lower than the utility value for remission pre-colectomy (both during induction and maintenance (0.84-0.89 – see Table 9).

Bodger et al.[31] mapped mid-point CDAI scores to EQ-5D utility scores. An algorithm developed by Buxton et al.[55] was used (EQ-5D = 0.9168 - 0.0012xCDAI). This algorithm was based on multiple observations from 905 patients with moderate-to-severe CD who participated in the Efficacy of Natalizumab as Active Crohn's Therapy (ENACT-1) and Evaluation of Natalizumab as Continuous Therapy (ENACT-2) clinical trials.[61] We refer to our discussion (see part 2.4) for some critical remarks from the authors who developed this algorithm.

The CADTH report mentions the manufacturer used Mayo scores observed in the PURSUIT[52, 56] trial to estimate the baseline disease severity and change in disease severity related to treatment effect. The associated health-related quality of life was estimated using utilities for these health states using the EQ-5D visual analogue scale (VAS).[25]

Essat et al.[28] report the manufacturer derived utility scores for the pre-surgical states from the GEMINI1 EQ-5D values for each state. The same utility was assumed for moderate to severe responders and non-responders (Table 9). The same assumption was made in the company submission included in the report of Rafia et al.[30] The manufacturer obtained EQ-5D utility scores from the GEMINI II[58] and GEMINI III[59] studies.

Stawowczyk et al.[34] performed a systematic review of the literature to identify utility values. They preferred to use the values from another abstract published in 2007 by Woehl et al.[60] It reported EQ-5D utility values and was carried out on 18 573 patients from the UK. The utility values were reported for remitting disease, mild disease, and moderate to severe disease. Stawowczyk et al. assumed that the utility value for moderate to severe disease that responded to treatment was equal to the value for mildly active disease. In patients

undergoing treatment or who are in an active disease state or had complications after surgery, the utility value was assumed to be as in active moderate to severe disease.[34]

Table 9: Utility values assigned to different health states in the identified economic evaluations

Study	QoL								
	**Remission**	**Mild**	**Moderate**	**Severe**	**Mod.-Sev.**	**Very Sev.**	**Response**	**No response**	**Other**
**Archer et al., 2016 (21)**	Remission: 0.87						Response: 0.76	No response: 0.41	
AbbVie submission	Remission: 0.91	Mild: 0.80			Mod.-sev.: 0.55				
MSD submission	Remission:<br>- pre-col., ind.: 0.86; 0.84<br>- pre col., maint.: 0.89; 0.88						Response:<br>- pre-col., ind.: 0.80; 0.79<br>- pre col., maint.: 0.80; 0.82<br>- relapse man.: 0.76; 0.76	No response: 0.70; 0.70	relapse (relapse man.): 0.42; 0.42
**Assasi et al., 2009 (23)**	Remission: 0.82						Drug responsive: 0.73	Drug refractory: 0.54	
**Bodger et al., 2009 (31)**							- Full resp.: 0.83<br>- Part.resp.: 0.69	Nonresponse: 0.42	
**CADTH, 2014 (25)**	Remission:<br>- Pre-col.: 0.82				Mod.-sev. (pre-col.): 0.55		Response (pre-col.): 0.72		
**Dretzke et al., 2011 (26)**	Remission: 0.95		Mod. relapse: 0.88	Sev. relapse: 0.73					
Abbott submission	Remission: 0.859		Moderate: 0.795	Severe: 0.693		Very Severe: 0.433			
**Essat et al., 2014 (28)**	Remission: 0.86	Mild: 0.80			Mod.-sev. (resp. and non-resp.): 0.68				
**Kaplan et al., 2007 (32)**	Med. Rem.: 0.89	Mild flare: 0.77		Severe flare: 0.62					
**Loftus et al., 2009 (33)**	Remission: 0.859		Moderate: 0.795	Severe: 0.693		Very severe: 0.433			
**Rafia et al., 2014 (30)**	Remission: 0.82	Mild: 0.73			Mod.-sev.: 0.57				
**Stawowczyk et al., 2016 (34)**	Remission: 0.88						Response: 0.76		Active treatment: 0.42
**Tang et al., 2012 (35)**	Remission: 0.89							Nonremission: 0.75	
**Yu et al., 2009 (36)**	Remission: 0.859							Average non-remitted health utility: 0.754	

AE: adverse event; compl.: complication; ind.: induction; maint.: maintenance; man.: management; mod: moderate; surg. rem.: surgical remission; part.resp.: partial response; postcol.: postcolectomy; pre-col: pre-colectomy; resp.: responder; sev: severe; surg.rem.: Surgical remission.
Remark: the description for the different health states was not always clear and differences in these definitions might exist (e.g. whether clinical disease activity scores were used or endoscopic remission was targeted).

Large differences are noticed in the post-surgery remission utility values. Several studies assign a value equal[23] or similar[32, 35] to the utility for (medical) remission. In contrast, several other studies assign a much lower value for post-surgery remission. For example, in the MSD submission, the utility value for post-colectomy remission (0.60) was assumed to be equal to the utility for late complications (post-colectomy). Similar values for post-surgery remission were assumed in the study discussed in the CADTH report[25] (0.67), Essat et al.[28] (0.60), Rafia et al.[30] (0.57), and Stawowczyk et al.[34] (0.61), while remission utility values were much higher, 0.82, 0.86, 0.82 and 0.88, respectively.

As noted by Archer et al.,[21] in the AbbVie submission, the utility for surgery was assumed to be the same as for the moderate to severe health state. Utility values for the post-surgery health states without complications and with transient complications were taken from Tsai et al.[62] The utility for the chronic complication state was estimated by using a weighted value of rates and HRQoL impacts of chronic pouchitis (Arseneau et al.[63]), infertility (Hu et al.[64]) and male sexual dysfunction (Smith and Roberts[65]).

Bodger et al.[31] assumed that for each cycle in the surgical state, patients were assumed to experience 2 weeks at an equivalent state of health as nonresponders, and 6 weeks at an equivalent state of health as full responders (0.73 = 25%x0.42 (utility non-response) + 75%x0.83 (utility full respons)).

The CADTH report[25] mentions the manufacturer could not retrieve utilities associated with colectomy and post-colectomy health states from the available trial data. They refer to observational literature and previous cost-effectiveness analyses.[15] The manufacturer also assumed adverse events and discontinuations due to adverse events were associated with a disutility of 0.10. The CADTH experts could not find evidence to support this assumption.

Dretzke et al. did not find utility values for the surgery health state. In the absence of such data, it was assumed that the average utility for individuals in the major surgery state would be equivalent to the EQ-5D state 22222 with a utility weight of 0.516.[26]

Essat et al.[28] found out that the manufacturer’s model used values for the post-surgery state drawn from a previous economic evaluation by Punekar and Hawkins,[66] referring to a study abstract published by Woehl et al.[42] However, they remark that the values used by Punekar do not coincide with values from this underlying reference and that the post-surgical remission utility value of 0.60 did not reflect any of the values reported by Woehl et al.[42] Also disutilities associated with serious infection, acute hypersensitivity reactions, skin site reactions, tuberculosis and lymphoma were based on different underlying studies. Disutilities for the latter two AEs were actually not used in the manufacturer’s model as the incidence rate for these events were zero.[28] The manufacturer’s model discussed in Rafia et al. [30] also included disutilities related to adverse events (seeTable 10).

Stawowczyk et al.[34] assumed that in the post-surgery remission state, the utility value would be lower than that in the remission after the treatment state to reflect the effect of chronic complications after a colectomy on the patient’s quality of life.

Tang et al.[35] also included a utility loss for patients with progressive multifocal leukoencephalopathy (PML) after receiving natalizumab, based on the Health Utility Index III that assessed patients with multiple sclerosis and PML.[67]

Table 10: Utility values assigned to (post-)surgery health states and complications/AEs

Study	Surgery	Post surgery	Complications and AEs
**Archer et al., 2016 (21)**		Post surgery: 0.70	
AbbVie submission	Surgery: 0.55	Post-surgery without compl.: 0.61	Transient compl.: 0.55<br>Chronic compl.: 0.43
MSD submission	Colectomy: 0.56; 0.56	Remission<br>- postcol.: 0.60; 0.60	Late compl. (postcol.): 0.60; 0.60
**Assasi et al., 2009 (23)**	Surgery: 0.54	Surg. Rem.: 0.82	
**Bodger et al., 2009 (31)**	Surgery: 0.73		
**CADTH, 2014 (25)**		Remission:<br>- Post-col.: 0.67	Post-col. Complication: 0.49<br>Serious AE: -0.10<br>Discontinuation due to AE: -0.10<br>Hospilatization during relapse man.: -0.05
**Dretzke et al., 2011 (26)**	Surgery: 0.52		
Abbott submission			
**Essat et al., 2014 (28)**	Surgery: 0.42	Post-surgery remission: 0.60	Post-surgery complications: 0.42<br>Serious infection: -0.52<br>Acute hypersensitivity reactions: -0.11<br>Skin site reactions : -0.03
**Kaplan et al., 2007 (32)**		Surg. Rem.: 0.86	
**Loftus et al., 2009 (33)**			
**Rafia et al., 2014 (30)**	Surgery: 0.57		Serious infection: -0.520<br>Tuberculosis: -0.550<br>Malignancy (including Lymphoma): -0.195<br>Acute hypersensitivity reactions: -0.110<br>Skin site reactions: -0.030
**Stawowczyk et al., 2016 (34)**		Remission after surgery: 0.61	Complications after surgery: 0.42
**Tang et al., 2012 (35)**		Postsurgical remission: 0.86	PML: -0.36
**Yu et al., 2009 (36)**			

PML: progressive multifocal leukoencephalopathy

2.3.6 TREATMENT EFFECT: UNDERLYING TRIALS

The treatment effect of included studies was based on a wide range of sources. An overview of trials is provided in Table 11.

Archer et al.[21] conducted a network meta-analysis (NMA) to compare the effects of adalimumab, golimumab and infliximab relative to placebo on clinical response. Such an analysis was performed for both the induction and maintenance phase. For the induction phase, data from five studies[41, 52, 53, 68] comparing two treatments were included. For the maintenance phase, both for patients starting in remission and starting in response, the NMA included data from four studies[41, 53, 56] comparing two or three treatments. In the maintenance phase, the NMA was also performed separately for the first 8–32 weeks and following 32–52 weeks. In a sensitivity analysis, the NMA was performed applying the same inclusion of trials as in the MSD submission.

In the AbbVie submission,[21] most estimates of effectiveness were taken from the ULTRA2 study and the ULTRA1/2 extension study.[41, 69, 70] Information from other references was also used to inform transitions that were not observed in these studies.[71, 72]

In the MSD submission, the manufacturer also performed a NMA to estimate the relative effectiveness of biological treatments. Archer et al. discuss this NMA and mention that for

induction, a NMA was undertaken using data from six randomized controlled trials (RCTs),[41, 52, 53, 56, 68] and for the maintenance treatment, relative treatment effects were based on a NMA of three RCTs.[41, 52, 53] However, Archer et al. critically assessed this submission and mention that the baseline model employed within the MSD NMA model was not discussed within the submissions.[44, 45] It was impossible for them to determine whether or not the applied estimates were appropriate since no additional detail was provided within the MSD submissions. Furthermore, strong assumptions were made: e.g. "*patients who have previously achieved a response can either maintain or lose that response, but they cannot improve (i.e. they cannot subsequently transit to the remission state). … no additional patients can achieve remission after induction and no patients with remission can completely lose response during any given model cycle.*"[21]

In the study of Assasi et al.,[23] the initial remission and response rates for infliximab were derived from the 12-week results of the 5 mg/kg arm that was reported by Targan et al.[73] For adalimumab, the four-week results of the 160mg and 80mg arm of the CLASSIC 1 study were used.[74] For the usual-care strategy, pooled rates from the placebo arms of these two trials were used to estimate remission and response rates. In this study, other trials were used to estimate the probability of relapse after 52 weeks of maintenance therapy. For infliximab, according to data from the ACCENT I study,[75] 37% of patients remained responders. For adalimumab, the CHARM study[39] reported that 43% of patients remained responders after 52 weeks.

Bodger et al.[31] selected the ACCENT I trial[76] to model the infliximab arm and the CHARM trial[39] for adalimumab.

The CADTH report[25] referred to an indirect treatment comparison (ITC) conducted by the manufacturer to estimate the efficacy of treatments for inducing response or remission. For the conventional treatment arm, this was based on the ACT-1, ACT-2, ULTRA-1, and ULTRA-2 trials. According to the Common Drug Review, there was no clear evidence of odds ratios being used from an ITC for the estimation of transition probabilities for golimumab, infliximab, and adalimumab. Estimation of probabilities appeared to have been achieved by pooling the number of events per single treatment arms without proper adjustment for the comparator.[25]

Dretzke et al.[26] extracted transition probabilities for the standard care states from Silverstein et al.[77] Effectiveness data for infliximab was based on the ACCENT I trial.[75, 76] Data from the CHARM trial[39] was used for adalimumab. In a sensitivity analysis, a paediatric population was modelled. In this scenario analysis, the data from the adult model was used and paediatric administration and drug costs were substituted for the adult costs.

Also the Abbott submission discussed in the report of Dretzke et al.[26] included an indirect treatment comparison. The adalimumab arm of the model was based on data up to week 56 in the CHARM trial.[39] However, since the standard arm of this trial began with adalimumab induction (80mg in week 0 and 40mg in week 2), this did not provide suitable estimates for the comparator arm.[26] Estimates for the placebo arm were based on the standard non-biologic care arm of the CLASSIC I trial.[78] An ordered probit regression prediction model was used to approximate the outcomes of the non-biologic treatment arm in the model, since the patient characteristics in the placebo arm of the CLASSIC I study were different from those in the adalimumab treatment arm of the CHARM study.[33]

In Essat et al.[28] the probabilities of remission, response (excluding remission), and no response for each medical treatment were based on the manufacturer's NMA. This NMA included the following trials for the induction phase: GEMINI1,[79] ULTRA1,[68] ULTRA2,[41] Suzuki et al,[80] ACT1,[53] ACT2[53] and PURSUIT-SC.[52] For the maintenance phase the following studies were included in the NMA: GEMINI1,[79] ULTRA2,[41] Suzuki et al,[80] ACT-1[53] and PURSUIT-M.[56]

In the study of Kaplan et al.[32], the initial response rate to dose escalation of infliximab was based on the proportion of patients in the ACCENT 1 study who lost their response to 5mg/kg, but regained it after they crossed-over to 10mg/kg.[75] The initial response rate to adalimumab was retrieved from the GAIN study[81] that evaluated adalimumab induction following infliximab failure. Estimates for remission on adalimumab at 1 year came from a subset of the CHARM study.[39] In the latter study, these data were not available for the subset of patients who were infliximab failures and the authors extrapolated the response rate of the entire study population to the infliximab failure subset.[32]

Rafia et al.[30] mention the company performed a NMA with the GEMINI II[58] and GEMINI III[59] trials as the main supporting evidence. The company also refers to the CLASSIC-I trial[78] and ENACT-1 trial[61] for adalimumab efficacy data. However, the reviewers remark that the latter trial assessed the efficacy of natalizumab for the treatment of CD, not adalimumab.[30]

Stawowczyk et al.[34] referred to the ULTRA 2 study[41] for estimates of response and remission with adalimumab or standard care.

Tang et al.[35] referred to different trials for estimates of the treatment effect with 4 different biologic treatments (see Table 11).

Finally, Yu et al.[36] relied on data from the CHARM[82] and ACCENT I[75] trials to model results for the adalimumab and infliximab treatment arm. The authors remark that patient samples were not equivalent at baseline: the CHARM trial included patients with a maximum baseline CDAI score of 450 versus 400 for ACCENT I.[36] Therefore, the sample of 234 adalimumab-treated patients were weighted to have the same baseline median, as well as the same 25th and 75th percentile CDAI values, sex distribution and median age as those in the infliximab arm of ACCENT I.[36]

Table 11: Sources for the modelled treatment effect

Study	Compared interventions	Trials
**Archer et al., 2016 (21)**	ADA, IFX, GOL, Conv., Surg.	NMA induction phase: ULTRA1, ULTRA2, PURSUIT-SC, ACT1, ACT2.<br>NMA maintenance phase: ULTRA2, PURSUIT-Maintenance, ACT1, ACT2.
AbbVie submission	ADA, Conv.	ULTRA2 trial and ULTRA1/2 extension study.
MSD submission	ADA, IFX, GOL, Surg.	NMA induction phase: ULTRA1, ULTRA2, PURSUIT-SC, PURSUIT-Maintenance, ACT1, ACT2.<br>NMA maintenance phase: ULTRA2, PURSUIT-SC, ACT1, ACT2.
**Assasi et al., 2009 (23)**	ADA, IFX, Conv.	Initial remission and response rates: Targan et al., 1997 (IFX), CLASSIC 1 (ADA).<br>Relapse while on maintenance anti-TNFs: ACCENT I (IFX), CHARM (ADA).
**Bodger et al., 2009 (31)**	ADA, IFX, Conv.	ACCENT I (IFX) and CHARM (ADA).
**CADTH, 2014 (25)**	ADA, IFX, GOL, Conv.	Indirect treatment comparison (no details provided).<br>Conventional therapy: ACT-1, ACT-2, ULTRA-1, ULTRA-2.
**Dretzke et al., 2011 (26)**	ADA, IFX, Conv.	Silverstein et al. (Conv.).<br>ACCENT I (IFX) and CHARM (ADA).
Abbott submission	ADA, Conv.	CHARM (ADA) and CLASSIC I (Conv.).
**Essat et al., 2014 (28)**	ADA, IFX, GOL, VED, Conv., Surg.	NMA induction phase: GEMINI1, ULTRA1, ULTRA2, Suzuki et al (2014), ACT1, ACT2, PURSUIT-SC.<br>NMA maintenance phase: GEMINI1, ULTRA2, Suzuki et al (2014), ACT-1, PURSUIT-M.
**Kaplan et al., 2007 (32)**	ADA, IFX	Initial response: GAIN study (ADA), ACCENT 1 (IFX).<br>Remission: CHARM (ADA), ACCENT 1 (IFX).
**Loftus et al., 2009 (33)**	ADA, Conv.	CHARM (ADA) and CLASSIC I (Conv.)
**Rafia et al., 2014 (30)**	ADA, IFX, VED, Conv.	GEMINI II and GEMINI III.<br>CLASSIC I (and ENACT-1)
**Stawowczyk et al., 2016 (34)**	ADA, Conv.	ULTRA 2.
**Tang et al., 2012 (35)**	ADA, IFX, CER, NAT	Initial response: Targan et al., 1997 (IFX), CLASSIC-I (ADA), PRECISE 1 (CER), ENACT (NAT).<br>Sustained remission: ACCENT I (IFX), CHARM (ADA), PRECISE 2 (CER), ENACT (NAT).
**Yu et al., 2009 (36)**	ADA, IFX	CHARM (ADA) and ACCENT I (IFX).

ADA: adalimumab; CD: Crohn's disease; CDAI: Crohn's disease activity index; CER: certolizumab pegol; Conv.: conventional non-biologic therapies; eow: every other week; GOL: golimumab; IFX: infliximab; NAT: natalizumab; NMA: network meta-analysis; Surg.: surgery; UC: ulcerative colitis; VED: vedolizumab

Information on other events were also often based on a wide variety of sources.

In the AbbVie submission,[21] transition complications rates were estimated from Swenson et al.[83] Chronic complication rates used information from Johnson et al.[84] (fertility), Kruasz and Duek[85] (male impotence) and Abdelrazeq et al.[86] (chronic pouchitis). Perioperative and post-operative mortality risks were based on a study published by Roberts et al.[87]

Assasi et al.[23] derived the probability of transitioning from the drug refractory health state to the surgery health state from the placebo arm in Feagan et al.,[88] (one-year major surgery rates: 0.038). Relapse after surgery was based on a review article from Lemann et al.[89] that included clinical recurrence data from the placebo arms of RCTs[90-95] that investigated the effect of 5-ASA treatment for post-surgical patients with CD on recurrence rates.

Bodger et al. [31] assumed a reduction in the risk of surgery with infliximab and adalimumab compared to control. This estimate was based on hospitalization figures reported by Lichtenstein et al.[96] and set at 0.45 in the base-case analysis.

Essat et al.[28] relied on a systematic review and meta-analysis of Froklis et al.[97] for the probability of undergoing colectomy. The surgery and post-surgery transition probabilities were estimated from the literature.[15, 98, 99] Also Rafia et al.[30] refer to the study of Froklis et al.[97] to estimate the proportion of patients undergoing surgery.

Stawowczyk et al.[34] derived the probability of colectomy from the study by Feagan et al.[100]

2.3.7 UNCERTAINTY

Almost all studies performed both probabilistic sensitivity analysis (PSA) as well as scenario analyses/one-way sensitivity analyses to estimate the uncertainty surrounding estimates of incremental costs, incremental effects and ICERs. We refer to the study of Loftus et al. for details on the PSA of the Abbott submission. Kaplan et al. did not perform a probabilistic sensitivity analysis (Table 12). In the result section, we provide an overview of the most influential variables (see part 2.3.8).

The probabilistic ICERs presented for the AbbVie submission were generated by the Assessment Group. The results of the model based on the point estimates of parameters were very similar to those produced using the probabilistic model.[21] Similar, Essat et al.[28] and Rafia et al.[30] indicate the cost-effectiveness results presented by the manufacturer were based on deterministic modelling. Whilst PSA was undertaken by the manufacturer, probabilistic ICERs were not presented within the manufacturer's submission.[28, 30] In the study of Tang et al., instead of using the mean values, the upper and lower limits of the ranges for cost data were determined by using the 1.25 and 0.75 times the median cost data.[35]

Bodger et al. also performed a threshold analysis in which they determined the treatment duration at which treatments were no longer considered to be cost effective at a threshold of £30 000 per QALY gained.[31]

Table 12: Type of analysis used to handle uncertainty

Study	PSA	scenario/one-way sensitivity analyses
**Archer et al., 2016 (21)**	x	x
AbbVie submission	x*	x
MSD submission	x	x
**Assasi et al., 2009 (23)**	x	x
**Bodger et al., 2009 (31)**	x	x
**CADTH, 2014 (25)**	x	x
**Dretzke et al., 2011 (26)**	x	x
Abbott submission	-	x
**Essat et al., 2014 (28)**	(x)**	x
**Kaplan et al., 2007 (32)**	-	x
**Loftus et al., 2009 (33)**	x	x
**Rafia et al., 2014 (30)**	(x)**	x
**Stawowczyk et al., 2016 (34)**	x	x
**Tang et al., 2012 (35)**	x	x
**Yu et al., 2009 (36)**	x	x

** The probabilistic ICERs were not calculated by the manufacturer but have been generated by the Assessment Group.*
*** Whilst PSA was undertaken by the manufacturer, probabilistic ICERs are not presented within the manufacturer's submission.[28, 30]*
PSA: probabilistic sensitivity analysis

2.3.8 RESULTS

Hereafter, we give an overview of the base-case results presented in the identified economic evaluations. An overview of these results is provided in Table 13 – Table 15. Thereafter, we provide further information on the results of the performed sensitivity analyses.

A) BASE-CASE RESULTS

Archer et al.:[21]

- When colectomy is an alternative, "*colectomy is expected to produce 14.71 QALYs at a cost of approximately £56 300 over the patient's remaining lifetime. All medical options are expected to produce substantially fewer QALYs at a greater cost than colectomy; hence, colectomy is expected to dominate IFX, ADA, GOL and conventional non-biological treatments.*"[21] The cost-effectiveness acceptability curve (CEAC) shows that for the whole range of presented willingness-to-pay (WTP) thresholds (£0-£100 000/QALY), the probability that colectomy is a cost-effective intervention approximates 100%.

  If elective colectomy is not considered an acceptable or preferable option, "*IFX and GOL are expected to be ruled out because of dominance, while the incremental cost-effectiveness of ADA versus conventional non-biological treatment is expected to be approximately £50 300 per QALY gained.*"[21] Assuming a WTP threshold of £30 000 per QALY gained, the probability that conventional management is the most cost-effective approach is approximately 98%. The WTP should increase to almost £60 000/QALY before adalimumab has the highest probability of being cost-effective.

  The authors also made an analysis for the paediatric population (mean age of 15 years), in which colectomy also dominated other interventions if considered an acceptable treatment option. If this is not an option, the ICER of IFX was calculated. However, adalimumab was not included in the analysis. Furthermore, the authors mention these analyses should be interpreted with caution since calculations were based on efficacy evidence from adult populations.

- In the AbbVie submission (marketing adalimumab – Humira®), over a 10-year time horizon, adalimumab creates 0.73 extra QALYs at an incremental cost of £25 335 per patient, which results in an ICER of £34 590/QALY gained. The probability that adalimumab is a cost-effective treatment option is 1% and 30% at a WTP threshold of £20 000 or £30 000/QALY, respectively.[21]

- In contrast, in the MSD submission (marketing infliximab/Remicade® and golimumab/Simponi®), adalimumab is expected to be dominated by golimumab, i.e. being less effective and more expensive. The estimated ICER of golimumab versus colectomy is approximately £27 000-28 000/QALY gained. The ICER of

infliximab in comparison with golimumab is expected to be approximately £76 000–80 000/QALY gained. At a WTP threshold of £30 000/QALY, golimumab has a probability of being cost-effective slightly higher than 50%.

Assasi et al.:[23]

- While usual care created the lowest expected QALYs, infliximab and adalimumab resulted in nearly identical higher QALYs. The ICER of adalimumab in comparison with usual care was estimated to be CAD193 305/QALY. When comparing infliximab with adalimumab this became CAD451 165/QALY. WTP needed to reach CAD208 000/QALY before adalimumab had a higher probability then usual care to become cost-effective.

Bodger et al.:[31]

- ICERs were calculated for every treatment option versus standard care. However, infliximab treatment was always more expensive and less effective than adalimumab treatment, thus being dominated. In Table 13, we added the ICERs calculated on the efficiency frontier, being £6850 and £13 418/QALY for one or two years respectively of maintenance treatment for initial responders to adalimumab.

CADTH:[25]

- The manufacturer's pharmacoeconomic evaluation reported the total costs and total mean utility gains per cycle over the full 10-year time horizon.[25] Median ICERs were calculated due to concerns that data was skewed (which is not in agreement with the standard approach in which the focus is on the mean values). Incremental costs, effects and ICERs are not calculated on the efficiency frontier. According to the manufacturer's calculations, golimumab has an ICER of about CAD42 000/QALY and infliximab and adalimumab are (extendedly) dominated.

Dretzke et al.:[26]

- In this study, for patients with severe disease, standard care is dominated by induction therapy, which then becomes the comparator for maintenance therapy. Infliximab and adalimumab are not mutually compared. The ICER of maintenance infliximab or adalimumab is about £5 million per QALY gained for patients with severe disease. Up to a WTP of £100 000/QALY, adalimumab or infliximab induction therapy has the highest probability of being cost-effective.

  For patients with moderate disease, standard care is not dominated by infliximab. Infliximab induction therapy has an ICER of about £94 000/QALY. In the adalimumab model, induction therapy dominates standard care and becomes the baseline. In both the infliximab and adalimumab model, the ICER of maintenance therapy was almost £14 million/QALY.

- In the Abbott submission, treatment of those with severe disease ($300 \leq$ CDAI < 450) has an ICER of about £12 000/QALY under optimistic assumptions (with about 81% of simulation being cost-effective applying a WTP-threshold of £30 000/QALY). When applying more conservative assumptions, as preferred by the review group, the ICER rises to about £30 000/QALY. The Abbott submission also calculated ICERs for patients with severe and moderate disease (not

presented here), but not for the moderate group (150 ≤ CDAI < 300) separately.

The estimates presented by the review group show an ICER of about £68 000/QALY and £113 000/QALY in the optimistic and conservative scenario, respectively. In the most favourable scenario, and applying a WTP-threshold of £30 000/QALY, only 7.9% of simulations are cost-effective.

Essat et al.:[28]

- According to the manufacturer's analysis, in the anti-TNF-α naïve population, vedolizumab dominates surgery, infliximab and golimumab. Versus adalimumab, the ICER of vedolizumab is estimated at £6634/QALY. The Evidence Review Group (ERG) notes that the manufacturer did not undertake a fully incremental analysis. Furthermore, in the ERG-preferred base case, surgery is likely to dominate all medical treatments. If surgery is not an option, according to the ERG-group, vedolizumab is expected to be dominated by adalimumab.

Kaplan et al.:[32]

- In this study with a 1-year time horizon, the infliximab dose escalation strategy yielded 0.79 QALYs compared to 0.76 QALYs for the adalimumab strategy. In combination with an extra cost of $10 293 ($28 367 per patient for infliximab dose escalation vs. $18 074 per patient for adalimumab), this results in an ICER of about $332 000/QALY.

Loftus et al.:[33]

- In this study with also a 1-year time horizon, in comparison with non-biological pharmacotherapy, adalimumab has an ICER of £16 064/QALY and £33 731/QALY in the treatment of severe or moderate-to-severe CD, respectively. The authors mention that in these populations, applying a WTP threshold of £30 000/QALY, adalimumab has an 89% and 86% probability of being cost-effective, respectively. However, we note that this latter probability of 86% is rather strange since the average ICER of about £34 000/QALY is higher than the WTP threshold.

Rafia et al.:[30]

- Over a 10-year time horizon, the probabilistic results show that adalimumab provides 0.21 additional QALYs in comparison with conventional non-biologic therapy for an additional cost of £4000, resulting in an ICER of about £19 000/QALY. Vedolizumab is extendedly dominated. Infliximab provides 0.0383 additional QALYs in comparison with adalimumab for an additional cost of about £4400, leading to an ICER of almost £116 000. Assuming a cost-effectiveness threshold of £30,000 per QALY gained, adalimumab has the highest probability of being the most cost-effective intervention (78%). For conventional therapy, infliximab and vedolizumab this is about 17%, 2.5% and 1.7%, respectively.

Stawowczyk et al.:[34]

- Over a 30-year time horizon, UC patients have 0.140 additional QALYs if treated with adalimumab and standard care instead of standard care alone. In combination with an extra cost of €10 647 (public payer perspective) or €9995 (social perspective), this results in a deterministic ICER of €76 120/QALY from a

public payer perspective and €71 457/QALY from a social perspective. The probabilistic analysis shows that a WTP >€73 800/QALY is needed to have a >50% probability that adalimumab/standard care is considered cost-effective.

Tang et al.:[35]

- In this analysis, several treatments are compared after standard care has failed. Standard care is therefore not further included as a comparator. No significant differences in efficacy are calculated between the four biologic treatments (infliximab, adalimumab, certolizumab pegol and natalizumab). They produce similar QALYs with overlapping 95%CI. Also cost estimates had overlapping confidence intervals. However, based on the Monte Carlo simulations, infliximab is dominant (i.e. better and less costly) in 39.4% (versus adalimumab), 38.0% (versus certolizumab pegol), and 78.2% (versus natalizumab) of the performed simulations.

Yu et al.:[36]

- In this study, applying a 1-year time horizon, adalimumab delivers 0.014 more QALYs (95% CI: 0.000 – 0.022) and saves $4852 (95% CI: -6758 – 491), in comparison with infliximab. Based on the probabilistic analysis, adalimumab dominates infliximab in about 94% of the simulations.

Table 13: Results presented in the identified economic evaluations (part 1/3)

Study	Results (Costs, QALYs and ICERs)					
**Archer et al., 2016 (21)**	Probabilistic cost-effectiveness results, if colectomy is an option:					
		Costs (£)	QALYs	Incr. cost (£)	Incr. QALYs	ICER (£/QALY)
	Conv. tr.	73 620	10.47	/	/	Dominated
	GOL	90 087	10.63	/	/	Dominated
	IFX	96 595	10.81	/	/	Dominated
	ADA	91 222	10.82	/	/	Dominated
	Colectomy	56 268	14.71	/	/	Dominating
	Probabilistic cost-effectiveness results, if colectomy is not an option:					
		Costs (£)	QALYs	Incr. cost (£)	Incr. QALYs	ICER (£/QALY)
	Conv. tr.	73 620	10.47	/	/	/
	GOL	90 087	10.63	/	/	Ext. dom.
	IFX	96 595	10.81	/	/	Dominated
	ADA	91 222	10.82	17 602	0.35	50 278
	Remark: analysis in paediatric population (mean age of 15 years) - ADA not included as a comparator.					
AbbVie submission		Costs (£)	QALYs	Incr. cost (£)	Incr. QALYs	ICER (£/QALY)
	Conv. tr.	NA	NA	/	/	/
	ADA	NA	NA	25 335	0.73	34 590
	Remark: Only probabilistic results generated by the Assessment Group are presented. Results based on point estimates of parameters were similar to these probabilistic results.					
MSD submission	GOL submission					
		Costs (£)	QALYs	Incr. cost (£)	Incr. QALYs	ICER (£/QALY)
	Colectomy	15 768	4.98	/	/	/
	ADA	32 097	5.49	/	/	Dominated
	GOL	31 379	5.54	15 611	0.56	27 994
	IFX	44 382	5.70	13 004	0.16	80 318
	IFX submission					
		Costs (£)	QALYs	Incr. cost (£)	Incr. QALYs	ICER (£/QALY)
	Colectomy	15 825	4.97	/	/	/
	ADA	32 123	5.48	/	/	Dominated
	GOL	31 348	5.54	15 523	0.57	27 163
	IFX	44 190	5.71	12 842	0.17	75 998
	Remark: Only probabilistic results generated by the Assessment Group are presented. Results based on point estimates of parameters were similar to these probabilistic results.					
**Assasi et al., 2009 (23)**		Costs (CAD)	QALYs	Incr. cost (CAD)	Incr. QALYs	ICER (CAD/QALY)
	Usual care	17 107	2.555	/	/	/
	ADA	45 480	2.701	28 373	0.147	193 305
	IFX	54 084	2.721	36 977	0.166	451 165
**Bodger et al., 2009 (31)**		Costs (£)	QALYs	ICER (£/QALY) vs. standard care		*ICER (£/QALY) on the eff. frontier**
	Standard care	43 490	14.209	/		*/*
	IFX (1 year)	50 330	14.568	19 050		*Dominated*
	IFX – 2 years	58 230	14.901	21 300		*Dominated*
	ADA – 1 year	46 730	14.682	7190		*6850*
	ADA – 2 years	53 090	15.156	10 310		*13418*

** Own calculation on the efficiency frontier.*
ADA: Adalimumab; CAD: Canadian dollar; Conv. tr.: Conventional non-biologic treatment; eff. frontier: efficiency frontier; Ext. dom.: extended dominated; GOL: Golimumab; IFX: Infliximab.

Table 14: Results presented in the identified economic evaluations (part 2/3)

Study	Results (Costs, QALYs and ICERs)					
**CADTH, 2014 (25)**	Manufacturer's etimates					
		Total cost (CAD) (10 year)	Mean utility (per cycle)	Incr. Cost (CAD) (per cycle)	Incr. Utilities (per cycle)	median ICER (CAD/QALY)
	Conv. tr.	131 438	0.5596	/	/	/
	GOL 50 mg	154 599	0.5733	569	0.0132	41 591
	GOL 100 mg	154 894	0.5735	585	0.0137	42 271
	IFX	161 032	0.5708	727	0.0108	65 982 (dom.)
	ADA	150 435	0.5669	463	0.0069	68 722 (ext.dom.)
	Remark: the manufacturer's pharmacoeconomic evaluation reported 3-month cycle mean incr. cost, utility gains, and median ICERs.					
**Dretzke et al., 2011 (26)**	Severe disease	Costs (£)	QALYs	ICERs (£/QALY) vs standard care	ICERs (£/QALY) on the eff. frontier	
	Standard care	13 415	0.8119	/	Dominated	
	IFX IND	12 051	0.8943	Dominates	Baseline	
	IFX MNT	19 143	0.8957	68 315	5.03 million	
	Standard care	13 421	0.8118	/	Dominated	
	ADA IND	7053	0.8942	Dominates	Baseline	
	ADA MNT	14 047	0.8956	7749	4.98 million	
	Moderate disease	Costs (£)	QALYs	ICERs (£/QALY) vs standard care	ICERs (£/QALY) on the eff. frontier	
	Standard care	6615	0.8926	/	Baseline	
	IFX IND	9573	0.9240	94 321	94 321	
	IFX MNT	16 751	0.9245	317 991	13.9 million	
	Standard care	6615	0.8922	/	Dominated	
	ADA IND	4583	0.9231	Dominates	Baseline	
	ADA MNT	11 657	0.9236	160 079	13.9 million	
Abbott submission	Optimistic estimate - severe disease					
		NHS costs (£)	QALYs	ICER (£/QALY)		
	Standard care	9892	0.7339			
	ADA	11 146	0.8384	11 998		
	Optimistic estimate - moderate disease					
		NHS costs (£)	QALYs	ICER (£/QALY)		
	Standard care	4531	0.8180			
	ADA	8540	0.8769	68 065		
	Pessimistic estimate - severe disease					
		NHS costs (£)	QALYs	ICER (£/QALY)		
	Standard care	9892	0.7339			
	ADA	12 636	0.8225	30 964		
	Pessimistic estimate - moderate disease					
		NHS costs (£)	QALYs	ICER (£/QALY)		
	Standard care	4531	0.8180			
	ADA	9333	0.8605	113 008		

ADA: Adalimumab; Conv. tr.: Conventional non-biologic treatment; eff. frontier: efficiency frontier; Ext. dom.: extended dominated; GOL: Golimumab; IFX: Infliximab; IND: induction therapy; MNT: maintenance therapy.

Table 15: Results presented in the identified economic evaluations (part 3/3)

Study	Results (Costs, QALYs and ICERs)					
**Essat et al., 2014 (28)**	Mannufacturer's estimates					
		Costs (£)	QALYs	Pairwise ICER (£/QALY) (vedolizumab vs comparator)		
	Vedolizumab	69 075	5.90	/		
	Infliximab	73 952	5.82	Dominating		
	Golimumab	70 387	5.79	Dominating		
	Adalimumab	68 157	5.76	6634		
	Conv. tr.	67 406	5.56	4862		
	Surgery	107 831	4.28	Dominating		
**Kaplan et al., 2007 (32)**		Costs ($)	QALYs	ICER ($/QALY)		
	Adalimumab therapy	18 074	0.76	/		
	Dose escalation IFX	28 367	0.79	332 032		
**Loftus et al., 2009 (33)**	Severe CD	Costs (£)	QALYs	ICER (£/QALY)		
	Nonbiologic	8992	0.7339	/		
	Adalimumab	10 882	0.8516	16 064		
	Moderate-to-severe CD	Costs (£)	QALYs	ICER (£/QALY)		
	Nonbiologic	6649	0.7743	/		
	Adalimumab	9696	0.8647	33 731		
**Rafia et al., 2014 (30)**		Costs (£)	QALYs	Incr. cost (£)	Incr. QALYs	ICER (£/QALY)
	Conv. Tr.	44 221	4.9247	/	/	/
	adalimumab	48 221	5.1390	£4,000	0.2143	£18,665
	vedolizumab	51 749	5.1431	Ext. dom.	Ext. dom.	Ext. dom.
	infliximab	52 641	5.1772	£4,420	0.0383	£115,527
**Stawowczyk et al., 2016 (34)**		Costs (€)		QALYs	ICER (€/QALY)	
	perspective:	public payer	social		public payer	social
	Standard care	9950	83 770	15.064	/	/
	ADA + standard care	20 598	93 765	15.204	76 120	71 457
**Tang et al., 2012 (35)**		Cost ($)	QALY	ICER		
	Infliximab	22 686	0.796	NA		
	Adalimumab	27 561	0.799	NA		
	Certolizumab Pegol	29 158	0.800	NA		
	Natalizumab	31 270	0.790	NA		
**Yu et al., 2009 (36)**		differences				
		Costs ($)	QALY	ICER		
	Adalimumab versus infliximab	-4852	0.014	Dominant		

ADA: Adalimumab; Conv. tr.: Conventional non-biologic treatment; IFX: Infliximab; NA: not available.

B) SENSITIVITY ANALYSIS

In this part, we describe the variables for which results are most sensitive. Table 16 gives an overview of these most determining variables as indicated by the authors of the original economic evaluations.

Treatment cost

Kaplan et al.[32] mentions the most important factor that influence the ICER in the sensitivity analysis was the drug cost for infliximab and adalimumab. Also in the study of Tang et al.[35] this is one of the most determining variables. Other analysis did not include a scenario changing drug costs.

Hospital and health state cost

In Loftus et al.,[33] results are sensitive to the hospital admission costs. An increase of 40% in these costs changes the ICER from about £16 000/QALY to making adalimumab dominant over nonbiologic therapy.

Also the authors of the AbbVie submission[21] and Essat et al.[28] report that health state costs have the largest impact on the ICER of vedolizumab.

Treatment effect

Not surprisingly, results are sensitive to changes in the modelled treatment effect.

In the analysis of Dretzke et al.[26] the relapse rate is important. In their study, sensitivity analyses are performed for patient with severe disease. Higher relapse rates increase the importance of maintenance over induction treatment. The authors mention maintenance treatment becomes more cost-effective for patients who typically suffer severe relapses within 10 or 11 weeks. However, they expect this to be a very small group of CD patients. Dretzke et al.[26] also highlights the importance of the uncertainty around the effectiveness estimates. Nevertheless, for adalimumab, the results remain robust with induction treatment dominating standard care and maintenance treatment having very high ICERs (around £500 000/QALY or higher).

In Essat et al.[28], both efficacy and remission transition probabilities have the largest impact on the ICERs. Also using response data from week 10 instead of week 6 increase the ICER of vedolizumab versus adalimumab from £6634/QALY to £21 006/QALY.

Kaplan et al.[32] indicate the initial response rate and the remission rate at 1 year are the most important model parameters that influence the QALYs.

Treatment duration

Increasing the treatment duration increases the ICERs. In Essat et al.,[28] increasing the treatment duration form one to three years increases the ICER from vedolizumab versus adalimumab from about £6600/QALY to more than £50 000/QALY.

Stawowczyk et al.[34] also report that biologic treatment is more effective but less cost-effective for the public payer and society when there is no restriction for treatment duration. Giving adalimumab with no time restriction instead of a 1-year treatment duration increases the ICER from about 76 000/QALY to almost 98 000/QALY in their analysis from the public payer’s perspective.

Utilities

In the study of Archer et al.,[21] results are sensitive to the post-surgery utility. Whereas in the base case analysis, the colectomy treatment option produces about 4 QALYs more than medical treatment options, changing the relative utilities of remission, response, active UC and post-surgery could result in the lowest QALY gain for colectomy. In all other scenarios, colectomy dominates all medical treatment options. Also the sensitivity analyses in the manufacturer’s submission indicate the sensitivity to utility assumptions.[21]

In Essat et al. using utility values from another study could change the base case ICER of vedolizumab versus adalimumab from about £6600/QALY to dominating. In Rafia et al.[30]

using utilities from another study improves adalimumab's ICER from almost £20 000/QALY to about £12 000/QALY. Also Tang et al.[35] mention the utility scores have the largest effect on the model results.

Time horizon

Changing the time horizon changes the time over which costs and effects can be accrued. In Assasi et al.,[23] the ICER of adalimumab improves from CAD304 472/QALY to CAD203 979/QALY if the 1-year time horizon is increased to 10 years.

Essat et al.[28] compares vedolizumab with all comparators. Restricting the base-case 10-year time horizon to 1 year, substantially increases vedolizumab's ICER versus adalimumab from about £6600/QALY to about £135 000/QALY. In Rafia et al.[30] the deterministic ICER improves from about £104 000/QALY, to £20 000/QALY to £10 000/QALY if the time horizon is increased from 1 year to 10 years to a lifetime horizon, respectively.

Several other studies also mention that the model results are sensitive to changing the time horizon.[25, 31]

Dretzke et al.[26] remark that the estimates of effectiveness are not changed over time since no reliable evidence on the long-term effectiveness (both the direction or magnitude of change) is available. They consider the long-term results as illustrative which should be considered with caution.

Patient weight

In Assasi et al.,[23] results are sensitive to changes in body weight. At a patient weight of 40kg, the ICER of adalimumab versus usual care and infliximab versus adalimumab is CAD172 723/QALY and CAD221 722/QALY, respectively. With a body weight of 90kg, this increases to CAD213 866/QALY and CAD681 022/QALY, respectively. The ICER in the adalimumab model changes because of patients switching to infliximab treatment in second line.

Missing value imputation method

Loftus et al.[33] is the only study that checked the sensitivity of results for using a different missing value imputation method. Instead of using the last-observation-carried-forward (LOCF) method for missing and dropped out patients, a non-biologic prediction model is applied. This increases the ICERs from about £16 000/QALY to £34 000/QALY in severe CD and from about £34 000/QALY to £58 000/QALY in moderate-to-severe CD.

Table 16: Most determining variables according to performed sensitivity analyses in the identified economic evaluations

**Study**	**Results are most sensitive for the following variables**								
	Drug treatment costs	Hospital admission cost	Health state costs*	Treatment effect**	Treatment duration	Utilities	Time horizon	Patient weight	Missing value imputation method
**Archer et al., 2016 (21)**						x			
AbbVie submission			x			x			
MSD submission						x			
**Assasi et al., 2009 (23)**				x			x	x	
**Bodger et al., 2009 (31)**					x		x		
**CADTH, 2014 (25)**							x		
**Dretzke et al., 2011 (26)**				x			x		
Abbott submission***									
**Essat et al., 2014 (28)**			x	x	x	x	x		
**Kaplan et al., 2007 (32)**	x			x					
**Loftus et al., 2009 (33)**		x					x		x
**Rafia et al., 2014 (30)**						x	x		
**Stawowczyk et al., 2016 (34)**					x				
**Tang et al., 2012 (35)**	x			x		x			
**Yu et al., 2009 (36)****									

We remark that not all studies include all these variables in their sensitivity analyses. For example, Archer et al.[21] *indicate QoL assumptions have an important impact on results, while e.g. Assasi et al.*[23] *and Bodger et al.*[31] *do not include QoL changes in their sensitivity analyses.*
** This also refers to disease state costs.*
*** This also includes the following alternative descriptions: remission transition probabilities, relapse rates, effectiveness estimates, efficacy, week 10 versus week 6 response data, initial response rate, remission rate at 1 year, and mortality estimate.*
**** We refer to the results of Dretzke et al. in which this manufacturer's submission is discussed.*
***** Univariate and multivariate probabilistic sensitivity analyses suggested that these results were robust.*

2.3.9 AUTHORS' CONCLUSIONS

In this part we give an overview of the conclusions as formulated by the authors of the identified economic evaluations. The AbbVie and MSD manufacturer's submissions do not contain an explicit conclusion. These analyses are assessed in the report of Archer et al.[21] which formulates an overall conclusion based on their own analysis. This is also the case for the Abbott submission which is assessed in the report of Dretzke et al.[26] In our discussion, we come back to the conclusions of the authors related to adalimumab.

Archer et al.:[21]

- "*The base-case analysis of the Assessment Group model suggests that within an adult UC population,* ***colectomy is expected to dominate*** *IFX, ADA, GOL and conventional non-biological treatments.* ***When elective colectomy is not an acceptable option****, the Assessment Group model suggests that IFX and GOL are expected to be ruled out because of dominance, while the incremental cost-effectiveness of* ***ADA*** *versus conventional non-biological treatment is expected to be approximately* ***£50 300 per QALY*** *gained.*"[21]
- "*The base-case analysis of the Assessment Group model suggests that* ***within a paediatric UC population****,* ***colectomy is expected to dominate IFX and conventional non-biological treatments****. When colectomy is not an acceptable option, the incremental cost-effectiveness of IFX versus conventional treatments is approximately £68 000 per QALY gained.*" We remark that in the latter paediatric analysis, adalimumab was not considered as a treatment alternative.

Assasi et al.:[23]

- "***Although infliximab and adalimumab have been shown to provide clinical benefit****, the costs associated with these treatments could be perceived as high. Based on the incremental cost-utility findings from our primary economic evaluations, adalimumab and infliximab for the treatment of IBD* ***may not be perceived to be a cost-effective use of health care resources****.*"[23]

Bodger et al.:[31]

- "*The model suggests* ***acceptable ICERs for biological agents*** *when considering a lifetime horizon with periods of up to 4 years continuous therapy.*"[31]

CADTH:[25]

- "*The issues identified by CDR* [Common Drug Review] *in the review of the manufacturer economic evaluation suggest that the included ITC* [indirect treatment comparison]*, model data transformations, underlying relationship between probability of outcome at induction and sustained outcomes at one year, and extended time horizon of 10 years may bias the results in favour of golimumab. ... CDR reanalyses varying the time horizon of the manufacturer's economic model found that the ICUR* [incremental cost-utility ratio] *for golimumab could lie in a range of $52 000 to $104 000 per QALY.*"[25]

  We remark that this conclusion is not related to adalimumab. In the manufacturer's submission related to golimumab, **adalimumab was estimated to be an extendedly dominated alternative**.

Dretzke et al.:[26]

- "*The findings of the economic model were that for induction, both adalimumab and infliximab were cost-effective (dominant relative to SC) in the management of severe CD and adalimumab was cost-effective for moderate CD (dominant relative to SC), according to the criteria laid out in the NICE Guide to the methods of technology appraisal.[101] Induction therapy with infliximab was not cost-effective for moderate CD (ICER of £94 321). Neither drug was cost-effective as maintenance therapy for moderate or severe disease (ICERs around £14M and £5M respectively for both drugs).*"[26]

Essat et al.:[28]

- Manufacturer: "*Within the anti-TNF-α naïve population, the manufacturer's model suggests that surgery is expected to be dominated by medical therapies. Vedolizumab is expected to be the most effective option. Infliximab and golimumab are expected to be dominated by vedolizumab and are ruled out of the analysis. The ICER for adalimumab versus conventional therapy is expected to be £3664 per QALY gained, whilst the ICER for vedolizumab versus adalimumab is expected to be £6634 per QALY gained.*"[28]
- ERG: "*The ERG-preferred base case indicates that **surgery is likely to dominate all medical treatments**. ... **Where surgery is not an acceptable option** in the anti-TNF-α naïve population, **vedolizumab** is expected to be **dominated by adalimumab**.*"[28]

Kaplan et al.:[32]

- "*In conclusion, among CD patients who lose response to 5 mg/kg of infliximab, **increasing the dose to 10 mg/kg** would lead to an excess of 0.03 QALY over a 1-year time frame compared to using adalimumab therapy alone. However, the difference in cost between these strategies resulted in an **ICER of over $330 000/QALY**. In sensitivity analysis, the most influential parameter estimates were the costs of adalimumab and infliximab, such that if the cost of* [IFX] *dose escalation was halved, this strategy became dominant.*"[32]

Loftus et al.:[33]

- "**Adalimumab** maintenance therapy **seems to be cost-effective** versus conventional, nonbiologic therapies for the maintenance of remission in patients with active Crohn's disease."[33]

Rafia et al.:[30]

- "*Based on the company's model, vedolizumab does not appear to have an ICER below £30 000 per QALY gained in all analyses presented by the company.*"[30]

  We remark that this conclusion is not related to adalimumab. In the probabilistic analysis, adalimumab has an ICER of about £19 000/QALY in comparison with conventional non-biological treatment.

Stawowczyk et al.:[34]

- "*Using a 30-year time horizon and the restriction for the duration of TNFα inhibitor therapy to 1 year, adalimumab/standard care treatment turned out to be more*

effective and more costly option compared with the standard care alone in Poland. ***One year biologic treatment*** *provided an* ***ICUR value of €71 457–76 120/QALY*** *gained, depending on the perspective. Biologic treatment came to be more effective but less cost-effective for the public payer and society when there is no restriction for treatment duration.*"[34]

Tang et al.:[35]

- "*Patients with moderate-to-severe Crohn's disease that failed to respond to standard treatment should preferentially receive* ***infliximab*** *as their initial biologic treatment, since this agent had the* ***highest probability of being the most cost-effective therapy*** *compared with the other biologic treatment options.*"[35]

Yu et al.:[36]

- "*This analysis suggests that adalimumab maintenance therapy is a dominant strategy versus infliximab maintenance therapy for patients with moderate to severe Crohn's disease.* ***Adalimumab appeared more effective and less costly than infliximab****.*"[36]

2.4 DISCUSSION

The overview of the economic literature allows us to identify important issues related to (the calculation of) the cost-effectiveness of adalimumab. In this part, we discuss some of these issues. These findings support us in providing input for the protocol from a health economic point of view (see chapter 3).

(Paediatric) population

All of the identified economic evaluations performed an analysis for an adult population. Two studies also included a secondary analysis for the paediatric population.[21, 26] Efficacy data, however, still relied on trials only including an adult population. The analysis also did not include the youngest children. In Archer et al.[21] the patients' starting age in their paediatric population was 15 years. The biggest change in the model was adjusting the patients' body weight and including paediatric administration costs.[26] Furthermore, these two secondary analyses only included infliximab and did not take adalimumab into account.

Dretzke et al.[26] also remark that the costs associated with treating children may differ from the costs of treating adults, due to e.g. the different drug dose and costs or the setting in which care takes place. Costs in specialist paediatric settings may be different from those that apply in adult clinics.[26]

Important remarks:

- The lack of information related to the treatment effect of biologicals in paediatric patients makes that results of such secondary analyses should be interpreted with caution.
- Archer et al.,[21] suggests RCTs assessing the clinical effectiveness of biologicals in paediatric patients as a research priority.

Severity of disease

Almost all studies explicitly include a population with moderate-to-severe CD or UC disease (see Table 4). Only two reports differentiate results according to the disease severity. Dretzke et al.,[26] inclusive the Abbott submission discussed in the same report, distinguish between severe and moderate disease. Loftus et al.[33] make calculations for severe CD and moderate-to-severe CD. They did not make a separate analysis for the moderate CD patients. Such a distinction is important in the economic evaluations since applying the same relative treatment effect to a higher baseline risk for a specific event results in a larger absolute treatment effect.

This is also reflected in the performed subgroup analyses. Dretzke et al.[26] note that the much larger health benefit for patients with severe disease compared with moderate disease results in ICERs that according to the authors were likely to be acceptable for severe disease but not for moderate disease. For example, in the Abbott submission, the ICER was about £12 000/QALY and £31 000/QALY in an optimistic and pessimistic estimate, respectively, for patients with severe disease. This was about £68 000/QALY and £113 000/QALY, respectively, in patients with moderate disease.

Important remark:

- The severity of disease might have a big impact on the ICER of biologicals. There is thus an economic justification for (not) using them as an intervention/comparator according to this severity of disease.

Adalimumab versus other biological treatment options

Based on the findings of the identified economic evaluations, adalimumab seems to have a better cost-effectiveness in comparison with other biologicals.

In Archer et al., infliximab and golimumab are expected to be ruled out because of dominance (less effective and more expensive), while the ICER of adalimumab versus conventional non-biological treatment is expected to be approximately £50 300 per QALY gained.[21] In the MSD submission (marketing infliximab/Remicade® and golimumab/Simponi®), adalimumab is expected to be dominated by golimumab.[21] However, the manufacturer’s submission includes a discount for their drug. If this discount is not taken into account, golimumab is ruled out because of extended dominance. Similarly, in the Canadian study, according to the manufacturer’s calculations, golimumab has an ICER of about CAD42 000/QALY and infliximab and adalimumab are (extendedly) dominated.[25]

In Assasi et al.[23] the ICER of adalimumab versus usual care is relatively high (about CAD193 000/QALY), but this is even higher for infliximab versus adalimumab (CAD451 000/QALY).

In Bodger et al. infliximab is dominated by adalimumab.[31]

In Dretzke et al., infliximab and adalimumab are not mutually compared. However, the findings of the economic model were in favour of adalimumab: for induction, both adalimumab and infliximab were cost-effective (dominant relative to standard care) in the management of severe CD and adalimumab was cost-effective for moderate CD (dominant relative to standard care).[26]

In Essat et al., in the ERG-preferred base case, surgery is likely to dominate all medical treatments. If surgery is not an option, according to the ERG-group, vedolizumab is expected to be dominated by adalimumab.[28]

Kaplan et al. compared adalimumab with a dose escalation of infliximab and the ICER of the latter was about $332 000/QALY.

The results of Rafia et al.[30] indicate that, assuming a cost-effectiveness threshold of £30 000/QALY, adalimumab has the highest probability of being the most cost-effective intervention (78%). Similarly, in the study of Yu et al.[36] based on the probabilistic analysis, adalimumab dominates infliximab in about 94% of the simulations.

Only in the US study of Tang et al.[35] infliximab and adalimumab are about equally effective and infliximab is cheaper.

Loftus et al.[33] and Stawowczyk et al.[34] only compared adalimumab with conventional non-biological treatment.

Based on the above information, most of the economic studies were in favour of adalimumab in comparison with other biologicals.

Important remark:

- Most of the identified studies indicate that adalimumab has a better cost-effectiveness than the other biologicals included in the analyses and thus, from a health economic point of view, seems a justified biological intervention in future trials.

Non-biological treatment options

In economic evaluations, it is important to work on the efficiency frontier, i.e. comparing treatments with the next best non-(extendedly) dominated intervention. Not all studies respect this: Bodger et al.[31] and the manufacturer's submission in the report of Essat et al.[28] did not include a fully incremental analysis. In the first study, the ICERs of all treatments are calculated versus standard care. In the second study, all treatment strategies are compared with vedolizumab. The original MSD model also did not include a fully incremental analysis, which is finally presented in the report by the reviewer group.[21]

The study of Dretzke et al.[26] illustrates the importance of working on the efficiency frontier: the ICER of adalimumab maintenance treatment versus standard care is about £8000/QALY, while in comparison with induction therapy, the ICER becomes about £5 million/QALY.

Dretzke et al.[26] also explain why it is important not to compare only biologicals with each other. This would only be relevant "*where both adalimumab and infliximab have been first justified as maintenance therapies versus standard care (SC). Where one or both maintenance therapies are not cost-effective versus SC, this comparison provides no information to decision-makers.*"[26] Therefore, Dretzke et al.[26] concentrate on the model including standard care as a treatment option. For any future trials in children, Dretzke et al.[26] suggest to include a placebo/SC arm, as there is currently no evidence of the benefit of anti-TNF therapy compared with SC.

Finally, concerning surgery, Archer et al.,[21] estimate that for patients in whom colectomy is an option, surgery dominates medical treatments. In contrast, in Essat et al.,[28] surgery was a dominated treatment strategy.

Important remark:

- From a health economic point of view, it is important to make an incremental analysis and include standard care in the analysis (and not immediately compare biologicals with each other)

Treatment effect

A major weakness of all economic evaluations is the lack of good supporting evidence to give a reliable estimate of the treatment effect. Ideally, the interventions are compared in a head-to-head RCT. As shown in Table 11, diverse trials are used to model the different treatment arms or to perform a NMA. In the report of Archer et al., the assessment group questions the selected trial data in the manufacturer's NMA, with inclusion of non-randomised data and the omission of randomised data.[21] Other models use data from separate trials to model the different treatment arms. The input for the conventional non-biological, adalimumab and infliximab treatment is often based on the CLASSIC I,[78] CHARM[39] and ACCENT I[75, 76] trials, respectively. However, comparing outcomes from individual treatment arms of separate trials might bias results and the direction of this bias is unknown.

Furthermore, the validity of the models is not always clear, and in cases were the validity has been checked, shortcomings are noticed. For example, Rafia et al.[30] note that the manufacturer's model predicts a greater number of life years in the biologic treatment arm versus non-biologic therapy, which contrasts with the lack of evidence of a differential mortality rate between treatments.[30] There was also an under-prediction of patients in remission in the placebo arm. Such discrepancies between the modelled outcomes and the observed data from the underlying trials are a major concern for the reliability of the presented results.

Important remark:

- Head-to-head RCTs are needed to allow an unbiased comparison of biologic therapy with standard care. This is also needed to set up reliable health economic models to estimate the intervention's cost-effectiveness.

Quality of life

The EUnetHTA guideline for methods for health economic evaluations recommends that results be presented in terms of both a cost-effectiveness analysis (CEA) and a cost-utility analysis (CUA).[102] The primary outcome measure(s) should where appropriate be presented as natural units (including life-years) and as QALYs.[102] The health-related quality of life (HRQoL) aspects of the QALY are captured in a HRQoL weight. Based on the review of guidelines used by EUnetHTA partners, EQ-5D is the most commonly recommended instrument for derivation of HRQoL weights, although other instruments are also mentioned (e.g. HUI, SF-6D or 15D).[102]

A major limitation is that none of the underlying trials measured QoL with a generic utility instrument. As a result, the authors of the economic evaluations have to make a lot of assumptions in their model. Archer et al.[21] derived utility estimates from a systematic review of EQ-5D studies in patients with UC (see Table 8). As such, utilities related to health states are included in the model. However, a direct measure of utility values in both the intervention and comparator treatment arm is more reliable.

Furthermore, previous reviewers also noticed strange assumptions in the utility values that are linked to health states. For example, Essat et al.[28] remark that the utility value in postsurgical remission was lower than for moderate/severe disease (0.60 versus 0.68), which appears to be inconsistent. Or Rafia et al.[30] notice that the utility score for patients with moderate to severe disease is applied to non-responders and comment that non-responders may include patients with mild disease (CDAI between 150 – 220).[30] In the absence of utility values for surgery, Dretzke et al. assumed that this health state is represented by the EQ-5D state 22222, which has a UK utility weight of 0.516.[26] Such assumptions are rather arbitrary and not very reliable.

Also the MSD (0.60) and AbbVie (0.61) submissions[21] and the Polish study[34] (0.61) include a relatively low post-surgery remission utility value. In contrast, other studies assume an (almost) equal utility value for post-surgery remission in comparison with remission under medical treatment: both 0.82,[23] or 0.86 vs. 0.89,[32, 35] respectively. Since model results are sensitive to such utility assumptions, better evidence-based input is desirable.

Finally, several studies (see part 2.3.5) derive utilities for the CD states from Gregor et al.[54] who used a standard gamble approach to define utility scores and correlate them with the CDAI. Also Bodger et al.[31] transformed CDAI scores to utilities, based on an algorithm developed by Buxton et al.[55] In this study, the correlation between CDAI and EQ-5D is -0.62 and 29% of the variability in EQ-5D scores is explained by CDAI.[55] However, Buxton et al. mention in their discussion that "*based on the variance explained, the relationships between the CDAI and utilities in the simple models are weaker than those for the IBDQ* [Inflammatory Bowel Disease Questionnaire] *and suggest that the CDAI provides a poorer basis for estimating utilities. Again its relatively poor performance as a predictor of utility reflects its main role as clinical indicator of disease activity, rather than of HRQoL.*"[55]

Important remarks:

- None of the underlying trials used to model the treatment effect of biologicals included a generic utility instrument. As a result, the economic evaluations are limited by a lack of data on HRQoL that can express outcomes in utilities.
- As recommended by the EUnetHTA guidelines on HRQoL,[103] future studies should include a generic utility instrument in complement to disease-specific questionnaires in order to adequately capture the impact of a disease on daily life.
- Including a generic utility instrument in further research is also suggested as a research priority by the reviewers in the study of Archer et al.[21] and Dretzke et al.[26] and the underlying NICE report.[27]

Indirect costs

The studies are performed from a healthcare payer's perspective, which excludes indirect non-healthcare related costs such as costs related to lost productivity. In contrast, indirect costs would represent a substantial portion of the costs of CD. A US study indicates this accounts for 28% of the total CD cost in the USA.[104]. Only two studies[33, 34] include a scenario with inclusion of these costs (see part 2.3.3). In the Polish study, based on an unpublished study, yearly indirect costs for remitted patients counted to PLN6523.75

(~€1553[l]). This was PLN22 934.58 (~€5461) for patients with active disease. Loftus et al.[33] indicate that "*including indirect costs related to lost productivity due to hospitalization improved the cost-effectiveness of adalimumab therapy. However, the estimate of indirect costs was likely substantially underestimated, because only work missed during hospitalization was included. Other indirect costs, such as decreased productivity at work and labor force nonparticipation, were not included.*" Also Assasi et al. remark that if a societal perspective was taken and indirect costs were included in the model, the cost-effectiveness of anti-TNFs compared with that of usual care likely would have been lower.[23] Unfortunately, Yu et al.[36] remark that reliable data sources do not exist to include the impact on indirect costs.

Important remarks:

- Indirect costs might represent a substantial portion of costs related to CD and UC.
- Reliable information about the impact of different treatments on indirect costs is lacking in the identified economic evaluations.

[l] The authors mention that €1=4.2PLN, based on the average exchange course from the year 2015.[34]

3 ECONOMIC INPUT FOR THE RESEARCH PROTOCOL

The review of the economic literature of adalimumab for the treatment of patients with CD or UC supports the performance of a trial with biologicals in paediatric patients, inclusive making a distinction according to severity of disease. The results of these studies also indicate that adalimumab is an appropriate intervention for inclusion in such a trial. From a health economic point of view, it is also important to make an incremental analysis comparing such an intervention with standard care, and not immediately versus another (expensive) biological treatment. A head-to-head clinical trial comparing a biological with standard care will support the calculation of the intervention's cost-effectiveness.

> "*The advent of highly effective yet costly new treatments for Crohn's disease will force clinicians, patients, and society to make important choices regarding the allocation of resources. Pharmacoeconomic analyses can be useful in deciding whether new technologies are of good value in comparison to established treatment regimens. In Crohn's disease conventional cost-effectiveness analyses are of limited use because surgery, death, and disease-related complications occur relatively infrequently. Alternatively, cost-utility models relate the incremental cost of new treatments to improvements in health-related quality of life. These analyses require the collection of valid cost and utility inputs … . Ultimately, cost-utility models should allow decision makers to make sensible choices for patients and society.*"[105]

The economic literature review identified the lack of QoL data that could be expressed as utilities and also indicated this as a research priority. Following the EUnetHTA guidelines on HRQoL,[103] such information will be included in the REDUCE-RISK trial through the inclusion of the generic EQ-5D questionnaire (part 3.1). This in complement with a disease-specific questionnaire,[m] suggested by the physician-specialists, and which is already included in the research protocol. The aim of applying the generic EQ-5D questionnaire is both 1) to assess children's (and parents') QoL, and 2) to gather this information to enable us to calculate QALYs (quality-adjusted life years) as an input for the future economic evaluation.

The review of the economic literature also indicates that indirect costs might represent a substantial portion of costs related to CD and UC but that the impact of different treatment options on such costs is lacking. Since the patient population in the REDUCE-RISK trial is restricted to children and adolescents aged 6-17 years, indirect costs do not immediately relate to the patient's productivity. Instead, we try to measure the impact on patient's school attendance and the productivity of parents (part 3.2).

[m] The included disease-specific questionnaire is the IMPACT. This is a measure of health-related quality of life, specifically designed for children with inflammatory bowel disease. It is validated for children aged 9-17 years.[106]

Furthermore, based on the review of the literature and discussions with the physician-specialists involved in the REDUCE-RISK trial, the initial treatment costs (part 3.3) and costs related to the treatment of adverse events (part 3.4) are also important to determine the treatment's cost-effectiveness.

At the end of the trial, when all information on the intervention's efficacy and safety has been gathered and the appropriate statistical analyses have been performed (part 3.5), the important incremental variables will be combined by setting up a trial-based economic evaluation to calculate the intervention's incremental costs, effects and ICERs (part 3.6).

3.1 EQ-5D

In line with the EUnetHTA and ISPOR recommendations, the EQ-5D generic utility instrument is included to assess the QoL of patients with CD or UC, as well as to provide an estimate of parents' QoL.

> "*A general recommendation applicable to all types of REA* [relative effectiveness assessment] *irrespective of their particular purpose, is to require the inclusion of a disease- or population specific and a generic HRQoL measure for most adequately capturing the impact of a disease on daily life. In case there is a need for the calculation of QALYs, a utility measure (Time Trade-Off or Standard Gamble) or generic HRQoL instrument associated with a reference set of utility values (generic utility instrument) is recommended.*"[103]
>
> "*Preference-weighted health state classification systems are more widely used in clinical trials than are direct elicitation methods such as time trade-off or standard gamble because they are both easier to administer and are considered to yield a measure of preferences from the general public. Examples of these classification systems include the EuroQol five-dimensional questionnaire* [EQ-5D]."[12]
>
> "*For countries that require an economic evaluation to support a health technology reimbursement application, it is recommended to require data emerging from the administration of a generic utility instrument in the clinical trial(s).*"[103]
>
> "*To improve comparability and consistency, countries might also consider recommending the use of one particular instrument for national reimbursement requests that is widely used (e.g. the EQ-5D).*"[103]

In our choice for an appropriate instrument, the validity, reliability, responsiveness, as well as the available languages (see part 3.1.4) and costs/fees associated with its use in our international trial are considered. Concerning the latter, the EQ-5D could be used free of costs for this study.

In patients with CD and UC, a study of Stark et al.[107] showed that both the EQ-VAS (visual analogue scale) and EQ-index scores correlate well with disease activity indices and differ significantly between active disease and remission groups. The authors concluded that the EQ-5D generates valid, reliable, and responsive preference-based evaluations of health in CD and UC. The EQ-VAS scores were more responsive than EQ-5D index scores and thus small health differences that are important from the patient's perspective may not be reflected in the EQ-index.[107] This is in line with the results from a previous study from this

research group that also concluded the EQ-5D to be "*reasonably valid, reliable and responsive in patients with inflammatory bowel disease. It can be used to generate preference-based valuations of health-related quality of life in inflammatory bowel disease.*"[108]

Furthermore, from a practical point of view, the time for completion is less than 2 minutes for the EQ-5D.[109]

The EQ-5D users guide provides some basic information about this instrument:[110]

- EQ-5D is a standardised measure of health status developed by the EuroQol Group in order to provide a simple, generic measure of health for clinical and economic appraisal.[111]
- Self-reported health status captured by EQ-5D relates to the respondent's situation at the time of completion.
- The EQ-5D consists of 2 pages - the EQ-5D descriptive system (page 2) and the EQ visual analogue scale (EQ VAS) (page 3). The EQ-5D descriptive system comprises the following 5 dimensions: mobility, self-care, usual activities, pain/discomfort and anxiety/depression.
    - In the EQ-5D-3L, each dimension has 3 levels: no problems, some problems, extreme problems.
    - In the EQ-5D-5L, each dimension has 5 levels: no problems, slight problems, moderate problems, severe problems, and extreme problems.
- The respondent is asked to indicate his/her health state by ticking (or placing a cross) in the box against the most appropriate statement in each of the 5 dimensions.
- The EQ VAS records the respondent's self-rated health on a vertical, visual analogue scale where the endpoints indicate the best and worst imaginable health state. This information can be used as a quantitative measure of health outcome as judged by the individual respondents.
- EQ-5D health states, defined by the EQ-5D descriptive system, may be converted into a utility value.
- The utilities, presented in country specific value sets, are a major feature of the EQ-5D instrument, facilitating the calculation of quality-adjusted life years (QALYs) that are used to inform economic evaluations of health care interventions.

Three EQ-5D questionnaires are used: the EQ-5D-Y (part 3.1.1), EQ-5D-Y proxy1 (part 3.1.2) and the EQ-5D-5L (part 3.1.3). A sample version of these instruments is included in Appendix 3.

3.1.1 EQ-5D-Y

> "*It is recommended that HRQoL, as a patient reported outcome, be assessed by patients themselves (self-report).*"[103]

The economic evaluation of interventions for children is complicated by the difficulty in obtaining self-reports of quality of life. As mentioned by Thorrington and Eames,[112] children may lack the cognitive ability to evaluate their health using abstract concepts in adult-specific instruments. They may also lack the required linguistic skills to answer questions. Indeed, the abstract notions contained in the EQ-5D may pose challenges for young children.[113] Therefore, the EuroQol group developed a child-friendly version of the EQ-5D-questionnaire, the EQ-5D-Y (were the Y stands for youth). It contains the same 5 dimensions as the original EQ-5D questionnaire and each dimension has 3 levels: no problems, some problems, a lot of problems. This Y-version uses a child-friendly wording. For example, 'anxiety/depression' is replaced by 'feeling worried, sad or unhappy' and the examples of usual activities is 'going to school, hobbies, sports, playing, doing things with family or friends' instead of 'work, study, housework, family or leisure activities'. Instruments that include items that are age appropriate are more likely to maximise reliability and validity of reports.[114]

In our research, the EQ-5D-Y is administered to measure the children's QoL. The EQ-5D-Y user manual mentions the **EQ-5D-Y can be used from the age of 8.**[n] The 5L version can be used for those of 12 years and over. However, the user's guide includes a possible exception: "*a study only with children up to 18 years, in this case EQ-5D-Y for older children would be recommended in order to have only one EQ-5D version in the study. The switch-over to the adult version could bring discontinuity as the adult and child versions are two different instruments.*"[115] Therefore, the youth version is used for all patients included in the REDUCE-RISK trial.

3.1.2 EQ-5D-Y PROXY1

> "*The use of proxies, such as caregivers or family, should be avoided where possible. However, the use of proxies for the measurement of HRQoL is unavoidable in some cases, e.g. cognitively impaired patients, small children.*"[103]

In the youngest children (<8 years), it is not possible to apply a self-completing questionnaire. For children aged 4-7 a proxy version can be used. The EQ-5D-Y has two proxy versions:[115]

- Proxy 1: The proxy rates how he/she rates the health of the child.
- Proxy 2: The proxy rates how he/she thinks the child would rate his/her own state if he/she were asked directly and could communicate it.

[n] We remark that in the translation certificate of the Arabic Israeli questionnaire, a higher age limit is mentioned: "*During the cognitive debriefing, it became apparent that younger children had difficulty understanding the questionnaire so it was agreed that the EQ-5D-Y version should only be used in children aged between 12-14.*" This questionnaire was tested on 8 Arabic-speaking children living in Israel aged between 8 and 14. This remark was not mentioned in the Hebrew version, which was also tested on 8 Hebrew-speaking children living in Israel aged between 8 and 15.

As recommended by the user's guide, the Proxy 1 version is applied. The user's guide recommends to apply this for children from 4-7 years and those older than 8 years who are not able to fill in the EQ-5D-Y themselves.[115] However, we ask one of the **parents** to **fill in the proxy version for all patients in the study**. This should allow us to assess the agreement between the children's and proxies estimates of the (changes in) children's QoL. "*While parents may be reliable reporters for physical activity limitations and externally manifest symptoms, their ability to accurately report on subjective outcomes such as emotion is questionable*".[113] A systematic review of studies examining the self-reported QoL in children (12 years) with congenital health conditions demonstrated that "*even for younger children, both child and parent perspectives are essential to understanding the impact of a condition on a child's QoL.*"[116] This study also examined the agreement between self- and proxy-reports. However, the main limitation was "*the lack of published studies on self-reported QoL in young children, in particular, lacking both self-reports and proxy reports.*"[116] The data from the REDUCE-RISK trial can provide input to answer this research question.

3.1.3 EQ-5D-5L

It is important to find ways of incorporating relatives' costs and effects when these might be substantial and may influence the ICERs.[117] Parents' QoL of children with CD or UC might be such an example. This has not been included in any of the identified studies, and thus the impact is unclear. Davidson et al. state that the most relevant outcome measure to use for relatives' effects would be their affected utility.[117] Therefore, we also include the measurement of parents' QoL through the use of the EQ-5D questionnaire.

In this case, there is the choice between the 3L and 5L version. The EQ-5D-5L version might be more sensitive to changes in health status in comparison with the 3L version.[118, 119] Schwenkglenks and colleagues expect that the 5L version will gradually replace the 3L version, due to reduced ceiling effects and more appropriate responsiveness.[120] Goldsmith et al. also refer to the increased ability to discriminate health states which may improve the prediction of EQ-5D index values.[121] Therefore, **to measure parents' QoL, the EQ-5D-5L version is used**.

Although there is no consensus about this topic, the study of Davidson and Levin mentions it would be beneficial if the results of the analysis were presented both with and without relatives' costs and effects.[117] In our study, results will also be presented with and without the possible impact on parents' QoL. In the first case, patient's and parents' QoL can be aggregated to estimate the total impact on QALYs of a medical intervention.[117] According to Davidson and Levin,[117] there would be no danger for double counting since the instrument used for eliciting QALY weights does not explicitly mention the relatives and are therefore probably not considered in the patient's QALY weight. The analysis without the parents' QoL allows comparing the results of the planned economic evaluation with results from other cost-effectiveness analyses.[117]

3.1.4 LANGUAGES

The initiative was taken to receive permission from the EuroQol group to use the relevant EQ-5D questionnaires to measure the impact on QoL. Table 17 provides an overview of all questionnaires that were made available for this study. Green indicates that the

questionnaire already existed. An orange cell means the questionnaire needed to be translated or reviewed by an in-(target)country linguist. The EuroQoL group took the initiative to provide validated and officially translated questionnaires.[o] In the end, for the initial group of countries considering participation in the trial, only the EQ-5D-Y proxy1 versions for the Czech Republic and Hungary were not available.

Preparatory steps are already taken in case other centres would also participate in the study: the three EQ-5D questionnaires are available for Australia, Japan, Portugal, South Korea and Spain. The Y-version is also available for Greece and Malaysia.

Table 17: Overview of available EQ-5D questionnaires

**Original countries participating in the trial**				
**Country**	**Languages**	**EQ-5D-5L**	**EQ-5D-Y**	**EQ-5D-Y proxy1**
Belgium	French, Dutch	OK (2x)*	OK (2x)	OK (2x)
Canada	French, English	OK (2x)	OK (2x)	OK (2x)
Czech Republic	Czech	OK	OK	/
France	French	OK	OK	OK
Germany	German	OK	OK	OK
Hungary	Hungarian	OK	OK	/
Israel	Hebrew, Arabic	OK (2x)	OK (2x)	OK (2x)
Italy	Italian	OK	OK	OK
Poland	Polish	OK	OK	OK
The Netherlands	Dutch	OK	OK	OK
UK	English	OK	OK	OK
**Countries possibly participating in the future**				
Australia	English	OK	OK	OK
Croatia	Croatian	OK	/	/
Greece	Greek	OK	OK	/
Ireland	English	OK	/	
Japan	Japanese	OK	OK	OK
Malaysia	English, Malay, Simplified Chinese, Tamil	OK (4x)	OK (2x)**	
Portugal	Portuguese	OK	OK	OK
South Korea	Korean	OK	OK	OK
Spain	Spanish	OK	OK	OK

** (2x) refers to the two language versions that are available for these multilingual countries.*
*** For Malaysia, the EQ-5D-Y version is available in Malay and Simplified Chinese.*
In green: questionnaires that already existed; In orange: questionnaires for which a review by

[o] The translation process includes the following steps: 1) translation of the source text into the target language by a professional translator (Single Forward Translation); 2) This forward translation is back translated into its original language by another professional translator (Single Back Translation); 3) This back translation is reviewed against the source text by the project manager (Back Translation Review); 4) The EuroQol group and partners discuss the back translation review report (EuroQol Review); 5) Proofreading for accuracy and layout of the translation (Proofreading and finalization); 6) Review of the proofreading changes by EuroQol (EuroQol Review).(Source: Personal communication with the EuroQol Group)

an in-(target)country linguist or a full translation was performed. In yellow: translation and validation process is ongoing.

3.1.5 TIMING

All children that are able to self-report QoL fill out the EQ-5D-Y. The EQ-5D-Y proxy1 should always be filled out by one of the parents, also when the child is able to fill out the EQ-5D-Y, to have a proxy estimate of the children's QoL (see part 3.1.2). The parents self-report their QoL filling out the EQ-5D-5L questionnaire.

The EUnetHTA guidelines recommend to demonstrate the HRQoL benefits of an intervention by means of repeated measurements in both the intervention and the control group.[103] As recommended by the ISPOR guidelines,[12] baseline measures of HRQoL are collected. The timing of the other measurements is mentioned in Table 18, both for the RCT and the inception cohort. In the RCT, QoL measurements are made at baseline and all following planned study visits (months 2, 4, 6, 9 and 12). In the inception cohort, the EQ-5D is included at baseline, months 3, 6, 12 and 18 and thereafter annually.

Table 18: Overview of the EQ-5D measurements (RCT and inception cohort)

**RCT**								
Time	w-3	M0*	M2	M4	M6	M9	M12	
Visit**	V0	V1	V2	V3	V4	V5	V6	
EQ-5D quest.	/	+	+	+	+	+	+	
**Inception cohort**								
Time	M0*	M1	M3	M6	M12	M18	M24-...	
Visit	V0	V1	V2	V3	V4	V5	Annual	Unsch.
EQ-5D quest.	+	/	+	+	+	+	+	/

** this is the baseline measurement.*
*** All follow-up visits can be scheduled within a +/-2weeks window.*
M: month; Quest.: questionnaires; Unsch.: unscheduled visits; V: visit.
Remark: in a first version of the protocol, it was mentioned that the EQ-5D measurements were not mandatory at M4 and M9, but recorded if available. However, in order to avoid much missing information, which is difficult to handle afterwards, it was decided to include these measurements for all patients as mentioned in the above table.

3.1.6 PRESENTING EQ-5D RESULTS AND VALUE SETS

Results will be presented transparently in tables and figures. Response rates and missing information will be mentioned. The user's guideline mentions the collected data can be presented in various ways:[103]

- Health profiles: Presenting results from the EQ-5D descriptive system as a health profile (i.e. the proportion of reported problems for each dimension).
- EQ VAS: Presenting results of the EQ VAS as a measure of overall self-rated health status. Both a measure of the central tendency and a measure of dispersion will be presented (i.e. mean values and 95% confidence intervals)

- EQ-5D index value – utilities: Presenting results from the EQ-5D index value. The collection of index values for all possible EQ-5D states is called a value set.

The EQ VAS self-rating records the respondent's own assessment of their health status and are therefore not representative of the general population. Value sets are based on VAS or TTO valuation techniques, and reflect the opinion of the general population.[110] The review of guidelines used by EUnetHTA partners shows that most guidelines recommend a value set based on hypothetical preferences representing the general public.[102]

> "*Country-specific value sets should be applied and reported in each pharmaco-economic report. This is no different from the requirement to use country specific costs. In the absence of a country-specific value set, the researcher should select another set of values for a population that most closely approximates that country. Sometimes however, information about index values ('utilities') is required to inform researchers or decision makers in an international context. In these instances, one value set applied over all health states data is probably more appropriate.*"[110]

A) EQ-5D-5L

For the EQ-5D-5L, value sets are currently available for 3 countries included in our trial: Canada, England and the Netherlands (https://euroqol.org/eq-5d-instruments/eq-5d-5l-about/valuation/). These different value sets will be used in a sensitivity analysis to check the robustness of results. For several countries were no value set is (yet) available, the EuroQol group created a crosswalk value set. This is a set that has been created for the 3L version and is adapted to fit the 5L version (https://euroqol.org/eq-5d-instruments/eq-5d-3l-about/valuation/choosing-a-value-set/). At the end of the trial, we will check if value sets for other countries became available. If not, the crosswalk value set will be used in the sensitivity analysis. Another possibility is the recently developed western preference pattern (WePP) model that has been developed and suggested as a useful "common currency" for (Western) countries that have not yet developed their own value sets.[122] This can also be used as an alternative.

Furthermore, we remark that the "*NICE guide to the methods of technology appraisal 2013[123] states that data collected using the EQ-5D-5L descriptive system may be used for reference-case analyses. When the guide was written, there was no valuation set for EQ-5D-5L from which to derive utilities. NICE's methods guide (section 5.3.12) states that: 'Until an acceptable valuation set for the EQ-5D-5L is available, the validated mapping function to derive utility values for the EQ-5D-5L from the existing EQ-5D (-3L) may be used'.*"(available from http://www.euroqol.org). A valuation set is now available for the EQ-5D-5L questionnaire that reflects the preference of members of the public in England.[124] However, NICE has issued a position statement on this valuation set, including among others the following:[125]

- "*Currently the 5L valuation set is not recommended for use. Companies, academic groups, and others preparing evidence submissions for NICE should:*
    - *Use the 3L valuation set for reference-case analyses*

  - *Whilst several mapping functions are available (Hernandez Alava et al. 2017), for consistency with the current guide to the methods of technology appraisal, the mapping function developed by van Hout et al. (2012)[126] should be used for reference-case analyses.*
  - *NICE supports sponsors of prospective clinical studies continuing to use the 5L version of EQ-5D descriptive system to collect data on quality of life.*
- *NICE plans to review this statement in August 2018.*"

The status of this NICE statement and the presence of other value sets will be checked when performing the economic evaluation. The most robust country-specific value set will be used in the reference case and other relevant value sets will be used in sensitivity analysis.

B) EQ-5D-Y (PROXY1)

The value sets for the EQ-5D are generated in an adult population. Adult preferences for health states may be different from the preferences of children and adolescents.[112] The user's guide for the youth version mentions that "*at present, a value set for the EQ-5D-Y is not yet available. It is not recommended to use the 3L value set as proxy value set for the EQ-5D-Y. The EuroQol Group is currently working on the development of a protocol for the valuation of the EQ-5D-Y.*"[115]

Thorrington and Eames performed a systematic review of the literature on measuring health utilities in children and adolescents. They identified several examples[127-129] discussing the lack of an appropriate value set for the EQ-5D-Y. It is stated that the current youth version of the EQ-5D is not yet complete without a child-focused value set. In the identified examples, existing value sets have been taken from the 3L-version of the adult-specific EQ-5D.[112] When performing the economic evaluation, the presence of a child-specific value set will be checked. Otherwise sensitivity analysis will be performed using both the adult tariffs and VAS outcomes.

3.2 SCHOOL ATTENDANCE AND PRODUCTIVITY QUESTIONNAIRES

In the literature review of the economic evaluations, we remark that indirect costs might represent a substantial portion of costs related to CD and UC (see part 2.4). Unfortunately, reliable information about the impact of different treatments on indirect costs is lacking in the identified economic evaluations. In adult populations, researchers often refer to the impact on productivity. For our paediatric population, we think in the first place about the school attendance of children and the possible impact of the child's disease on their parents' productivity.

To improve the quality and uniformity of data generated from trials, the ISPOR guidelines[12] recommend to use validated instruments when incorporating productivity costs.[130-132] Using validated and reliable instruments if available is a general rule. Therefore, a "quick and dirty" search was performed to identify suitable validated questionnaires for both school attendance (part 3.2.1) and parents' productivity (part 3.2.2).

3.2.1 SCHOOL ATTENDANCE

For school attendance, the following terms are combined: "Inflammatory Bowel Diseases"[Mesh] AND (("schools"[MeSH Terms] OR "schools"[All Fields] OR "school"[All Fields]) AND attendance[All Fields]). After an initial search in November 2016, this search is updated in January 2018 and yielded only 13 references of which three are considered relevant.[133-135]

One study used a semi-structured questionnaire for both children and parents and found that significant psychosocial and academic difficulties are faced by children with chronic diseases like IBD.[133] Children with CD and UC missed significantly more school days than age-matched healthy controls.[133] Another study[134] created an online survey that included a Student Adaptation to College Questionnaire (SACQ). The results show that "*disease activity in students with CD was associated strongly with their self-reported ability to keep up with academic work (P<.0089) and confidence in their ability to meet future academic challenges (P<.0015). Students with active IBD reported feeling as if they were not academically successful (P<.018), and students with ulcerative colitis reported irregular class attendance (P<.043).*"[134] The authors conclude that "*students with IBD do not adjust to college as well as healthy students*" and that "*strategies to increase disease control and provide social and emotional support during college could improve adjustment to college and academic performance, and increase patients' potential.*"[134] The third study obtained report cards and school absence information from schools. Children with IBD had poorer school functioning and significantly more absences.[135] However, demographic and psychosocial factors seem to be better predictors than disease factors.[135]

None of these studies used a structured questionnaire that was validated for use in children with CD or UC. The SACQ questionnaire[p] is a 67-item, self-report questionnaire that is for

[p] Source: https://www.wpspublish.com/store/p/2949/sacq-student-adaptation-to-college-questionnaire

college students and is mainly used at universities for routine freshman screening. It is considered not appropriate for our research.

A non-systematic google search is performed to identify other potentially relevant questionnaires. However, these questionnaires are very general. For example, the 'School Attendance Questionnaire'[q] mentions these questionnaires are generally designed by school authorities to find out the reasons for missing school. However, the questions posed clearly indicate this questionnaire of school attendance is not well placed to apply in a population of sick children (e.g. 'are your parents aware of this attendance percentage' or 'are you aware that … can lead to your suspension from school'). Other researchers propose a novel method for measuring class attendance by using location and bluetooth data collected from smartphone sensors.[136] This is not applicable for the youngest children in our population since they don't have a smartphone. No well suited questionnaire is thus identified that can be used for this international trial.

De novo school attendance questionnaire

A non-validated school attendance questionnaire is set up. This choice was made since we preferred to take the initiative to try to measure the impact with a non-validated instrument instead of not trying to measure this important aspect. The school attendance questionnaire exists of a version that is used at the start of the research and a version to be used at the follow-up visits:

- Questionnaire school attendance - start of the research (see Appendix 4)
- Questionnaire school attendance - follow-up visits (see Appendix 4)

The questionnaire is initially set up in Dutch. Due to time restrictions and the approaching start of the trial, no forward and back translation is foreseen by a professional translator. Initially, a translation is made in French and English by native speakers involved in this international H2020 project. The English version was then used to translate the questionnaire in other languages (e.g. Hebrew).

In our questionnaire, we avoid questions that are country specific (e.g. related to the school system) since this would cause problems in both the applicability of the questionnaire in our international trial and the analysis of results. The parents fill out this questionnaire. First, we ask them to give a general picture of a typical school week to be able to have a view on the number of days the child goes to school in a typical week (exclusive home education) and the presence of home schooling (or home education). The aim of the questionnaire is to estimate the impact of IBD (CD and UC) and its treatment on school attendance and home education. We will measure:

- The presence and amount of home education.
- Whether home education is due to IBD.
- The percentage of school days that children could not attend[r]

---

[q] Source: http://www.samplequestionnaire.com/school-attendance.html

[r] % = 'How many school days could your child not attend?' / ('How many school days could your child not attend?' + 'How many school days could your child attend during this period?')

- In case of home education, the percentage of home schooling days that children could not attend[s]
- For both school days and home education, the part of absence that is due to IBD (in the opinion of the parents)[t]

To assist participants with accurate recall, the ISPOR guidelines[12] recommend economic investigators to consider using memory aids such as diaries to record medical visits and events, and should inform participants that they will be asked to report this information throughout the trial.[137] In line with this recommendations, to help the parents, the questionnaires (at the start + during follow-up visits) include an overview of the questions that will be posed at the follow-up visits. The last page, entitled '*information for parents to take home to help in collecting information for the next follow-up visit*', contains an overview of the questions. A simple diary is considered a useful tool and is suggested to the parents to document presence/absence from school as well as their judgement on whether this is IBD-related or not.

The timing of the measurements is provided in Table 19, for both the RCT and the inception cohort. In the RCT, the timing coincides with the EQ-5D measurements (see above in Table 18). In the inception cohort, the school attendance questionnaire is included at baseline, months 3 and 12 and thereafter annually. Month 3 is also included since this is the end of the induction phase.

Table 19: Overview of the school attendance measurements (RCT and inception cohort)

**RCT**								
Time	w-3	M0*	M2	M4	M6	M9	M12	
Visit	V0	V1	V2	V3	V4	V5	V6	
School att. quest.	/	+	+	+	+	+	+	
**Inception cohort**								
Time	M0*	M1	M3	M6	M12	M18	M24-…	
Visit	V0	V1	V2	V3	V4	V5	Annual	Unsch.
School att. quest.	+	/	+	/	+	/	+	/

[s] % = 'How many home schooling days could your child not attend?' / ('How many home schooling days could your child not attend?' + 'How many home schooling days could your child attend during this period?')

[t] % for school days = 'How many of these days … do you think your child could not attend due to the inflammatory bowel disease?' / 'How many school days could your child not attend?'

% for home schooling days = 'How many of these days … do you think your child could not attend due to the inflammatory bowel disease?' / 'How many home schooling days could your child not attend?'

** this is the baseline measurement.*
*** All follow-up visits can be scheduled within a +/-2weeks window.*
M: month; Quest.: questionnaires; Unsch.: unscheduled visits; V: visit.

The results of the school attendance questionnaire will be presented in table format. In the economic evaluation, the impact on school attendance will be included if there is an incremental impact on this outcome. This will be included as a non-monetary consequence, separately from the ICER estimates.

3.2.2 PARENTS' PRODUCTIVITY

In search for a suitable productivity questionnaire that measures absenteeism, the following MeSH (Medical Subject Headings) terms are used to identify an existing systematic review (date of search: 14 January, 2018): ("Surveys and Questionnaires"[Mesh] AND "Absenteeism"[Mesh]) AND ("Review Literature as Topic"[Mesh] OR "Review" [Publication Type]). As such 131 references are identified. One of the studies contains a review of methods to measure health-related productivity loss and identified 20 survey instruments that assess the effect of health problems on absenteeism or presenteeism.[138] More recently developed questionnaires not included in this review are the iMTA Productivity Cost Questionnaire (iPCQ),[139] the Work Role Functioning Questionnaire,[140] and the World Health Organization Health and Work Performance Questionnaire (HPQ) (https://www.hcp.med.harvard.edu/hpq/).

None of the identified questionnaires is suitable to measure in an international trial the impact of a child's disease on their parents' productivity. Since we initially thought no such validated questionnaire was available, we set up a de novo questionnaire. However, when writing this report, we found out that there already exists such a questionnaire, the WPAI:CD-CG and WPAI:UC-CG (see hereafter). At the annual meeting in Paris (25-26 January, 2018), we suggested to use this already existing questionnaire. We reduced the de novo 'school attendance and productivity questionnaire' to the previously presented 'school attendance questionnaire'. For transparency, we present the deleted parts that were originally included in Appendix 5. Deleting these questions did not influence any of the school attendance-related questions nor the order of these questions.

WPAI:CD-Caregiver and WPAI:UC-Caregiver

The Work Productivity and Activity Impairment questionnaire (WPAI) (http://www.reillyassociates.net/) is a self-administered questionnaire assessing the impact of a disease on a patient's ability to work and/or perform non-work activities. There exists a version specifically for CD (WPAI:CD) and for UC (WPAI:UC). The RCT is restricted to CD patients, while the inception cohort includes both CD and UC.

The discriminative validity, reliability, and responsiveness of the WPAI:CD were demonstrated in an RCT of 662 CD patients in whom certolizumab pegol was compared with placebo.[141] The Spanish WPAI:CD questionnaire was also judged to be a valid and reliable measurement of work impairment in Crohn's disease. However, in this study, the test unexpectedly did not present satisfactory reproducibility for the evaluation of presenteeism and asked for further research.[142] However, in another study, the same research team evaluated the validity and reproducibility of the Spanish version of the WPAI questionnaire in

CD patients and confirmed its validity for measuring work impairment in CD patients. In this study, the test reproducibility was also adequate.[143]

There also exists a caregiver version of the WPAI in which the effect of a child's specific health problem on the parent's work productivity is measured. The caregiver version for CD is included in Appendix 6. It includes six questions that ask about the effect of a child's CD on their parents' ability to work and perform regular activities:[u]

- Q1 = currently employed
- Q2 = hours missed due to problems associated with child's CD
- Q3 = hours missed for other reasons
- Q4 = hours actually worked
- Q5 = degree child's CD affected productivity while working
- Q6 = degree child's CD affected regular activities

The following scores are calculated from these questions:[u]

- Percent work time missed due to child's CD: Q2/(Q2+Q4)
- Percent impairment while working due to child's CD: Q5/10
- Percent overall work impairment due to child's CD: Q2/(Q2+Q4)+[(1-(Q2/(Q2+Q4)))x(Q5/10)]
- Percent activity impairment due to child's CD: Q6/10

It thus includes a measurement for both absenteeism (percentage of time work missed) and presenteeism (percentage of impairment while working) for parents of a child with CD. The third score indicates the percentage of activity impairment (total work productivity impairment; TWPI). The last score provides an overall percentage of work impairment (total activity impairment; TAI).[130]

The recall period in the WPAI questionnaire and its validation studies is 7 days. The general literature on recall burden suggests that a longer recall period would not be suitable for the type of information being elicited with the WPAI.[u]

The WPAI has been translated into several languages through a harmonization process consisting of several independent translations, back translations, expert review of the back translation, and local review by users.[u] The available WPAI:CD-CG versions for the countries involved in the RCT are mentioned in Table 20. For the inception cohort, if the diagnosis is UC, the WPAI:UC-CG versions area applied (Table 21).

Table 20: Overview of available WPAI:CD-CG questionnaires (Crohn's Disease, for caregivers)

**Original countries participating in the trial**		
**Country**	**Languages**	
Belgium	French	WPAI:CD-CG (French-France, V2.0)
	Dutch	WPAI:CD-CG (Dutch-Netherlands, V2.0)
Canada	French	WPAI:CD-CG (French-Canada, V2.2)

[u] Source: http://www.reillyassociates.net/

	English	WPAI:CD-CG (English-UK, V2.0)
Czech Republic	Czech	WPAI:CD-CG (Czech-Czech Republic, V2.1)
France	French	WPAI:CD-CG (French-France, V2.0)
Germany	German	WPAI:CD-CG (German-Germany, V2.1)
Hungary	Hungarian	/
Israel	Hebrew	WPAI:CD-CG (Hebrew-Israel, V2.0)
	Arabic	WPAI-CD-CG (Arabic-Israel, final)
	Russian	WPAI-CD-CG (Russian-Israel, V2.2)
Italy	Italian	WPAI:CD-CG (Italian-Italy, V2.3)
Poland	Polish	WPAI:CD-CG (Polish-Poland, V2.0)
The Netherlands	Dutch	WPAI:CD-CG (Dutch-Netherlands, V2.0)
UK	English	WPAI:CD-CG (English-UK, V2.0)
**Countries possibly participating in the future**		
Australia	English	WPAI:CD-CG (English-Australia, V2.0)
Croatia	Croatian	WPAI:CD-CG (Croatian-Croatia, V2.0)
Greece	Greek	WPAI:CD-CG (Greek-Greece, V2.2)
Ireland	English	WPAI-CD-CG (English-Ireland, V2.0)
Japan	Japanese	/
Malaysia	Malay	/
Portugal	Portuguese	WPAI:CD-CG (Portuguese-Portugal, V2.1)
South Korea	Korean	/
Spain	Spanish	WPAI:CD-CG (Spanish-US, V2.0)

In green: questionnaires that already existed; In orange: questionnaires for which a version with the appropriate language but from another country is available. /: no questionnaire is available for these countries.

Table 21: Overview of available WPAI:UC-CG questionnaires (Ulcerative Colitis, for caregivers)

**Original countries participating in the trial**		
**Country**	**Languages**	
Belgium	French	WPAI:UC-CG (French-Belgium, v2.2)
	Dutch	WPAI:UC-CG (Dutch-Belgium, v2.2)
Canada	French	WPAI:UC-CG (French-Canada, v2.2)
	English	WPAI:UC-CG (English-US, v2.0)
Czech Republic	Czech	WPAI:UC-CG (Czech-Czech Republic, v2.4)
France	French	WPAI:UC-CG (French-France, v2.2)
Germany	German	WPAI:UC-CG (German-Germany, v2.1)
Hungary	Hungarian	WPAI:UC-CG (Hungarian-Hungary, v2.1)
Israel	Hebrew	WPAI:UC-CG (Hebrew-Israel, v2.0)
	Arabic	WPAI:UC-CG (Arabic-Israel, v2.0)
Italy	Italian	WPAI:UC-CG (Italian-Italy, v2.3)
Poland	Polish	WPAI:UC-CG (Polish-Poland, v2.1)
The Netherlands	Dutch	WPAI:UC-CG (Dutch-Netherlands, v2.0)
UK	English	WPAI:UC-CG (English-US, v2.0)
**Countries possibly participating in the future**		
Australia	English	WPAI:UC-CG (English-US, v2.0)
Croatia	Croatian	/
Greece	Greek	/
Ireland	English	WPAI:UC-CG (English-US, v2.0)
Japan	Japanese	WPAI-UC-CG (Japanese-Japan, V2.2)
Malaysia	Malay	/
Portugal	Portuguese	/

South Korea	Korean	/
Spain	Spanish	WPAI:UC-CG (Spanish-Spain, v2.1)

In green: questionnaires that already existed; In orange: questionnaires for which a version with the appropriate language but from another country is available. /: no questionnaire is available for these countries.

The timing of the measurements is provided in Table 22, for both the RCT and the inception cohort, and coincides with the measurements for the school attendance questionnaires (see above in Table 19).

Table 22: Overview of the WPAI:CD-CG measurements (RCT and inception cohort)

RCT								
Time	w-3	M0*	M2	M4	M6	M9	M12	
Visit	V0	V1	V2	V3	V4	V5	V6	
WPAI:CD-CG quest.	/	+	+	+	+	+	+	
**Inception cohort**								
Time	M0*	M1	M3	M6	M12	M18	M24-...	
Visit	V0	V1	V2	V3	V4	V5	Annual	Unsch.
WPAI:CD-CG quest.	+	/	+	/	+	/	+	/

** this is the baseline measurement.*
*** All follow-up visits can be scheduled within a +/-2weeks window.*
M: month; Quest.: questionnaires; Unsch.: unscheduled visits; V: visit.

Similar as for the results of the school attendance questionnaire, the scores of the WPAI:CD-CG questionnaire applied in the RCT will be presented in table format. In the economic evaluation, the impact on productivity will be included if there is an incremental impact on this outcome. The monetary value of productivity losses will be included according to the national guidelines on economic evaluation (if such specific guidelines for productivity losses exist[v]). Both an analysis with and without this impact will be presented.

[v] For example, the Belgian guidelines for economic evaluations mention the Belgian average labour cost per working day was estimated at €257 (costing year 2010). The guidelines also mention short-term lost productivity during paid work has to be valued using the Human Capital Approach. Long-term absence from work should be valued applying the Friction Cost Method, varying the friction period from 2 to 6 months.[144]

3.3 TREATMENT COSTS

The ISPOR guidelines recommend "*prioritization of high-cost resources as well as those that are expected to differ between treatment arms, without distinction as to whether they are related to disease or intervention[145]. The scope of resources considered should include direct medical and nonmedical resources and indirect or productivity costs across patients and caregivers.*"[12] Possible differences in productivity in the RCT will be measured through the WPAI:CD-CG questionnaire (see previous part). High-cost resources that are expected to differ between treatment arms are of course the drug treatment costs in the different treatment arms. The protocol of the REDUCE-RISK trial mentions the following:[w]

- "*Low risk protocol*"
    - *Subcutaneous MTX once weekly 15mg/m² body surface area,[146, 147] with a maximal dose of 25mg/week (low dose therapy). Ondansetron (Zofran) premedication (4-8mg 1H prior to injection) is recommended, folate acid substitution (15mg po, 3 days after MTX injection, for children <20kg: 1x 5mg) is recommended. (MTX sc. injections are performed by a qualified health professional, only the injectable forms of MTX are used)*

      versus
    - *Oral AZA/6MP at a dose of 2.5 mg/kg once daily rounded to the nearest multiplication of 12.5mg or oral 6MP at a dose of 1.5mg/kg once daily rounded to the nearest multiplication of 12.5mg. Heterozygote patients for TPMT or those with TPMT activity 6-9nmol/h/ml erythrocytes (9-22nmol 6MTG/g Hb/h will receive half the calculated dose. TPMT homozygotes or those with TPMT activity <6 nmol/h/ml erythrocytes (or <9 nmol 6MTG/g Hb/h) will be excluded from the trial.*
- *High risk protocol*
    - *Subcutaneous MTX once weekly 15mg/m2 body surface area,[146, 147] with a maximal dose of 25mg/week (low dose therapy). Ondansetron (Zofran) premedication (4-8mg 1H prior to injection) is recommended, folate acid substitution (1x 15mg po, 3 days after MTX injection, for children <20kg: 1x 5mg) is recommended. (MTX sc. injections are performed by a qualified health professional, only the injectable forms of MTX are used)*

      versus
    - *Subcutaneous Adalimumab started at a dose of 160mg followed by 80mg 2 weeks later and then 40mg every 2 weeks in patients over 40kg. In patients < 40kg sc. doses of Adalimumab are as follows: induction 160mg/1,73m2 BSA (max 160mg), followed by 80mg/1,73m2 BSA (max 80mg) 2 weeks later and maintenance of 40mg/1,73m2 BSA (max 40mg) every 2 weeks, all doses rounded up to the nearest 5 multiplications. (ADA*

[w] Source: Protocol REDUCE RISK 2016.10.24 V3.3

sc. injections are performed by either a qualified health professional or a trained parents or patient)"

For cost calculations, the administered doses and treatment duration will be used to determine the amount of administered drugs. This will be multiplied with the official list prices for these drugs. Possible waste for adalimumab[x] will also be taken into account. Scenarios will be performed to take into account the price differences between countries. The influence of possible discounts or the introduction of biosimilars on the official list prices will be modelled trough scenario analyses.

3.4 ADVERSE EVENTS AND RELATED COSTS

The ISPOR guidelines mention that "*the frequency with which resource use data are collected should account for the levels of resource usage expected among patients enrolled in the trial, the ability to verify patient-reported data through electronic medical records or other sources, and the characteristics(e.g., cognitive abilities) of the trial participants.*"[12] The guidelines also "*caution against narrow collection of resources given that the treatment may have unanticipated effects and the trial may offer the last opportunity to collect these data within a randomized study design*".[12] On the other hand, we do not want to overload the researchers involved in the trial with gathering too much information. For adverse events, it is difficult to predict which ones will differ between treatment arms. Gathering the costs for all adverse events is considered to be a major effort that might miss its goal: in case there are no incremental differences, these costs will not influence the ICERs. Therefore, a more practical approach is choses. For the medical part of the REDUCE-RISK trial, information on the occurrence of an adverse event (AE) or serious adverse event (SAE)[y] will be gathered. The investigator will use the following definitions to rate the severity of each adverse event:[w]

- Mild: The adverse event is transient and easily tolerated by the subject.
- Moderate: The adverse event causes the subject discomfort and interrupts the subject's usual activities.
- Severe: The adverse event causes considerable interference with the subject's usual activities and may be incapacitating or life-threatening.

The investigators will also assess the relationship of the adverse event to the use of study drug. For the economic evaluation, we will include the adverse events for which significant differences between the treatment arms are identified and which entail important treatment costs. For the treatment costs of these incremental adverse events, we will have a look at administrative billing information or set up the theoretical treatment costs. In order to have country-specific information, collaboration with several research teams from a selection of countries will be necessary. The countries are not selected yet, but we will select them based

[x] Children with a body weight >40 kg receive 40mg. Under a body weight of 40kg, less than the 40mg vial dose is administered.

[y] According to the protocol, the following adverse events are regarded as serious adverse events: death of subject, life-threatening, hospitalization, prolongation of hospitalization, congenital anomaly, persistent or significant disability/incapacity, important medical event requiring medical or surgical intervention to prevent serious outcome, spontaneous abortion, elective abortion, pregnancy. We refer to the full protocol for further details.

among others on the number of patients included in the trial as well as the ability of the research team to collaborate in gathering country-specific cost information.

3.5 STATISTICAL ANALYSIS

Data will be entered into an eCRF (electronic case report forms – designed for this trial) by each individual investigator on site of the patient inclusion, monitoring will be assured by PIBDnet, as well as data clearance and calculation.[w]

The ISPOR guidelines mention that:

- "*The analysis of economic measures should be guided by a data analysis plan. A prespecified plan is particularly important if formal tests of hypotheses are to be performed. Any tests of hypotheses that are not specified within the plan should be reported as exploratory. The plan should specify whether generalized linear model, least squares regression, or other multi-variable analysis will be used to improve precision and to adjust for treatment group imbalances. The plan should also identify any selected subgroups and state the type of analysis, for example, intention-to-treat or modified intention-to-treat, that will be conducted. The plan should be finalized before trial data are unblinded; publication of the analysis plan before the completion of the trial is a best practice.[148-150]*"[12]

In the current protocol, it is stated that "*a Statistical Analysis Plan (SAP) will be written and finalized before study closure, i.e., database closure. The SAP will provide full details of the analyses and data displays to be used for data derivations.*"[w]

The protocol currently includes a general description of the statistics that will be performed:[w]

- "*Descriptive statistics: descriptive statistics will be presented for each treatment of the low- and high risk paediatric CD groups and, where applicable, for the paired difference of each patient: mean, median, standard deviation, standard error, quartiles, minimum, maximum, and the two-sided 95% confidence limits of mean and median. Frequency tables will be presented where applicable.*

- *Primary analysis: Difference in the 12 months steroid/EEN free sustained remission rates between the treatment groups will be sought using Chi square test. Mantel Haenzel test will be used to combine data from all participating sites. The analysis will compare the two treatment arms of high and low risk groups independently.*

- *Secondary analyses: For each CD risk group, Chi-square tests or Fisher's exact tests (as appropriate) will be used to compare, between the two arms, the rate:…"* of e.g. remission at month 2, dropouts, serious adverse events, etc. (for full details we refer to the full protocol).

- "*For each CD risk group, Student's t tests or Wilcoxon rank sum tests (as appropriate) will be used to compare between the two arms: …*" e.g. the change in EQ-5D scores (forms EQ-5D-Y proxy1, EQ-5D-Y and EQ-5D-5L) between each visit and baseline, etc. (for full details we refer to the full protocol).

This information will thus be updated before study closure. The results from these statistics will be used in the economic model (see part 3.6).

3.6 ECONOMIC MODELLING

As mentioned in the introduction (see part 1.3), an economic evaluation is "*the comparative analysis of alternative courses of action in terms of both their costs and consequences*".[11] In economic evaluations, the incremental cost-effectiveness ratio (ICER) is calculated applying the following general formula:

- ICER = IC/IE = $(C_{Int} – C_{Comp})$ / $(E_{Int} – E_{Comp})$

  With C: costs; Comp: comparator; E: effects; IC: incremental cost; IE: incremental effect; Int: intervention.

Therefore, the initial task is to identify, measure and value the differences in costs and effects of the alternatives being considered. This report provides an overview of how information for these incremental elements will be gathered. Once this is done for the REDUCE-RISK trial, the relevant elements will be brought together in an economic model.

This trial-based economic evaluation will be performed according to the national guidelines for economic evaluations (e.g. for the choice of discount rate for costs and outcomes).[102] Following these guidelines, outcomes will be presented in the base case as extra costs per QALY gained. QALYs are calculated by combining the QoL scores (utilities) that are measured at regular intervals (see part 3.1) with data on survival.

The ISPOR guidelines also mention the following:

- "*Because economic outcomes in trials are the result of a single sample drawn from the population, one should report the variability in these outcomes that arises from such sampling. … One approach for reporting this variability is to construct a confidence interval for the cost-effectiveness ratio … or to construct an acceptability curve.*"[12]
- "*Uncertainty should be assessed for any parameter estimates that, when varied, have the potential to influence policy. Examples include unit costs and the discount rate. One approach to this assessment is sensitivity analysis.*"[12]

These guidelines will be followed by performing probabilistic sensitivity analysis and presenting both the cost-effectiveness plane (incl. confidence interval around the ICER), as well as the cost-effectiveness acceptability curve. One-way sensitivity analysis will be performed for influential variables like unit costs and discount rates. Furthermore, sensitivity analysis will also be performed for the utility value sets (applying different national value sets and VAS-scores – see part 3.1.6) as well as for extrapolation purposes beyond the trial follow-up period (if deemed appropriate). In case of the latter, cost-effectiveness ratios will be calculated at various time horizons.[12]

The ISPOR guidelines also state that "*there are several analysis features that should be common to all analyses of economic data derived from clinical trials:*

- *1. The intention-to-treat population should be used for the primary analysis.*
- *2. A common time horizon(s) should be used for accumulating costs and outcomes; a within-trial assessment of costs and outcomes should be conducted, even when also modelling or projecting beyond the time horizon of the trial.*
- *3. An assessment of uncertainty is necessary for each measure (standard errors or confidence intervals for point estimates; P values for hypothesis tests).*

- *4. A(common) real discount rate should be applied to future costs and, when used in a CEA, to future outcomes.*
- *5. If data for some subjects are missing and/or censored, the analytic approach should address this issue consistently in the various analyses affected by missing data.*"[12]

These guidelines will also be followed.

The REDUCE-RISK trial is an international trial. Reinhold et al.[151] mention that "*In practice, there are often pooled/split analyses in which, on the one hand, pooled effectiveness data of all participating countries are taken into consideration, whereas, on the other hand, only resource data of the country concerned are considered. This approach is a trade-off between a country-based assignment of resources and a high statistical power concerning the effectiveness data.[152]*" Similarly, effectiveness data will be based on the analysis of all patients included in the trial, while country-specific input will be used for treatment costs (see 3.3) and adverse events (see 3.4).

Following the ISPOR guidelines, reporting of the methods and results of the economic evaluation will be performed according to the CHEERS (Consolidated Health Economic Evaluation Reporting Standards) guidelines.[153, 154]

4 REFERENCES

[1] Zarin DA, Tse T, Williams RJ, Carr S. Trial Reporting in ClinicalTrials.gov - The Final Rule. N Engl J Med. 2016 Nov 17;375(20):1998-2004.
[2] Ruemmele FM, Veres G, Kolho KL, Griffiths A, Levine A, Escher JC, et al. Consensus guidelines of ECCO/ESPGHAN on the medical management of pediatric Crohn's disease. J Crohns Colitis. 2014 Oct;8(10):1179-207.
[3] Froslie KF, Jahnsen J, Moum BA, Vatn MH, Group I. Mucosal healing in inflammatory bowel disease: results from a Norwegian population-based cohort. Gastroenterology. 2007 Aug;133(2):412-22.
[4] D'Haens G, Baert F, van Assche G, Caenepeel P, Vergauwe P, Tuynman H, et al. Early combined immunosuppression or conventional management in patients with newly diagnosed Crohn's disease: an open randomised trial. Lancet. 2008 Feb 23;371(9613):660-7.
[5] Khanna R, Bressler B, Levesque BG, Zou G, Stitt LW, Greenberg GR, et al. Early combined immunosuppression for the management of Crohn's disease (REACT): a cluster randomised controlled trial. Lancet. 2015 Nov 7;386(10006):1825-34.
[6] Colombel JF, Sandborn WJ, Reinisch W, Mantzaris GJ, Kornbluth A, Rachmilewitz D, et al. Infliximab, azathioprine, or combination therapy for Crohn's disease. N Engl J Med. 2010 Apr 15;362(15):1383-95.
[7] Walters TD, Kim MO, Denson LA, Griffiths AM, Dubinsky M, Markowitz J, et al. Increased effectiveness of early therapy with anti-tumor necrosis factor-alpha vs an immunomodulator in children with Crohn's disease. Gastroenterology. 2014 Feb;146(2):383-91.
[8] Hyams J, Crandall W, Kugathasan S, Griffiths A, Olson A, Johanns J, et al. Induction and maintenance infliximab therapy for the treatment of moderate-to-severe Crohn's disease in children. Gastroenterology. 2007 Mar;132(3):863-73; quiz 1165-6.
[9] Hyams JS, Griffiths A, Markowitz J, Baldassano RN, Faubion WA, Jr., Colletti RB, et al. Safety and efficacy of adalimumab for moderate to severe Crohn's disease in children. Gastroenterology. 2012 Aug;143(2):365-74 e2.
[10] Sassi F, Archard L, Le Grand J. Equity and the economic evaluation of healthcare; 2001.
[11] Drummond M, Sculpher M, Torrance G, O'Brien B, Stoddart G. Methods for the Economic Evaluation of Health Care Programmes: Oxford University Press 2005.
[12] Ramsey SD, Willke RJ, Glick H, Reed SD, Augustovski F, Jonsson B, et al. Cost-effectiveness analysis alongside clinical trials II-An ISPOR Good Research Practices Task Force report. Value Health. 2015 Mar;18(2):161-72.
[13] Anderson R. Systematic reviews of economic evaluations: utility or futility? Health Econ. 2010 Mar;19(3):350-64.
[14] Lund H, Brunnhuber K, Juhl C, Robinson K, Leenaars M, Dorch BF, et al. Towards evidence based research. Bmj. 2016 Oct 21;355:i5440.
[15] Xie F, Blackhouse G, Assasi N, Gaebel K, Robertson D, Goeree R. Cost-utility analysis of infliximab and adalimumab for refractory ulcerative colitis. Cost Effectiveness and Resource Allocation. 2009 02 Mar 2011;7:20.
[16] Lofland JH, Mallow P, Rizzo J. Cost-per-remission analysis of infliximab compared to adalimumab among adults with moderate-to-severe ulcerative colitis. Journal of Medical Economics. 2013 19 Aug 2013;16(4):461-7.
[17] Toor K, Druyts E, Jansen JP, Thorlund K. Cost per remission and cost per response with infliximab, adalimumab, and golimumab for the treatment of moderately-to-severely active ulcerative colitis. Journal of Medical Economics. 2015 Jun;18(6):437-46.

[18] Liu Y, Wu EQ, Bensimon AG, Fan CP, Bao Y, Ganguli A, et al. Cost per responder associated with biologic therapies for crohn's disease, psoriasis, and rheumatoid arthritis. Advances in Therapy. 2012 19 Mar 2013;29(7):620-34.
[19] Yokomizo L, Limketkai B, Park KT. Cost-effectiveness of adalimumab, infliximab or vedolizumab as first-line biological therapy in moderate-to-severe ulcerative colitis. BMJ Open Gastroenterol. 2016;3(1):e000093.
[20] Saro C, Ceballos D, Munoz F, De la Coba C, Aguilar MD, Lazaro P, et al. Resources Utilization and Costs the Year Before and After Starting Treatment with Adalimumab in Crohn's Disease Patients. Inflammatory Bowel Diseases. 2015 Jul;21(7):1631-40.
[21] Archer R, Tappenden P, Ren S, Martyn-St James M, Harvey R, Basarir H, et al. Infliximab, adalimumab and golimumab for treating moderately to severely active ulcerative colitis after the failure of conventional therapy (including a review of TA140 and TA262): clinical effectiveness systematic review and economic model. Health Technol Assess. 2016 May;20(39):1-326.
[22] Tappenden P, Ren S, Archer R, Harvey R, James MM, Basarir H, et al. A Model-Based Economic Evaluation of Biologic and Non-Biologic Options for the Treatment of Adults with Moderately-to-Severely Active Ulcerative Colitis after the Failure of Conventional Therapy. Pharmacoeconomics. 2016 Oct;34(10):1023-38.
[23] Assasi N, Blackhouse G, Xie F, Gaebel K, Marshall J, Irvine E, et al. Anti-TNF-a drugs for refractory inflammatory bowel disease: clinical- and cost-effectiveness analyses. Report. Ottawa: Canadian Agency for Drugs and Technologies in Health (CADTH); 2009.
[24] Blackhouse G, Assasi N, Xie F, Marshall J, Irvine EJ, Gaebel K, et al. Canadian cost-utility analysis of initiation and maintenance treatment with anti-TNF-alpha drugs for refractory Crohn's disease. Journal of Crohn's and Colitis. 2012 21 Jul 2012;6(1):77-85.
[25] Canadian Agency for Drugs and Technologies in Health (CADTH). Common Drug Review Pharmacoeconomic Review Report for Simponi; November 2014.
[26] Dretzke J, Edlin R, Round J, Connock M, Hulme C, Czeczot J, et al. A systematic review and economic evaluation of the use of tumour necrosis factor-alpha (TNF-a) inhibitors, adalimumab and infliximab, for Crohn's disease. 2011;15(6):1-250.
[27] National Institute for Health and Care Excellence (NICE). Infliximab and adalimumab for the treatment of Crohn's disease's (TA187); May 2010.
[28] Essat M, Tappenden P, Ren S, Bessey A, Archer R, Wong R, et al. Vedolizumab for the treatment of adults with moderately to severely active ulcerative colitis: A Single Technology Appraisal; 2014.
[29] Essat M, Tappenden P, Ren S, Bessey A, Archer R, Wong R, et al. Vedolizumab for the Treatment of Adults with Moderate-to-Severe Active Ulcerative Colitis: An Evidence Review Group Perspective of a NICE Single Technology Appraisal. PharmacoEconomics. 2016;34(3):245-57.
[30] Rafia R, Scope A, Harnan S, Stevens JW, Stevenson M, Sutton A, et al. Vedolizumab for the treatment of adults with moderately to severely active Crohn’s disease: A Single Technology Appraisal; 2014.
[31] Bodger K, Kikuchi T, Hughes D. Cost-effectiveness of biological therapy for Crohn's disease: Markov cohort analyses incorporating United Kingdom patient-level cost data. Alimentary Pharmacology and Therapeutics. 2009 17 Mar 2010;30(3):265-74.
[32] Kaplan GG, Hur C, Korzenik J, Sands BE. Infliximab dose escalation vs initiation of adalimumab for loss of response in Crohn's disease: a cost-effectiveness analysis. Alimentary Pharmacology and Therapeutics. 2007 09 Aug 2008;26(11-12):1509-20.
[33] Loftus EV, Johnson SJ, Yu AP, Wu EQ, Chao J, Mulani PM. Cost-effectiveness of adalimumab for the maintenance of remission in patients with Crohn's disease. European Journal of Gastroenterology and Hepatology. 2009 20 Oct 2010;21(11):1302-9.
[34] Stawowczyk E, Kawalec P, Pilc A. Cost-utility analysis of 1-year treatment with adalimumab/standard care and standard care alone for ulcerative colitis in Poland. Eur J Clin Pharmacol. 2016((Stawowczyk E.) StatSoft Polska Sp. z o.o., Krakow, Poland):1-7.
[35] Tang DH, Armstrong EP, Lee JK. Cost-utility analysis of biologic treatments for moderate-to-severe Crohn's disease. Pharmacotherapy. 2012 Jun;32(6):515-26.

[36] Yu AP, Johnson S, Wang ST, Atanasov P, Tang J, Wu E, et al. Cost utility of adalimumab versus infliximab maintenance therapies in the United States for moderately to severely active Crohn's disease. PharmacoEconomics. 2009 02 Jun 2010;27(7):609-21.
[37] Best WR, Becktel JM, Singleton JW, Kern F, Jr. Development of a Crohn's disease activity index. National Cooperative Crohn's Disease Study. Gastroenterology. 1976 Mar;70(3):439-44.
[38] Sostegni R, Daperno M, Scaglione N, Lavagna A, Rocca R, Pera A. Review article: Crohn's disease: monitoring disease activity. Aliment Pharmacol Ther. 2003 Jun;17 Suppl 2:11-7.
[39] Colombel JF, Sandborn WJ, Rutgeerts P, Enns R, Hanauer SB, Panaccione R, et al. Adalimumab for maintenance of clinical response and remission in patients with Crohn's disease: the CHARM trial. Gastroenterology. 2007 Jan;132(1):52-65.
[40] Buchanan J, Wordsworth S, Ahmad T, Perrin A, Vermeire S, Sans M, et al. Managing the long term care of inflammatory bowel disease patients: The cost to European health care providers. Journal of Crohn's and Colitis. 2011;5(4):301-16.
[41] Sandborn WJ, van Assche G, Reinisch W, Colombel JF, D'Haens G, Wolf DC, et al. Adalimumab induces and maintains clinical remission in patients with moderate-to-severe ulcerative colitis. Gastroenterology. 2012 Feb;142(2):257-65 e1-3.
[42] Woehl A, Hawthorne A, McEwan P. The relation between disease activity, quality of life and health utility in patients with ulcerative colitis. Gut. 2008;57(Suppl. 1):A153.
[43] Swinburn P, Elwick H, Bean K, Curry A, Patel S, Bodger K, et al. The Impact of Surgery on Health Related Quality of Life in Ulcerative Colitis. *Gut Conference: Digestive Disorders Federation Meeting*. Liverpool 2012.
[44] MSD. Manufacturer Submission of Evidence: Infliximab (Remicade). Submission to NICE; 2014.
[45] MSD. Manufacturer Submission of Evidence: Golimumab (Simponi). Submission to NICE; 2014.
[46] Casellas F, Lopez Vivancos J, Malagela JR. Previous experience and quiality of life in patients with inflammatory bowel disease during relapse. Rev Esp Enferm Dig. 2003 Jul;95(7):476-9, 1-5.
[47] Leidl R, Reitmeir P, Konig HH, Stark R. The performance of a value set for the EQ-5D based on experienced health states in patients with inflammatory bowel disease. Value Health. 2012 Jan;15(1):151-7.
[48] Vaizey C, Gibson P, Black C, Nicholls R, Weston A, Gaya D, et al. The relationship between disease severity, quality of life and health care resource utilization among United Kingdom patients with ulcerative colitis. 8th Congress of ECCO; 14–16 February 2013; Vienna, Austria: Journal of Crohn's and Colitis Conference; 14–16 February 2013.
[49] van der Valk ME, Mangen MJ, Dijkstra G, van Bodegraven AA, Fidder H, de Jong DJ, et al. Is There a Difference in Quality of Life and Costs Between Ulcerative Colitis Patients with a Pouch or an Ileostomy? *Gastroenterology Conference: Digestive Disease Week*. San Diego, CA 19–22 May 2012.
[50] Richards DM, Hughes SA, Irving MH, Scott NA. Patient quality of life after successful restorative proctocolectomy is normal. Colorectal Dis. 2001 Jul;3(4):223-6.
[51] Kuruvilla K, Osler T, Hyman NH. A comparison of the quality of life of ulcerative colitis patients after IPAA vs ileostomy. Dis Colon Rectum. 2012 Nov;55(11):1131-7.
[52] Sandborn WJ, Feagan BG, Marano C, Zhang H, Strauss R, Johanns J, et al. Subcutaneous golimumab induces clinical response and remission in patients with moderate-to-severe ulcerative colitis. Gastroenterology. 2014 Jan;146(1):85-95; quiz e14-5.
[53] Rutgeerts P, Sandborn WJ, Feagan BG, Reinisch W, Olson A, Johanns J, et al. Infliximab for induction and maintenance therapy for ulcerative colitis. N Engl J Med. 2005 Dec 08;353(23):2462-76.
[54] Gregor JC, McDonald JW, Klar N, Wall R, Atkinson K, Lamba B, et al. An evaluation of utility measurement in Crohn's disease. Inflamm Bowel Dis. 1997 Winter;3(4):265-76.
[55] Buxton MJ, Lacey LA, Feagan BG, Niecko T, Miller DW, Townsend RJ. Mapping from disease-specific measures to utility: an analysis of the relationships between the

Inflammatory Bowel Disease Questionnaire and Crohn's Disease Activity Index in Crohn's disease and measures of utility. Value Health. 2007 May-Jun;10(3):214-20.
[56] Sandborn WJ, Feagan BG, Marano C, Zhang H, Strauss R, Johanns J, et al. Subcutaneous golimumab maintains clinical response in patients with moderate-to-severe ulcerative colitis. Gastroenterology. 2014 Jan;146(1):96-109 e1.
[57] Takeda Pharmaceuticals. Vedolizumab (Entyvio) for the treatment of adults with moderate to severe ulcerative colitis. Manufacturer's submission to the National Institute for Health and Care Excellence. High Wycombe: Takeda; 2014.
[58] Sandborn WJ, Feagan BG, Rutgeerts P, Hanauer S, Colombel JF, Sands BE, et al. Vedolizumab as induction and maintenance therapy for Crohn's disease. N Engl J Med. 2013 Aug 22;369(8):711-21.
[59] Sands BE, Feagan BG, Rutgeerts P, Colombel JF, Sandborn WJ, Sy R, et al. Effects of vedolizumab induction therapy for patients with Crohn's disease in whom tumor necrosis factor antagonist treatment failed. Gastroenterology. 2014 Sep;147(3):618-27 e3.
[60] Woehl A, Hawthorne B, Morgan C, Punekar Y, McEwan P. The epidemiology and healthcare resource use in patients with Crohn’s disease: a population based UK study. Value Health. 2007;10:A355.
[61] Sandborn WJ, Colombel JF, Enns R, Feagan BG, Hanauer SB, Lawrance IC, et al. Natalizumab induction and maintenance therapy for Crohn's disease. N Engl J Med. 2005 Nov 03;353(18):1912-25.
[62] Tsai HH, Punekar YS, Morris J, Fortun P. A model of the long-term cost effectiveness of scheduled maintenance treatment with infliximab for moderate-to-severe ulcerative colitis. Aliment Pharmacol Ther. 2008 Nov 15;28(10):1230-9.
[63] Arseneau KO, Sultan S, Provenzale DT, Onken J, Bickston SJ, Foley E, et al. Do patient preferences influence decisions on treatment for patients with steroid-refractory ulcerative colitis? Clin Gastroenterol Hepatol. 2006 Sep;4(9):1135-42.
[64] Hu D, Grossman D, Levin C, Blanchard K, Goldie SJ. Cost-effectiveness analysis of alternative first-trimester pregnancy termination strategies in Mexico City. BJOG. 2009 May;116(6):768-79.
[65] Smith KJ, Roberts MS. Quality-of-Life Utility Values for Erectile Function and Sildenafil Treatment. Clin Drug Investig. 2005;25(2):99-105.
[66] Punekar YS, Hawkins N. Cost-effectiveness of infliximab for the treatment of acute exacerbations of ulcerative colitis. Eur J Health Econ. 2010 Feb;11(1):67-76.
[67] Thompson JP, Noyes K, Dorsey ER, Schwid SR, Holloway RG. Quantitative risk-benefit analysis of natalizumab. Neurology. 2008 Jul 29;71(5):357-64.
[68] Reinisch W, Sandborn WJ, Hommes DW, D'Haens G, Hanauer S, Schreiber S, et al. Adalimumab for induction of clinical remission in moderately to severely active ulcerative colitis: results of a randomised controlled trial. Gut. 2011 Jun;60(6):780-7.
[69] AbbVie. Adalimumab, Golimumab and Infliximab, for the Treatment of Ulcerative Colitis (Subacute). Submission to NICE; 2014.
[70] Turner D, Walsh CM, Steinhart AH, Griffiths AM. Response to corticosteroids in severe ulcerative colitis: a systematic review of the literature and a meta-regression. Clin Gastroenterol Hepatol. 2007 Jan;5(1):103-10.
[71] Odes S, Vardi H, Friger M, Esser D, Wolters F, Moum B, et al. Clinical and economic outcomes in a population-based European cohort of 948 ulcerative colitis and Crohn's disease patients by Markov analysis. Aliment Pharmacol Ther. 2010 Apr;31(7):735-44.
[72] Kane S, Huo D, Aikens J, Hanauer S. Medication nonadherence and the outcomes of patients with quiescent ulcerative colitis. Am J Med. 2003 Jan;114(1):39-43.
[73] Targan SR, Hanauer SB, van Deventer SJ, Mayer L, Present DH, Braakman T, et al. A short-term study of chimeric monoclonal antibody cA2 to tumor necrosis factor alpha for Crohn's disease. Crohn's Disease cA2 Study Group. N Engl J Med. 1997 Oct 09;337(15):1029-35.
[74] Sandborn W, Lukas M, Rutgeerts P, Pollack P, Kent J. Fully human anti-TNF adalimumab maintains remission for one year in patients with active Crohn's disease: a randomised, controlled cohort [abstract]. Gut. 2006;55(Suppl 2):A27.

[75] Rutgeerts P, Feagan BG, Lichtenstein GR, Mayer LF, Schreiber S, Colombel JF, et al. Comparison of scheduled and episodic treatment strategies of infliximab in Crohn's disease. Gastroenterology. 2004 Feb;126(2):402-13.
[76] Hanauer SB, Feagan BG, Lichtenstein GR, Mayer LF, Schreiber S, Colombel JF, et al. Maintenance infliximab for Crohn's disease: the ACCENT I randomised trial. Lancet. 2002 May 04;359(9317):1541-9.
[77] Silverstein MD, Loftus EV, Sandborn WJ, Tremaine WJ, Feagan BG, Nietert PJ, et al. Clinical course and costs of care for Crohn's disease: Markov model analysis of a population-based cohort. Gastroenterology. 1999 Jul;117(1):49-57.
[78] Hanauer SB, Sandborn WJ, Rutgeerts P, Fedorak RN, Lukas M, MacIntosh D, et al. Human anti-tumor necrosis factor monoclonal antibody (adalimumab) in Crohn's disease: the CLASSIC-I trial. Gastroenterology. 2006 Feb;130(2):323-33; quiz 591.
[79] Feagan BG, Rutgeerts P, Sands BE, Hanauer S, Colombel JF, Sandborn WJ, et al. Vedolizumab as induction and maintenance therapy for ulcerative colitis. N Engl J Med. 2013 Aug 22;369(8):699-710.
[80] Suzuki Y, Motoya S, Hanai H, Matsumoto T, Hibi T, Robinson AM, et al. Efficacy and safety of adalimumab in Japanese patients with moderately to severely active ulcerative colitis. J Gastroenterol. 2014 Feb;49(2):283-94.
[81] Sandborn W, Rutgeerts P, Enns R, et al. Adalimumab rapidly induces clinical remission and response in patients with moderate to severe Crohn’s disease who had secondary failure to inflixmab therapy: results of the GAIN study. Am J Gastroenterol. 2006;101(S408):A1147.
[82] Colombel JF, Sandborn WJ, Rutgeerts P, Kamm MA, Yu AP, Wu EQ, et al. Comparison of two adalimumab treatment schedule strategies for moderate-to-severe Crohn's disease: results from the CHARM trial. Am J Gastroenterol. 2009 May;104(5):1170-9.
[83] Swenson BR, Hollenbeak CS, Koltun WA. Factors affecting cost and length of stay associated with the ileal pouch-anal anastomosis. Dis Colon Rectum. 2003 Jun;46(6):754-61.
[84] Johnson P, Richard C, Ravid A, Spencer L, Pinto E, Hanna M, et al. Female infertility after ileal pouch-anal anastomosis for ulcerative colitis. Dis Colon Rectum. 2004 Jul;47(7):1119-26.
[85] Krausz MM, Duek SD. Restorative proctocolectomy with ileal pouch-anal anastomosis for ulcerative colitis and familial adenomatous polyposis: twenty years follow-up in 174 patients. Isr Med Assoc J. 2005 Jan;7(1):23-7.
[86] Abdelrazeq AS, Kandiyil N, Botterill ID, Lund JN, Reynolds JR, Holdsworth PJ, et al. Predictors for acute and chronic pouchitis following restorative proctocolectomy for ulcerative colitis. Colorectal Dis. 2008 Oct;10(8):805-13.
[87] Roberts SE, Williams JG, Yeates D, Goldacre MJ. Mortality in patients with and without colectomy admitted to hospital for ulcerative colitis and Crohn's disease: record linkage studies. Bmj. 2007 Nov 17;335(7628):1033.
[88] Feagan BG, Panaccione R, Sandborn WJ, D'Haens GR, Schreiber S, Rutgeerts PJ, et al. Effects of adalimumab therapy on incidence of hospitalization and surgery in Crohn's disease: results from the CHARM study. Gastroenterology. 2008 Nov;135(5):1493-9.
[89] Lemann M. Review article: can post-operative recurrence in Crohn's disease be prevented? Aliment Pharmacol Ther. 2006 Oct;24 Suppl 3:22-8.
[90] McLeod RS, Wolff BG, Steinhart AH, Carryer PW, O'Rourke K, Andrews DF, et al. Prophylactic mesalamine treatment decreases postoperative recurrence of Crohn's disease. Gastroenterology. 1995 Aug;109(2):404-13.
[91] Brignola C, Cottone M, Pera A, Ardizzone S, Scribano ML, De Franchis R, et al. Mesalamine in the prevention of endoscopic recurrence after intestinal resection for Crohn's disease. Italian Cooperative Study Group. Gastroenterology. 1995 Feb;108(2):345-9.
[92] Caprilli R, Andreoli A, Capurso L, Corrao G, D'Albasio G, Gioieni A, et al. Oral mesalazine (5-aminosalicylic acid; Asacol) for the prevention of post-operative recurrence of

Crohn's disease. Gruppo Italiano per lo Studio del Colon e del Retto (GISC). Aliment Pharmacol Ther. 1994 Feb;8(1):35-43.
[93] Sutherland LR, Martin F, Bailey RJ, Fedorak RN, Poleski M, Dallaire C, et al. A randomized, placebo-controlled, double-blind trial of mesalamine in the maintenance of remission of Crohn's disease. The Canadian Mesalamine for Remission of Crohn's Disease Study Group. Gastroenterology. 1997 Apr;112(4):1069-77.
[94] Lochs H, Mayer M, Fleig WE, Mortensen PB, Bauer P, Genser D, et al. Prophylaxis of postoperative relapse in Crohn's disease with mesalamine: European Cooperative Crohn's Disease Study VI. Gastroenterology. 2000 Feb;118(2):264-73.
[95] Hanauer SB, Korelitz BI, Rutgeerts P, Peppercorn MA, Thisted RA, Cohen RD, et al. Postoperative maintenance of Crohn's disease remission with 6-mercaptopurine, mesalamine, or placebo: a 2-year trial. Gastroenterology. 2004 Sep;127(3):723-9.
[96] Lichtenstein GR, Yan S, Bala M, Blank M, Sands BE. Infliximab maintenance treatment reduces hospitalizations, surgeries, and procedures in fistulizing Crohn's disease. Gastroenterology. 2005 Apr;128(4):862-9.
[97] Frolkis AD, Dykeman J, Negron ME, Debruyn J, Jette N, Fiest KM, et al. Risk of surgery for inflammatory bowel diseases has decreased over time: a systematic review and meta-analysis of population-based studies. Gastroenterology. 2013 Nov;145(5):996-1006.
[98] Loftus EV, Jr., Delgado DJ, Friedman HS, Sandborn WJ. Colectomy and the incidence of postsurgical complications among ulcerative colitis patients with private health insurance in the United States. Am J Gastroenterol. 2008 Jul;103(7):1737-45.
[99] Mahadevan U, Loftus EV, Jr., Tremaine WJ, Pemberton JH, Harmsen WS, Schleck CD, et al. Azathioprine or 6-mercaptopurine before colectomy for ulcerative colitis is not associated with increased postoperative complications. Inflamm Bowel Dis. 2002 Sep;8(5):311-6.
[100] Feagan BG, Sandborn WJ, Lazar A, Thakkar RB, Huang B, Reilly N, et al. Adalimumab therapy is associated with reduced risk of hospitalization in patients with ulcerative colitis. Gastroenterology. 2014 Jan;146(1):110-8 e3.
[101] National Institute of Health and Clinical Excellence. Guide to the methods of technology appraisal. London: NICE; 2008.
[102] EUnetHTA. Methods for health economic evaluations - A guideline based on current practices in Europe: European network for Health Technology Assessment; May 2015.
[103] EUnetHTA. Endpoints used for relative effectiveness assessment: health-related quality of life and utility measures: European network for Health Technology Assessment; 2015.
[104] Yu AP, Cabanilla LA, Wu EQ, Mulani PM, Chao J. The costs of Crohn's disease in the United States and other Western countries: a systematic review. Curr Med Res Opin. 2008 Feb;24(2):319-28.
[105] Feagan BG. Review article: economic issues in Crohn's disease--assessing the effects of new treatments on health-related quality of life. Aliment Pharmacol Ther. 1999 Sep;13 Suppl 4:29-37; discussion 8.
[106] Otley A, Smith C, Nicholas D, Munk M, Avolio J, Sherman PM, et al. The IMPACT questionnaire: a valid measure of health-related quality of life in pediatric inflammatory bowel disease. J Pediatr Gastroenterol Nutr. 2002 Oct;35(4):557-63.
[107] Stark RG, Reitmeir P, Leidl R, Konig HH. Validity, reliability, and responsiveness of the EQ-5D in inflammatory bowel disease in Germany. Inflamm Bowel Dis. 2010 Jan;16(1):42-51.
[108] Konig HH, Ulshofer A, Gregor M, von Tirpitz C, Reinshagen M, Adler G, et al. Validation of the EuroQol questionnaire in patients with inflammatory bowel disease. Eur J Gastroenterol Hepatol. 2002 Nov;14(11):1205-15.
[109] Shearer D, Morshed S. Common generic measures of health related quality of life in injured patients. Injury. 2011 Mar;42(3):241-7.
[110] van Reenen M, Janssen B. EQ-5D-5L User Guide: Basic information on how to use the EQ-5D-5L instrument (Version 2.1); April 2015.

[111] EuroQol Group. EuroQol--a new facility for the measurement of health-related quality of life. Health Policy. 1990 Dec;16(3):199-208.
[112] Thorrington D, Eames K. Measuring Health Utilities in Children and Adolescents: A Systematic Review of the Literature. PLoS ONE. 2015;10(8):e0135672.
[113] Ungar WJ. Challenges in health state valuation in paediatric economic evaluation: are QALYs contraindicated? Pharmacoeconomics. 2011 Aug;29(8):641-52.
[114] De Civita M, Regier D, Alamgir AH, Anis AH, Fitzgerald MJ, Marra CA. Evaluating health-related quality-of-life studies in paediatric populations: some conceptual, methodological and developmental considerations and recent applications. Pharmacoeconomics. 2005;23(7):659-85.
[115] van Reenen M, Janssen B, Oppe M, Kreimeier S, Greiner W. EQ-5D-Y User Guide: Basic information on how to use the EQ-5D-Y instrument (Version 1.0); August 2014.
[116] Jardine J, Glinianaia SV, McConachie H, Embleton ND, Rankin J. Self-reported quality of life of young children with conditions from early infancy: a systematic review. Pediatrics. 2014 Oct;134(4):e1129-48.
[117] Davidson T, Levin LA. Is the societal approach wide enough to include relatives? Incorporating relatives' costs and effects in a cost-effectiveness analysis. Appl Health Econ Health Policy. 2010;8(1):25-35.
[118] Janssen MF, Pickard AS, Golicki D, Gudex C, Niewada M, Scalone L, et al. Measurement properties of the EQ-5D-5L compared to the EQ-5D-3L across eight patient groups: a multi-country study. Qual Life Res. 2013 Sep;22(7):1717-27.
[119] Herdman M, Gudex C, Lloyd A, Janssen M, Kind P, Parkin D, et al. Development and preliminary testing of the new five-level version of EQ-5D (EQ-5D-5L). Qual Life Res. 2011 Dec;20(10):1727-36.
[120] Schwenkglenks M, Matter-Walstra K. Is the EQ-5D suitable for use in oncology? An overview of the literature and recent developments. Expert Rev Pharmacoecon Outcomes Res. 2016;16(2):207-19.
[121] Goldsmith KA, Dyer MT, Buxton MJ, Sharples LD. Mapping of the EQ-5D index from clinical outcome measures and demographic variables in patients with coronary heart disease. Health Qual Life Outcomes. 2010 Jun 4;8:54.
[122] Olsen JA, Lamu AN, Cairns J. In search of a common currency: A comparison of seven EQ-5D-5L value sets. Health Econ. 2017 Oct 24.
[123] National Institute for Health and Care Excellence (NICE). Guide to the methods of technology appraisal 2013; April 2013.
[124] Devlin N, Shah K, Feng Y, Mulhern B, Van Hout B. Valuing health-related quality of life: an EQ-5D-5L value set for England: Office of Health Economics 2016.
[125] National Institute for Health and Care Excellence (NICE). Position statement on use of the EQ-5D-5L valuation set; August 2017.
[126] van Hout B, Janssen MF, Feng YS, Kohlmann T, Busschbach J, Golicki D, et al. Interim scoring for the EQ-5D-5L: mapping the EQ-5D-5L to EQ-5D-3L value sets. Value Health. 2012 Jul-Aug;15(5):708-15.
[127] Wu XY, Ohinmaa A, Veugelers PJ. Sociodemographic and neighbourhood determinants of health-related quality of life among grade-five students in Canada. Qual Life Res. 2010 Sep;19(7):969-76.
[128] Canaway AG, Frew EJ. Measuring preference-based quality of life in children aged 6-7 years: a comparison of the performance of the CHU-9D and EQ-5D-Y--the WAVES pilot study. Qual Life Res. 2013 Feb;22(1):173-83.
[129] Oluboyede Y, Tubeuf S, McCabe C. Measuring health outcomes of adolescents: report from a pilot study. Eur J Health Econ. 2013 Feb;14(1):11-9.
[130] Reilly MC, Zbrozek AS, Dukes EM. The validity and reproducibility of a work productivity and activity impairment instrument. Pharmacoeconomics. 1993 Nov;4(5):353-65.
[131] Lerner D, Amick BC, 3rd, Rogers WH, Malspeis S, Bungay K, Cynn D. The Work Limitations Questionnaire. Med Care. 2001 Jan;39(1):72-85.

[132] Prasad M, Wahlqvist P, Shikiar R, Shih YC. A review of self-report instruments measuring health-related work productivity: a patient-reported outcomes perspective. Pharmacoeconomics. 2004;22(4):225-44.
[133] Assa A, Ish-Tov A, Rinawi F, Shamir R. School Attendance in Children With Functional Abdominal Pain and Inflammatory Bowel Diseases. J Pediatr Gastroenterol Nutr. 2015 Nov;61(5):553-7.
[134] Almadani SB, Adler J, Browning J, Green EH, Helvie K, Rizk RS, et al. Effects of inflammatory bowel disease on students' adjustment to college. Clin Gastroenterol Hepatol. 2014 Dec;12(12):2055-62 e1.
[135] Mackner LM, Bickmeier RM, Crandall WV. Academic achievement, attendance, and school-related quality of life in pediatric inflammatory bowel disease. J Dev Behav Pediatr. 2012 Feb;33(2):106-11.
[136] Kassarnig V, Bjerre-Nielsen A, Mones E, Lehmann S, Lassen DD. Class attendance, peer similarity, and academic performance in a large field study. PLoS ONE. 2017;12(11):e0187078.
[137] Goossens ME, Rutten-van Molken MP, Vlaeyen JW, van der Linden SM. The cost diary: a method to measure direct and indirect costs in cost-effectiveness research. J Clin Epidemiol. 2000 Jul;53(7):688-95.
[138] Mattke S, Balakrishnan A, Bergamo G, Newberry SJ. A review of methods to measure health-related productivity loss. Am J Manag Care. 2007 Apr;13(4):211-7.
[139] Bouwmans C, Krol M, Severens H, Koopmanschap M, Brouwer W, Hakkaart-van Roijen L. The iMTA Productivity Cost Questionnaire: A Standardized Instrument for Measuring and Valuing Health-Related Productivity Losses. Value Health. 2015 Sep;18(6):753-8.
[140] Abma FI, van der Klink JJ, Bultmann U. The work role functioning questionnaire 2.0 (Dutch version): examination of its reliability, validity and responsiveness in the general working population. J Occup Rehabil. 2013 Mar;23(1):135-47.
[141] Reilly MC, Gerlier L, Brabant Y, Brown M. Validity, reliability, and responsiveness of the work productivity and activity impairment questionnaire in Crohn's disease. Clin Ther. 2008 Feb;30(2):393-404.
[142] Vergara M, Montserrat A, Casellas F, Maudsley M, Gallardo O, Ricart E, et al. Validation of the Spanish Work Productivity and Activity impairment questionnaire: Crohn's disease version. Eur J Gastroenterol Hepatol. 2009 Jul;21(7):809-15.
[143] Vergara M, Montserrat A, Casellas F, Villoria A, Suarez D, Maudsley M, et al. A new validation of the Spanish Work Productivity and Activity Impairment Questionnaire-Crohn's disease version. Value Health. 2011 Sep-Oct;14(6):859-61.
[144] Cleemput I, Neyt M, Van de Sande S, Thiry N. Belgian guidelines for economic evaluations and budget impact analyses: second edition. Brussels: Belgian Health Care Knowledge Centre(KCE); 2012.
[145] Glick H, Doshi J, Sonnad S, Polsky D. Economic Evaluation in Clinical Trials. (2nd ed.). New York: Oxford University Press 2015.
[146] Mack DR, Young R, Kaufman SS, Ramey L, Vanderhoof JA. Methotrexate in patients with Crohn's disease after 6-mercaptopurine. J Pediatr. 1998 May;132(5):830-5.
[147] Rosh JR. Methotrexate. In: Mamula P, Baldassano RN, Markowitz JE, editors. Pediatric inflammatory bowel disease 2007.
[148] Ramsey SD, Sullivan SD, Kaplan RM, Wood DE, Chiang YP, Wagner JL. Economic analysis of lung volume reduction surgery as part of the National Emphysema Treatment Trial. NETT Research Group. Ann Thorac Surg. 2001 Mar;71(3):995-1002.
[149] Sullivan SD, Liljas B, Buxton M, Lamm CJ, O'Byrne P, Tan WC, et al. Design and analytic considerations in determining the cost-effectiveness of early intervention in asthma from a multinational clinical trial. Control Clin Trials. 2001 Aug;22(4):420-37.
[150] Spertus JA, Tooley J, Jones P, Poston C, Mahoney E, Deedwania P, et al. Expanding the outcomes in clinical trials of heart failure: the quality of life and economic components of EPHESUS (EPlerenone's neuroHormonal Efficacy and SUrvival Study). Am Heart J. 2002 Apr;143(4):636-42.

[151] Reinhold T, Brüggenjürgen B, Schlander M, Rosenfeld S, Hessel F, Willich S. Economic analysis based on multinational studies: methods for adapting findings to national contexts. J Public Health. 2010;18:327-35.
[152] Reed SD, Anstrom KJ, Bakhai A, Briggs AH, Califf RM, Cohen DJ, et al. Conducting economic evaluations alongside multinational clinical trials: toward a research consensus. Am Heart J. 2005 Mar;149(3):434-43.
[153] Husereau D, Drummond M, Petrou S, Carswell C, Moher D, Greenberg D, et al. Consolidated Health Economic Evaluation Reporting Standards (CHEERS) statement. Value Health. 2013 Mar-Apr;16(2):e1-5.
[154] Husereau D, Drummond M, Petrou S, Carswell C, Moher D, Greenberg D, et al. Consolidated Health Economic Evaluation Reporting Standards (CHEERS)--explanation and elaboration: a report of the ISPOR Health Economic Evaluation Publication Guidelines Good Reporting Practices Task Force. Value Health. 2013 Mar-Apr;16(2):231-50.

5 APPENDICES

APPENDIX 1. SEARCH STRATEGY ECONOMIC EVALUATIONS

HTA AGENCIES

In February 2016, the websites of HTA institutes (Table 23) were searched using free text for the disease (‘inflammatory’, ‘Crohn’, and ‘ulcerative’).

Table 23: List of INAHTA member websites searched for HTA reports

Abbreviation	Institute	Country
AETS	Agencia de Evaluación de Tecnologias Sanitarias	Spain
AETSA	Andalusian Agency for Health Technology Assessment	Spain
AGENAS	The Agency for Regional Healthcare	Italy
AHRQ	Agency for Healthcare Research and Quality	USA
AHTA	Adelaide Health Technology Assessment	Australia
AHTAPol	Agency for Health Technology Assessment in Poland	Poland
AQuAS	Agència de Qualitat i Avaluació Sanitàries de Catalunya	Spain
ASERNIP-S	Australian Safety and Efficacy Register of New Interventional Procedures -Surgical	Australia
ASSR	Agenzia Sanitaria e Sociale Regionale (Regional Agency for Health and Social Care)	Italy
AVALIA-T	Galician Agency for Health Technology Assessment	Spain
CADTH	Canadian Agency for Drugs and Technologies in Health	Canada
CDE	Center for Drug Evaluation	Taiwan
CEDIT	Comité d’Évaluation et de Diffusion des Innovations Technologiques	France
CEM	Inspection générale de la sécurité sociale (IGSS), Cellule d'expertise médicale	Luxembourg
CENETEC	Centro Nacional de Excelencia Tecnológica en Salud Reforma	Mexico
CONITEC	National Committee for Technology Incorporation	Brazil
CMeRC	Department of Internal Medicine	South Africa
CRD	Centre for Reviews and Dissemination	United Kingdom
DAHTA @DIMDI	German Agency for HTA at the German Institute for Medical Documentation and Information	Germany
DECIT-	Secretaria de Ciência, Tecnologia e Insumos Estratégicos,	Brazil

Abbreviation	Institute	Country
CGATS	Departamento de Ciência e Tecnologia	
ETESA	Department of Quality and Patient Safety of the Ministry Health of Chile	Chile
FinOHTA	Finnish Office for Health Care Technology Assessment	Finland
G-ba	The German Health Care System and the Federal Joint Committee	Germany
GÖG	Gesundheit Österreich	Austria
HAD-MSP	Health Assessment Division, Ministry of Public Health	Uruguay
HAS	Haute Autorité de Santé	France
HCT-NHSRC	Division of Healthcare Technology, National Health Systems Resource Center	India
HealthPACT	Health Policy Advisory Committee on Technology	Australia
HIQA	Health Information and Quality Authority	Ireland
HIS	Healthcare Improvement Scotland	United Kingdom
HQO	Evidence Development and Standards Branch	Canada
HSAC	Health Services Assessment Collaboration	New Zealand
HTA-HSR/DHTA	HTA & Health Services Research	Denmark
IECS	Institute for Clinical Effectiveness and Health Policy	Argentina
IETS	Instituto de Evaluación Tecnológica en Salud	Colombia
IHE	Institute of Health Economics	Canada
INESSS	Institut national d'excellence en santé et en services sociaux	Canada
IQWiG	Institut für Qualität und Wirtschaftlichkeit im Gesundheitswesen	Germany
KCE	Belgian Federal Health Care Knowledge Centre	Belgium
LBI of HTA	Ludwig Boltzmann Institut für Health Technology Assessment	Austria
MaHTAS	Health Technology Assessment Section at Ministry of Health of Malaysia	Malaysia
MTU-SFOPH	Medical Technology Unit - Swiss Federal Office of Public Health	Switzerland
NECA	National Evidence-based healthcare Collaboration Agency	Korea
NHC	New Zealand National Health Committee	New Zealand
NHMRC CTC	NHMRC Clinical Trials Centre	Australia
NIHR	National Institute for Health Research	United Kingdom
NOKC	Norwegian Knowledge Centre for Health Services	Norway
OSTEBA	Basque Office for Health Technology Assessment	Spain
RCHD-CS	Ministry of Public Health of the Republic of Kazakhstan, Republican Centre for Health Development, Centre of	Kazakhstan

Abbreviation	Institute	Country
	Standardization, HTA department	
SBU	Swedish Council on Technology Assessment in Health Care	Sweden
UCEETS	The National Coordination Unit of Health Technology Assessment and Implementation	Argentina
UVT	HTA Unit in A. Gemelli University Hospital	Italy
VASPVT	State Health Care Accreditation Agency under the Ministry of Health of the Republic of Lithuania	Lithuania
ZIN	Zorginstituut Nederland	The Netherlands
ZonMw	The Medical and Health Research Council of The Netherlands	The Netherlands
**Selection of ex or non-member websites**		
CHE	Centre for Health Economics	United Kingdom
CMT	Center for Medical Technology Assessment	Sweden
EUnetHTA	European Network for HealthTechnology Assessment	Europe
NICE	National Institute for Health and Care Excellence	United Kingdom
PHARMAC	Pharmaceutical Management Agency	New Zealand

The following databases were searched in September 2016: Centre for Reviews and Dissemination (CRD) databases (NHS Economic Evaluation Database (NHS EED) and Health Technology Assessments (HTA)), Medline, and Embase. Table 24 up to Table 28 provide an overview of the applied search strategies.

Table 24: Search strategy and results for CRD HTA

**Date**	23 September 2016		
**Database**	Centre for Reviews and Dissemination (CRD) (www.crd.york.ac.uk/CRDWeb)		
**Date covered**	All		
**Search strategy**	1	MeSH DESCRIPTOR Inflammatory Bowel Diseases EXPLODE ALL TREES	452
	2	* IN HTA	16 565
	3	#1 AND #2	63
	4	(humira)	29
	5	(adalimumab)	241
	6	#4 OR #5	241
**Result**	7	#3 AND #6	**6 references**
**Note**	The Mesh term 'Inflammatory Bowel Diseases' includes both Mesh terms 'Colitis, Ulcerative' and 'Crohn Disease'.<br>There exists a Mesh descriptor for adalimumab (113		

	hits). However, we preferred to use the free text search (humira and adalimumab) which provides more hits.	

HTA: Health Technology Assessment.

Table 25: Search strategy and results for CRD NHS EED

**Date**	23 September 2016		
**Database**	Centre for Reviews and Dissemination (CRD) (www.crd.york.ac.uk/CRDWeb)		
**Date covered**	All		
**Search strategy**	1	MeSH DESCRIPTOR Inflammatory Bowel Diseases EXPLODE ALL TREES	452
	2	* IN NHSEED	17 613
	3	#1 AND #2	98
	4	(humira)	29
	5	(adalimumab)	241
	6	#4 OR #5	241
**Result**	7	#3 AND #6	**17 references**
**Note**	See remarks in Table 24.		

EED: Economic Evaluation Database.

Table 26: Search strategy and results for Medline (OVID) (part I)

**Date**	2 October 2016		
**Database**	Ovid MEDLINE(R) without Revisions1996 to September Week 3 2016		
**Date covered**	All		
**Search strategy**	1	economics/	6295
	2	exp "Costs and Cost Analysis"/	139 083
	3	"Value of Life"/ec [Economics]	234
	4	Economics, Dental/	202
	5	exp Economics, Hospital/	12 792
	6	Economics, Medical/	1854
	7	Economics, Nursing/	577
	8	Economics, Pharmaceutical/	2280
	9	1 or 2 or 3 or 4 or 5 or 6 or 7 or 8	151 644
	10	(econom$ or cost$ or pric$ or pharmacoeconomic$).tw.	426 458
	11	(expenditure$ not energy).tw.	14 846
	12	(value adj1 money).tw.	17
	13	budget$.tw.	13 924
	14	10 or 11 or 12 or 13	440 535
	15	9 or 14	496 823

	16	letter.pt.	591 118
	17	editorial.pt.	307 061
	18	historical article.pt.	149 210
	19	16 or 17 or 18	1 034 647
	20	15 not 19	473 602
	21	Animals/	3 169 772
	22	human/	9 037 446
	23	21 not (21 and 22)	2 041 038
	24	20 not 23	429 892
	25	(metabolic adj cost).ti,ab,sh.	711
	26	((energy or oxygen) adj cost).ti,ab,sh.	1864
	27	24 not (25 or 26)	427 898
	28	adalimumab.mp.	4531
	29	humira.mp.	125
	30	28 or 29	4545
**Result**	31	27 and 30	**351 references**
**Note**	The MeSH term ‘exp Inflammatory Bowel Diseases/’ was linked to 42 130 hits. Adding this term with the boolean ‘and’ resulted in only 68 hits. Therefore, it was preferred not to include this term in the search and go through all 351 identified references.		

Table 27: Search strategy and results for Medline (OVID) (part II)

**Date**	2 October 2016		
**Database**	Ovid MEDLINE(R) In-Process & Other Non-Indexed Citations and Ovid MEDLINE(R) 1946 to Present		
**Date covered**	All		
**Search strategy**	1	cost$.mp.	531 586
	2	economic$.mp.	254 508
	3	budget$.mp.	28 776
	4	expenditure$.mp.	56 772
	5	1 or 2 or 3 or 4	766 578
	6	(adalimumab or humira).mp.	5384
	7	5 and 6	471
	8	crohn$.mp.	46217
	9	"inflammatory bowel disease$".mp.	38029
	10	15 or 17	68762
**Result**	11	7 and 19	**95 references**
**Note**	/		

Table 28: Search strategy and results for EMBASE

**Date**	2 October 2016		
**Database**	EMBASE		
**Date covered**	All		
**Search strategy**	1	socioeconomics'/exp	209 108
	2	cost benefit analysis'/exp	71 294
	3	cost effectiveness analysis'/exp	117 494
	4	cost of illness'/exp	15 945
	5	cost control'/exp	56 509
	6	economic aspect'/exp	1 294 654
	7	financial management'/exp	349 152
	8	health care cost'/exp	236 632
	9	health care financing'/exp	12 068
	10	health economics'/exp	701 828
	11	hospital cost'/exp	30 174
	12	finance'/exp OR 'funding'/exp OR fiscal OR financial	217 369
	13	cost minimization analysis'/exp	2848
	14	cost*:de,cl,ab,ti	795 164
	15	estimate*:de,cl,ab,ti	867 876
	16	variable*:de,cl,ab,ti	815 806
	17	unit:de,cl,ab,ti	502 013
	18	#14' NEAR/1 '#15' OR '#15' NEAR/1 '#14'	102 388
	19	#14' NEAR/1 '#16' OR '#16' NEAR/1 '#14'	252 974
	20	#14' NEAR/1 '#17' OR '#17' NEAR/1 '#14'	50 081
	21	#1 OR #2 OR #3 OR #4 OR #5 OR #6 OR #7 OR #8 OR #9 OR #10 OR #11 OR #12 OR #13 OR #18 OR #19 OR #20	1 719 993
	22	'inflammatory bowel disease'/exp	111 756
	23	'adalimumab'/exp	21 104
	24	humira:ab,ti	374
	25	#23 OR #24	21 120
	26	#22 AND #25	6439
**Result**	27	#21 AND #26	**782 references**
**Note**	/		

After removal of 153 duplicates, a total of 1098 references were identified (Table 29).

Table 29: Results of search strategy

Database	References
CRD HTA	6
CRD NHS EED	17
Medline	351
Medline In-Process & Other	95
Embase	782
**Total (incl. duplicates)**	**1251 references**
Duplicates	153
**Total (excl. duplicates)**	**1098 references**

APPENDIX 2. DATA EXTRACTION SHEET

Table 30: Data extraction sheet

	Elements to be extracted from the original economic evaluation
1	Reference (including all authors)
2	Conflict of interest and/or study funding
3	Country
4	Study question
5	Type of analysis (analytic technique) - e.g. cost-effectiveness analysis, cost-utility analysis, …
6	Design - e.g. Markov model, decision tree, …
7	Population
8	Intervention
9	Comparator
10	Time horizon
11	Discount rate for costs and/or effects
12	Perspective
13	Costs:<br>Cost items included; Measurement of resource use; Valuation of resource use; Data sources; Currency and cost year; Other aspects…
14	Outcomes:<br>Endpoints taken into account and/or health states; Valuation of health states; Treatment effect and Extrapolation; Utility assessment (Quality of Life); Data sources for outcomes; Other aspects…
15	Uncertainty - Scenario analysis; Sensitivity analysis
16	Assumptions
17	Results:<br>Cost-effectiveness and/or cost-utility (base case); Scenario analysis; Sensitivity analysis; Other aspects…
18	Conclusions:<br>The conclusion of the authors (which can be discussed in the actual critical assessment)
19	Remarks:<br>E.g. limitations of the study.

APPENDIX 3. EQ-5D QUESTIONNAIRES – SAMPLE VERSIONS

On the following pages, a sample version of the EQ-5D questionnaires is included:[z]

- 1) the English (UK) EQ-5D-5L questionnaire (paper self-complete version)
- 2) the English (UK) EQ-5D-Y questionnaire (paper self-complete version)
- 3) the English (UK) EQ-5D-Y Proxy1 questionnaire (paper self-complete version)

EQ-5D-5L: SAMPLE VERSION

This sample version is added on the following pages of the pdf-version of this report.

[z] © 2008 EuroQol Group. EQ-5D™ is a trade mark of the EuroQol Group.

Health Questionnaire

English version for the UK

Sample

UK (English) © 2009 EuroQol Group EQ-5D™ is a trade mark of the EuroQol Group

Under each heading, please tick the ONE box that best describes your health TODAY.

MOBILITY

I have no problems in walking about ❑

I have slight problems in walking about ❑

I have moderate problems in walking about ❑

I have severe problems in walking about ❑

I am unable to walk about ❑

SELF-CARE

I have no problems washing or dressing myself ❑

I have slight problems washing or dressing myself ❑

I have moderate problems washing or dressing myself ❑

I have severe problems washing or dressing myself ❑

I am unable to wash or dress myself ❑

USUAL ACTIVITIES *(e.g. work, study, housework, family or leisure activities)*

I have no problems doing my usual activities ❑

I have slight problems doing my usual activities ❑

I have moderate problems doing my usual activities ❑

I have severe problems doing my usual activities ❑

I am unable to do my usual activities ❑

PAIN / DISCOMFORT

I have no pain or discomfort ❑

I have slight pain or discomfort ❑

I have moderate pain or discomfort ❑

I have severe pain or discomfort ❑

I have extreme pain or discomfort ❑

ANXIETY / DEPRESSION

I am not anxious or depressed ❑

I am slightly anxious or depressed ❑

I am moderately anxious or depressed ❑

I am severely anxious or depressed ❑

I am extremely anxious or depressed ❑

Sample

UK (English) © 2009 EuroQol Group EQ-5D™ is a trade mark of the EuroQol Group

- We would like to know how good or bad your health is TODAY.
- This scale is numbered from 0 to 100.
- 100 means the best health you can imagine.
  0 means the worst health you can imagine.
- Mark an X on the scale to indicate how your health is TODAY.
- Now, please write the number you marked on the scale in the box below.

YOUR HEALTH TODAY = ☐

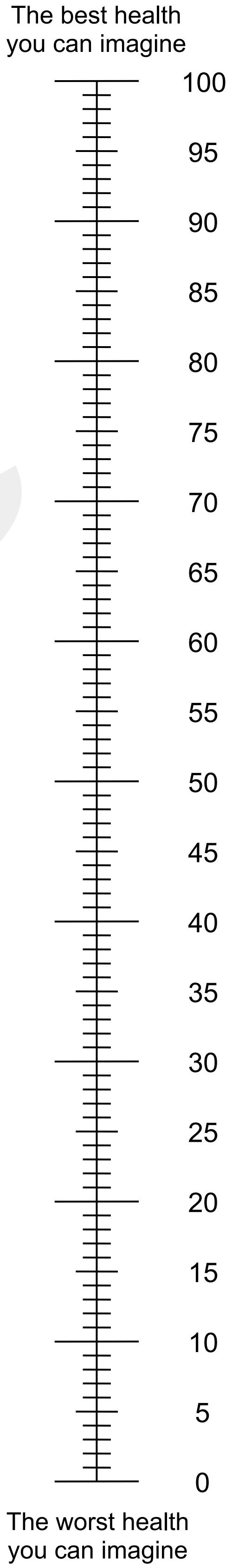

Sample

UK (English) © 2009 EuroQol Group EQ-5D™ is a trade mark of the EuroQol Group

EQ-5D-Y: SAMPLE VERSION

This sample version is added on the following pages of the pdf-version of this report.

Health Questionnaire

English version for the UK

Sample

UK (English) © 2008 EuroQol Group EQ-5D™ is a trade mark of the EuroQol Group

EQ-5D-Y

Describing your health TODAY

Under each heading, please tick the ONE box that best describes your health TODAY.

Mobility *(walking about)*

I have no problems walking about ❑

I have some problems walking about ❑

I have a lot of problems walking about ❑

Looking after myself

I have no problems washing or dressing myself ❑

I have some problems washing or dressing myself ❑

I have a lot of problems washing or dressing myself ❑

Doing usual activities *(for example, going to school, hobbies, sports, playing, doing things with family or friends)*

I have no problems doing my usual activities ❑

I have some problems doing my usual activities ❑

I have a lot of problems doing my usual activities ❑

Having pain or discomfort

I have no pain or discomfort ❑

I have some pain or discomfort ❑

I have a lot of pain or discomfort ❑

Feeling worried, sad or unhappy

I am not worried, sad or unhappy ❑

I am a bit worried, sad or unhappy ❑

I am very worried, sad or unhappy ❑

Sample

UK (English) © 2008 EuroQol Group EQ-5D™ is a trade mark of the EuroQol Group

How good is your health TODAY

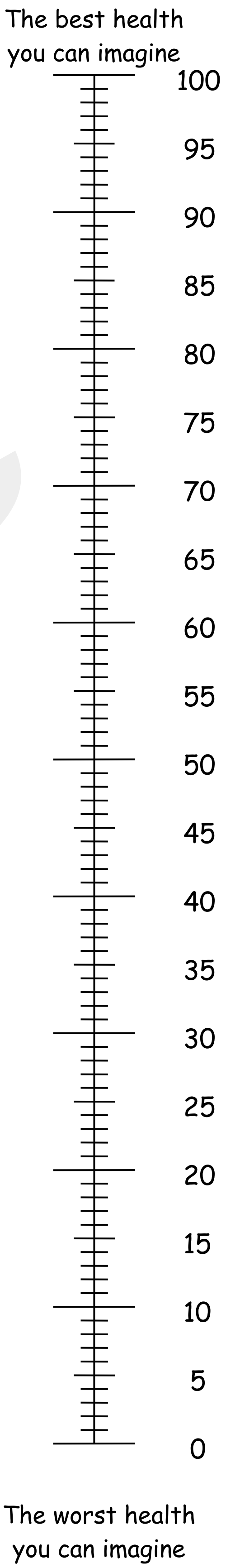

- We would like to know how good or bad your health is TODAY.
- This line is numbered from 0 to 100.
- 100 means the <u>best</u> health you can imagine.
  0 means the <u>worst</u> health you can imagine.
- Please mark an X on the line that shows how good or bad your health is TODAY.

Sample

UK (English) © 2008 EuroQol Group EQ-5D™ is a trade mark of the EuroQol Group

EQ-5D-Y PROXY1: SAMPLE VERSION

This sample version is added on the following pages of the pdf-version of this report.

Health Questionnaire

English version for the UK

Proxy version of the EQ-5D-Y: 1

(The purpose of this questionnaire is to explore how a care-giver or someone who knows the child well (proxy), would rate the health of the child. The proxy should not answer on behalf of the child, but rather rate the child's health as the proxy sees it)

Sample

UK (English) © 2014 EuroQol Group. EQ-5D™ is a trade mark of the EuroQol Group

EQ-5D-Y

Describing the child's health TODAY

Under each heading, please tick the ONE box that **you think** best describes the child's health **TODAY**.

Mobility *(walking about)*

He/she has no problems walking about ☐

He/she has some problems walking about ☐

He/she has a lot of problems walking about ☐

Looking after him/herself

He/she has no problems washing or dressing him/herself ☐

He/she has some problems washing or dressing him/herself ☐

He/she has a lot of problems washing or dressing him/herself ☐

Doing usual activities (*for example: going to school, hobbies, sports, playing, doing things with family or friends)*

He/she has no problems doing his/her usual activities ☐

He/she has some problems doing his/her usual activities ☐

He/she has a lot of problems doing his/her usual activities ☐

Having pain or discomfort

He/she has no pain or discomfort ☐

He/she has some pain or discomfort ☐

He/she has a lot of pain or discomfort ☐

Feeling worried, sad or unhappy

He/she is not worried, sad or unhappy ☐

He/she is a bit worried, sad or unhappy ☐

He/she is very worried, sad or unhappy ☐

Sample

UK (English) © 2014 EuroQol Group. EQ-5D™ is a trade mark of the EuroQol Group

The child's health TODAY

- We would like to know how good or bad you think the child's health is TODAY.
- This line is numbered 0 to 100.
- 100 means the <u>best</u> health you can imagine.
  0 means the <u>worst</u> health you can imagine.
- Please mark an X on the line to show how good or bad **you think** the child's health is **TODAY**.

The best health you can imagine

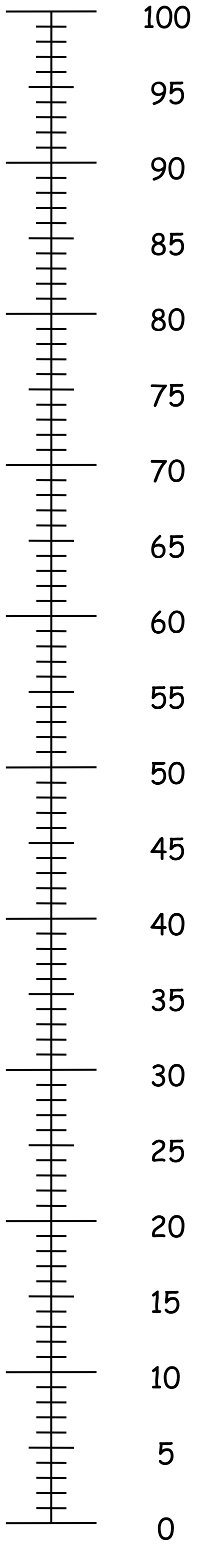

The worst health you can imagine

Sample

UK (English) © 2014 EuroQol Group. EQ-5D™ is a trade mark of the EuroQol Group

APPENDIX 4. DE NOVO SCHOOL ATTENDANCE QUESTIONNAIRE

On the following pages, the 'de novo' school attendance questionnaires is included:

- 1) the version that is provided at the start of the research
- 2) the version that is provided during follow-up visits

START OF RESEARCH (FINAL VERSION)

This sample version is added on the following pages of the pdf-version of this report.

QUESTIONNAIRE:

IMPACT ON CHILDREN's SCHOOL ATTENDANCE

(To be filled out at the **start of the research**)

Dear parents,

In this study, we would like to try to measure the impact of the health of your child on the **school attendance** of your child.

To do this, we would like to ask you a couple of questions at the start of the research and ask you to provide us with some feedback at each follow-up visit.

Your answers will only be used by the researchers involved in this study and the information collected will then be anonymised.

Your help with this research is really appreciated!

General questions:

Question 1: What is the patient code of the child who is participating in the study?

……………………………………………………………………………………

Question 2: On which date are you filling out this questionnaire?

Day	Month	Year
☐☐	☐☐	☐☐☐☐

Here are the questions to be filled out by the parents AT THE START OF THE RESEARCH relating to SCHOOL ATTENDANCE.

Question 3: In which month and which year was your child born?

Month ☐☐ Year ☐☐☐☐

Question 4: Can you give an image of a typical school week? (You can also report half days)

a) How many days does your child go to school in a typical week? (exclude home education)

…… days

b) Does your child have home schooling (or home education)?

☐ Yes ☐ No

c) If yes, how many hours per week does your child get home schooling for?

On average …… hours per week

d) If yes, is following home schooling a consequence of inflammatory bowel disease?

☐ Yes ☐ No

Question 5: Please can you collect some information in a diary (or something similar) to share with us at the next follow-up visit?

At the follow-up visits, we would like you to let us know how many days your child could not go to school. Therefore, **we would like you to keep a simple diary**, for the period between this visit and your next follow-up visit, with three items:

a1) Count the number of school days your child could attend.

b1) Count the number of school days your child could not attend.

c1) Count how many of these days you think your child could not attend due to the inflammatory bowel disease.

In case your child also follows home schooling (or home education), you can gather the same information:

a2) Count the number of home school days your child could attend.

b2) Count the number of home school days your child could not attend.

c2) Count how many of these days you think your child could not attend due to the inflammatory bowel disease.

If it is a half day, please count 0.5 days.

The answers for these three items can be provided at every follow-up visit. You can find a copy of these questions on the last page to take home with you. Please restart counting from zero after every follow-up visit where you provided this information in the questionnaire.

INFORMATION FOR PARENTS **<u>TO TAKE HOME</u>**
TO HELP IN COLLECTING INFORMATION
FOR THE NEXT FOLLOW-UP VISIT

Dear parents,

In this questionnaire, we asked you to gather information related to your child's school attendance. **<u>We would like you to keep a simple diary</u>** (or something similar) to gather this information. **<u>You can take this page with you to help you to remind you which information can be gathered</u>**.

<u>School attendance</u>:

a1) Count the number of school days your child could attend.

b1) Count the number of school days your child could not attend.

c1) Count how many of these days <u>you think</u> your child could not attend due to the inflammatory bowel disease.

In case your child also follows home schooling (or home education), you can gather the same information:

a2) Count the number of home school days your child could attend.

b2) Count the number of home school days your child could not attend.

c2) Count how many of these days <u>you think</u> your child could not attend due to the inflammatory bowel disease.

If it is a half day, please count 0.5 days. The answers for these three items can be provided at every follow-up visit. Please restart counting from zero after every follow-up visit where you provided this information in the questionnaire.

Thank you very much for your help with this research!

FOLLOW-UP VISITS (FINAL VERSION)

This sample version is added on the following pages of the pdf-version of this report.

QUESTIONNAIRE:

IMPACT ON CHILDREN's SCHOOL ATTENDANCE

(To be filled out at the **follow-up visits**)

Dear parents,

In this study, we would like to try to measure the impact of the health of your child on the **school attendance** of your child.

To do this, we would like to ask you a couple of questions at the start of the research and ask you to provide us with some feedback at every follow-up visit.

Your answers will only be used by the researchers involved in this study and the information collected will then be anonymised.

Your help in this research is really appreciated!

General questions:

Question 1: What is the patient code of the child who is participating in the study?

……………………………………………………………………………………

Question 2: On which date are you filling out this questionnaire?

Day	Month	Year
☐☐	☐☐	☐☐☐☐

Here are the questions to be filled out by the parents AT THE FOLLOW-UP VISIT related to SCHOOL ATTENDANCE.

Question 3: Has the school situation of your child changed since the completion of the previous questionnaire? (If yes, go to question 4. If no, go to question 5)

☐ Yes ☐ No

Question 4: Can you give an image of a typical school week? (You can also report half days)

a) How many days does your child go to school in a typical week? (exclude home education)

…… days

b) Does your child follow home schooling (or home education)?

☐ Yes ☐ No

c) If yes, how many hours per week does your child follow home schooling?

On average …… hours per week

d) If yes, is following home schooling a consequence of the inflammatory bowel disease?

☐ Yes ☐ No

Please, continue with the questionnaire on the next page.

Question 5: Can you answer the following questions based on the information collected in your diary?

In the period between the previous time you filled out this questionnaire and this follow-up visit:

a1) How many school days could your child attend during this period?

…… days

b1) How many school days could your child not attend?

…… days

c1) How many of these days (in answer b1) do you think your child could not attend due to the inflammatory bowel disease?

…… days

In case your child is also home schooled:

a2) How many home schooling days could your child attend during this period?

…… days

b2) How many home schooling days could your child not attend?

…… days

c2) How many of these days (in answer b2) do you think your child could not attend due to the inflammatory bowel disease?

…… days

In the next follow-up visit, we will gather the same information based on your diary. You can find a copy of these questions on the last page to take home with you. Please restart counting from zero after you provided this information during this follow-up visit.

INFORMATION FOR PARENTS **<u>TO TAKE HOME</u>**
TO HELP IN COLLECTING INFORMATION
FOR THE NEXT FOLLOW-UP VISIT

Dear parents,

In this questionnaire, we asked you to gather information related to your child's school attendance. **<u>We would like you to keep a simple diary</u>** (or something similar) to gather this information. **<u>You can take this page with you to help you to remind you which information can be gathered</u>**.

<u>School attendance</u>:

a1) Count the number of school days your child could attend.

b1) Count the number of school days your child could not attend.

c1) Count how many of these days <u>you think</u> your child could not attend due to the inflammatory bowel disease.

In case your child also follows home schooling (or home education), you can gather the same information:

a2) Count the number of home school days your child could attend.

b2) Count the number of home school days your child could not attend.

c2) Count how many of these days <u>you think</u> your child could not attend due to the inflammatory bowel disease.

If it is a half day, please count 0.5 days. The answers for these three items can be provided at every follow-up visit. Please restart counting from zero after every follow-up visit where you provided this information in the questionnaire.

Thank you very much for your help with this research!

APPENDIX 5. ABANDONED PRODUCTIVITY QUESTIONNAIRE

As described in part 3.2.2, we initially set up a de novo ‘school attendance and productivity questionnaire’. However, due to the identification of an existing questionnaire for productivity of parents with CD children, we decided to delete the productivity part of the questionnaire and include the WPAI:CD-CG questionnaire. Hereafter, we present the deleted parts, both for the version that is provided at the start of the research, as well as the version that is provided during follow-up visits.

START OF RESEARCH (IDENTIFICATION OF DELETED PARTS)

This sample version is added on the following pages of the pdf-version of this report.

QUESTIONNAIRE:

IMPACT ON CHILDREN's SCHOOL ATTENDANCE ~~AND PARENTS' PRODUCTIVITY~~

(To be filled out at the **start of the research**)

Dear parents,

In this study, we would like to try to measure the impact of the health of your child on ~~1)~~ the **school attendance** of your child ~~and 2) the **productivity** of the parents~~.

To do this, we would like to ask you a couple of questions at the start of the research and ask you to provide us with some feedback at each follow-up visit.

Your answers will only be used by the researchers involved in this study and the information collected will then be anonymised.

Your help with this research is really appreciated!

General questions:

Question 1: What is the patient code of the child who is participating in the study?

……………………………………………………………………………………

Question 2: On which date are you filling out this questionnaire?

Day		Month		Year			

Here are the questions to be filled out by the parents AT THE START OF THE RESEARCH relating to SCHOOL ATTENDANCE.

Question 3: In which month and which year was your child born?

Month ☐☐ Year ☐☐☐☐

Question 4: Can you give an image of a typical school week? (You can also report half days)

a) How many days does your child go to school in a typical week? (exclude home education)

…… days

b) Does your child have home schooling (or home education)?

☐ Yes ☐ No

c) If yes, how many hours per week does your child get home schooling for?

On average …… hours per week

d) If yes, is following home schooling a consequence of inflammatory bowel disease?

☐ Yes ☐ No

Question 5: Please can you collect some information in a diary (or something similar) to share with us at the next follow-up visit?

At the follow-up visits, we would like you to let us know how many days your child could not go to school. Therefore, **we would like you to keep a simple diary**, for the period between this visit and your next follow-up visit, with three items:

a1) Count the number of school days your child could attend.

b1) Count the number of school days your child could not attend.

c1) Count how many of these days you think your child could not attend due to the inflammatory bowel disease.

In case your child also follows home schooling (or home education), you can gather the same information:

a2) Count the number of home school days your child could attend.

b2) Count the number of home school days your child could not attend.

c2) Count how many of these days you think your child could not attend due to the inflammatory bowel disease.

If it is a half day, please count 0.5 days.

The answers for these three items can be provided at every follow-up visit. You can find a copy of these questions on the last page to take home with you. Please restart counting from zero after every follow-up visit where you provided this information in the questionnaire.

~~Please continue on the next page asking questions related to productivity.~~

Here are the questions to be filled out by the parents AT THE START OF THE RESEARCH related to parents' work (PRODUCTIVITY).

If there is one parent, please fill out the boxes next to "parent 1". If there are two parents, please fill out both boxes next to "parent 1" and "parent 2".

Question 6: In which month and which year were you born?

Parent 1: Month Year

Parent 2: Month Year

Question 7: What is your gender?

Parent 1: ☐ Male ☐ Female

Parent 2: ☐ Male ☐ Female

Question 8: Are you currently in paid work? (If no, please go to question 11)

Parent 1: ☐ Yes ☐ No

Parent 2: ☐ Yes ☐ No

Question 9: How many hours per week do you usually work? (Only count the hours for which you are paid)

Parent 1: ……. hours

Parent 2: ……. hours

Question 10: How many days per week do you usually work? (Only count the days for which you are paid)

Parent 1: ……. days

Parent 2: ……. days

~~**Question 11a**: Has your work situation changed recently due to the inflammatory bowel disease of your child? (If no, go to question 12)~~

~~Parent 1:~~ ☐ ~~Yes~~ ☐ ~~No~~

~~Parent 2:~~ ☐ ~~Yes~~ ☐ ~~No~~

~~**Question 11b**: If yes, how has your work situation changed? (A combination of the following answers is possible)~~

~~Parent 1:~~ ☐ ~~Working fewer days~~
☐ ~~More working from home~~
☐ ~~Others: ……………………………………………………………………~~
~~……………………………………………………………………………~~

~~Parent 2:~~ ☐ ~~Working fewer days~~
☐ ~~More working from home~~
☐ ~~Others: ……………………………………………………………………~~
~~……………………………………………………………………………~~

~~**Question 12a**: Have you asked for help (from family, friends, household help, etc.) due to the inflammatory bowel disease of your child?~~

☐ ~~Yes~~ ☐ ~~No~~

~~**Question 12b**: If yes, on average, how many hours per week do you get help related to the inflammatory bowel disease of your child?~~

~~On average …… hours per week~~

Question 13: Please can you gather some information in a diary (or something similar) to share with us at the next follow-up visit?

At the follow-up visits, we would like you to let us know how many days you could not do your work. Therefore, **we would like you to keep a simple diary**, for the period between this visit and your next follow-up visit, with the following five items (for both parents):

- For paid work:

  a) Count the number of paid working days that you could do your work (exclude holidays).

  b) Count the number of days you could not do your paid work.

  c) Count how many of these days you think you could not do your paid work related to the inflammatory bowel disease of your child.

- For unpaid work (housekeeping, volunteer work, shopping, gardening, etc.):

  d) Count the number of days you could not do your unpaid work.

  e) Count how many of these days you think you could not do your unpaid work related to the inflammatory bowel disease of your child.

If it is a half day, please count 0.5 days.

It would be really helpful if the answers for these five items could be provided at every follow-up visit. You can find a copy of these questions on the last page to take home with you. Please restart counting from zero after every follow-up visit where you provided this information in the questionnaire.

This is the end of this questionnaire.

Thank you very much for your help with this research!

INFORMATION FOR PARENTS **<u>TO TAKE HOME</u>**
TO HELP IN COLLECTING INFORMATION
FOR THE NEXT FOLLOW-UP VISIT

Dear parents,

In this questionnaire, we asked you to gather information related to your child's school attendance~~ and your work (productivity)~~. **<u>We would like you to keep a simple diary</u>** (or something similar) to gather this information. **<u>You can take this page ~~and the next one~~ with you to help you to remind you which information can be gathered</u>**.

<u>School attendance</u>:

a1) Count the number of school days your child could attend.

b1) Count the number of school days your child could not attend.

c1) Count how many of these days <u>you think</u> your child could not attend due to the inflammatory bowel disease.

In case your child also follows home schooling (or home education), you can gather the same information:

a2) Count the number of home school days your child could attend.

b2) Count the number of home school days your child could not attend.

c2) Count how many of these days <u>you think</u> your child could not attend due to the inflammatory bowel disease.

If it is a half day, please count 0.5 days. The answers for these three items can be provided at every follow-up visit. Please restart counting from zero after every follow-up visit where you provided this information in the questionnaire.

~~INFORMATION FOR PARENTS **TO TAKE HOME**~~
~~TO HELP IN COLLECTING INFORMATION~~
~~FOR THE NEXT FOLLOW-UP VISIT~~

~~**Productivity**: for both parents~~

- ~~For paid work:~~

  ~~a) Count the number of paid working days that you could do your work (exclude holidays).~~

  ~~b) Count the number of days you could not do your paid work.~~

  ~~c) Count how many of these days you think you could not do your paid work related to the inflammatory bowel disease of your child.~~

- ~~For unpaid work (housekeeping, volunteer work, shopping, gardening, etc.):~~

  ~~d) Count the number of days you could not do your unpaid work.~~

  ~~e) Count how many of these days you think you could not do your unpaid work related to the inflammatory bowel disease of your child.~~

~~If it is a half day, please count 0.5 days. The answers for these five items can be provided at every follow-up visit. Please restart counting from zero after every follow-up visit where you provided this information in the questionnaire.~~

Thank you very much for your help with this research!

FOLLOW-UP VISITS (IDENTIFICATION OF DELETED PARTS)

This sample version is added on the following pages of the pdf-version of this report.

QUESTIONNAIRE:

IMPACT ON CHILDREN's SCHOOL ATTENDANCE ~~AND PARENTS' PRODUCTIVITY~~

(To be filled out at the **follow-up visits**)

Dear parents,

In this study, we would like to try to measure the impact of the health of your child on ~~1)~~ the **school attendance** of your child ~~and 2) the **productivity** of the parents~~.

To do this, we would like to ask you a couple of questions at the start of the research and ask you to provide us with some feedback at every follow-up visit.

Your answers will only be used by the researchers involved in this study and the information collected will then be anonymised.

Your help in this research is really appreciated!

General questions:

Question 1: What is the patient code of the child who is participating in the study?

....

Question 2: On which date are you filling out this questionnaire?

Day		Month		Year			

Here are the questions to be filled out by the parents AT THE FOLLOW-UP VISIT related to SCHOOL ATTENDANCE.

Question 3: Has the school situation of your child changed since the completion of the previous questionnaire? (If yes, go to question 4. If no, go to question 5)

☐ Yes ☐ No

Question 4: Can you give an image of a typical school week? (You can also report half days)

a) How many days does your child go to school in a typical week? (exclude home education)

…… days

b) Does your child follow home schooling (or home education)?

☐ Yes ☐ No

c) If yes, how many hours per week does your child follow home schooling?

On average …… hours per week

d) If yes, is following home schooling a consequence of the inflammatory bowel disease?

☐ Yes ☐ No

Please, continue with the questionnaire on the next page.

Question 5: Can you answer the following questions based on the information collected in your diary?

In the period between the previous time you filled out this questionnaire and this follow-up visit:

a1) How many school days could your child attend during this period?

…… days

b1) How many school days could your child not attend?

…… days

c1) How many of these days (in answer b1) do you think your child could not attend due to the inflammatory bowel disease?

…… days

In case your child is also home schooled:

a2) How many home schooling days could your child attend during this period?

…… days

b2) How many home schooling days could your child not attend?

…… days

c2) How many of these days (in answer b2) do you think your child could not attend due to the inflammatory bowel disease?

…… days

In the next follow-up visit, we will gather the same information based on your diary. You can find a copy of these questions on the last page to take home with you. Please restart counting from zero after you provided this information during this follow-up visit.

~~Please continue on the next page asking questions related to productivity.~~

Here are the questions to be filled out by the parents AT THE FOLLOW-UP VISIT related to parents' work (PRODUCTIVITY).

If there is one parent, please fill out the boxes next to "parent 1". If there are two parents, please fill out both boxes next to "parent 1" and "parent 2".

Question 6: In which month and which year were you born?

Parent 1: Month Year

Parent 2: Month Year

Question 7: What is your gender?

Parent 1: ☐ Male ☐ Female

Parent 2: ☐ Male ☐ Female

Question 8: Are you currently in paid work? (If no, please go to question 11d and 11e where questions related to unpaid work are asked)

Parent 1: ☐ Yes ☐ No

Parent 2: ☐ Yes ☐ No

Question 9: How many hours per week do you usually work? (Only count the hours for which you are paid)

Parent 1: .... hours

Parent 2: .... hours

Question 10: How many days per week do you usually work? (Only count the days for which you are paid)

Parent 1: .... days

Parent 2: .... days

Question 11: Can you answer the following questions based on the information in your diary?

In the period between the previous time you filled out this questionnaire and this follow-up visit:

- For paid work:

  a) How many paid working days could you do your work? (exclusive holidays)

  Parent 1: .... days

  Parent 2: .... days

  b) How many days could you not do your paid work?

  Parent 1: .... days

  Parent 2: .... days

  c) How many of these days (in answer b) do you think you could not do your paid work related to the inflammatory bowel disease of your child?

  Parent 1: .... days

  Parent 2: .... days

- For unpaid work (housekeeping, volunteer work, shopping, gardening, etc.):

  d) How many days could you not do your unpaid work?

  Parent 1: .... days

  Parent 2: .... days

  e) How many of these days (in answer d) do you think you could not do your unpaid work related to the inflammatory bowel disease of your child?

  Parent 1: .... days

  Parent 2: .... days

~~**Question 12a**: Have you asked for help (from family, friends, household help, etc.) due to the inflammatory bowel disease of your child? (in the period after the previous follow-up visit)~~

☐ ~~Yes~~ ☐ ~~No~~

~~**Question 12b**: If yes, on average, how many hours per week do you get help related to the inflammatory bowel disease of your child?~~

~~On average ...... hours per week~~

~~**This is the end of this questionnaire.**~~

~~In the next follow-up visit, we would like you to provide the same information based on your diary. You can find a copy of these questions on the last page to take home with you. Please restart counting from zero after you provided this information during this follow-up visit.~~

~~Thank you very much for your help with this research!~~

INFORMATION FOR PARENTS **TO TAKE HOME**
TO HELP IN COLLECTING INFORMATION
FOR THE NEXT FOLLOW-UP VISIT

Dear parents,

In this questionnaire, we asked you to gather information related to your child's school attendance ~~and your work (productivity)~~. **We would like you to keep a simple diary** (or something similar) to gather this information. **You can take this page ~~and the next one~~ with you to help you to remind you which information can be gathered**.

School attendance:

a1) Count the number of school days your child could attend.

b1) Count the number of school days your child could not attend.

c1) Count how many of these days you think your child could not attend due to the inflammatory bowel disease.

In case your child also follows home schooling (or home education), you can gather the same information:

a2) Count the number of home school days your child could attend.

b2) Count the number of home school days your child could not attend.

c2) Count how many of these days you think your child could not attend due to the inflammatory bowel disease.

If it is a half day, please count 0.5 days. The answers for these three items can be provided at every follow-up visit. Please restart counting from zero after every follow-up visit where you provided this information in the questionnaire.

INFORMATION FOR PARENTS **TO TAKE HOME**
TO HELP IN COLLECTING INFORMATION
FOR THE NEXT FOLLOW-UP VISIT

Productivity: for both parents

- For paid work:

  a) Count the number of paid working days that you could do your work (exclude holidays).

  b) Count the number of days you could not do your paid work.

  c) Count how many of these days you think you could not do your paid work related to the inflammatory bowel disease of your child.

- For unpaid work (housekeeping, volunteer work, shopping, gardening, etc.):

  d) Count the number of days you could not do your unpaid work.

  e) Count how many of these days you think you could not do your unpaid work related to the inflammatory bowel disease of your child.

If it is a half day, please count 0.5 days. The answers for these five items can be provided at every follow-up visit. Please restart counting from zero after every follow-up visit where you provided this information in the questionnaire.

Thank you very much for your help with this research!

APPENDIX 6. WPAI:CD-CG

Work Productivity and Activity Impairment Questionnaire:
Crohn's Disease V2.0 (WPAI:CD) – Caregiver

The following questions ask about the effect of your child's Crohn's Disease on your ability to work and perform normal daily activities. *Please fill in the blanks or circle a number, as indicated.*

1. Are you currently employed (working for pay)? _____ NO _____ YES
   If NO, tick "NO" and skip to question 6.

The next questions refer to the **past seven days**, not including today.

2. During the past seven days, how many hours did you miss from work because of problems associated with your child's Crohn's Disease? *Include hours you missed on sick days, times you went in late, left early, etc., because of your child's Crohn's Disease. Do not include time you missed for your child to participate in this study.*

_____HOURS

3. During the past seven days, how many hours did you miss from work because of any other reason, such as annual leave, holidays, time off for your child to participate in this study?

_____ HOURS

4. During the past seven days, how many hours did you actually work?

_____ HOURS *(If "0", skip to question 6.)*

5. During the past seven days, how much did your child's Crohn's Disease affect your productivity while you were working?

Think about days you were limited in the amount or kind of work you could do, days you accomplished less than you would like, or days you could not do your work as carefully as usual. If your child's Crohn's Disease affected your work only a little, choose a low number. Choose a high number if your child's Crohn's Disease affected your work a great deal.

Consider only how much your child's Crohn's Disease
affected productivity while you were working.

My child's Crohn's Disease had no effect on my work	0	1	2	3	4	5	6	7	8	9	10	My child's Crohn's Disease completely prevented me from working

CIRCLE A NUMBER

6. During the past seven days, how much did your child's Crohn's Disease affect your ability to perform your normal daily activities, excluding your job?

By normal activities, we mean the usual activities you perform, such as working around the house, shopping, childcare, exercising, studying, etc. Think about times you were limited in the amount or kind of activities you could perform and times you accomplished less than you would like. If your child's Crohn's Disease affected your activities only a little, choose a low number. Choose a high number if your child's Crohn's Disease affected your activities a great deal.

Consider only how much your child's Crohn's Disease affected your ability
to perform your normal daily activities, excluding your job.

My child's Crohn's Disease had no effect on my daily activities	0	1	2	3	4	5	6	7	8	9	10	My child's Crohn's Disease completely prevented me from doing my daily activities

CIRCLE A NUMBER
