# Supplementary material for: Identifying Health Economic Considerations to Include in the Research Protocol of a Randomized Controlled Trial (the REDUCE-RISK Trial): Systematic Literature Review and Assessment
Source: JMIR Form Res. 2021 Jan 25;5(1):e13888. doi: 10.2196/13888 (PMC7870354; doi:10.2196/13888)
Supplement: Multimedia Appendix 2 [file formative_v5i1e13888_app2.docx]

Table S1: In- and excluded studies (and reasons for exclusion) based on full text analysis

|  | Reference | Reason for exclusion |
| --- | --- | --- |
| 1 | National Horizon Scanning Centre. Adalimumab (Humira) for moderate to severely active Crohn's disease - horizon scanning review. Report. Birmingham: National Horizon Scanning Centre (NHSC); 2005. | Not available |
| 2 | Dretzke J, Edlin R, Round J, Connock M, Hulme C, Czeczot J, et al. A systematic review and economic evaluation of the use of tumour necrosis factor-alpha (TNF-a) inhibitors, adalimumab and infliximab, for Crohn's disease. HTA Technology Assessment Report. NIHR Health Technology Assessment programme; 2011;15(6). Available from: http://www.hta.ac.uk/1652 | Selected |
| 3 | Assasi N, Blackhouse G, Xie F, Gaebel K, Marshall J, Irvine E, et al. Anti-TNF-a drugs for refractory inflammatory bowel disease: clinical- and cost-effectiveness analyses. Report. Ottawa: Canadian Agency for Drugs and Technologies in Health (CADTH); 2009. Available from: http://www.cadth.ca/media/pdf/H0479_Anti_TNF_a_Drugs_for_Refractory_Inflammatory_Bowel_Disease_tr_e.pdf | Selected |
| 4 | National Institute for Health and Clinical Excellence. Infliximab (review) and adalimumab for the treatment of Crohn’s disease. Report. London: National Institute for Health and Clinical Excellence (NICE); 2010. Available from: http://www.nice.org.uk/nicemedia/live/12985/48552/48552.pdf | Link to other report (NICE TA187) 🡪 see hand searching. |
| 5 | Hayes I. Adalimumab (humira) for treatment of pediatric Crohn's disease. Report. Lansdale, PA: HAYES Inc.; 2015. | To pay for |
| 6 | Hayes I. Humira (Adalimumab; Abbott Laboratories) for treatment of pediatric crohn's disease. Lansdale: HAYES, Inc.. Health Technology Brief Publication. . 2013. | To pay for |
| 7 | Kaplan GG, Hur C, Korzenik J, Sands BE. Infliximab dose escalation vs initiation of adalimumab for loss of response in Crohn's disease: a cost-effectiveness analysis. Alimentary Pharmacology and Therapeutics. 2007;26(11-12):1509-20. | Selected |
| 8 | Bodger K, Kikuchi T, Hughes D. Cost-effectiveness of biological therapy for Crohn's disease: Markov cohort analyses incorporating United Kingdom patient-level cost data. Alimentary Pharmacology and Therapeutics. 2009;30(3):265-74. | Selected |
| 9 | Yu AP, Johnson S, Wang ST, Atanasov P, Tang J, Wu E, et al. Cost utility of adalimumab versus infliximab maintenance therapies in the United States for moderately to severely active Crohn's disease. PharmacoEconomics. 2009;27(7):609-21. | Selected |
| 10 | Loftus EV, Johnson SJ, Yu AP, Wu EQ, Chao J, Mulani PM. Cost-effectiveness of adalimumab for the maintenance of remission in patients with Crohn's disease. European Journal of Gastroenterology and Hepatology. 2009;21(11):1302-9. | Selected |
| 11 | Xie F, Blackhouse G, Assasi N, Gaebel K, Robertson D, Goeree R. Cost-utility analysis of infliximab and adalimumab for refractory ulcerative colitis. Cost Effectiveness and Resource Allocation. 2009;7:20. | Intervention (initial therapy with infliximab and switching to adalimumab in case of no response) |
| 12 | Sandborn WJ, Colombel JF, Schreiber S, Plevy SE, Pollack PF, Robinson AM, et al. Dosage adjustment during long-term adalimumab treatment for Crohn's disease: clinical efficacy and pharmacoeconomics. Inflammatory Bowel Diseases. 2011;17(1):141-51. | Study design (cost analysis) |
| 13 | Blackhouse G, Assasi N, Xie F, Marshall J, Irvine EJ, Gaebel K, et al. Canadian cost-utility analysis of initiation and maintenance treatment with anti-TNF-alpha drugs for refractory Crohn's disease. Journal of Crohn's and Colitis. 2012;6(1):77-85. | Selected |
| 14 | Liu Y, Wu EQ, Bensimon AG, Fan CP, Bao Y, Ganguli A, et al. Cost per responder associated with biologic therapies for crohn's disease, psoriasis, and rheumatoid arthritis. Advances in Therapy. 2012;29(7):620-34. | Outcome (cost per responder) |
| 15 | Lofland JH, Mallow P, Rizzo J. Cost-per-remission analysis of infliximab compared to adalimumab among adults with moderate-to-severe ulcerative colitis. Journal of Medical Economics. 2013;16(4):461-7. | Outcome (cost per remission) |
| 16 | van der Valk ME, Mangen MJ, Severs M, van der Have M, Dijkstra G, van Bodegraven AA, et al. Comparison of Costs and Quality of Life in Ulcerative Colitis Patients with an Ileal Pouch-Anal Anastomosis, Ileostomy and Anti-TNFalpha Therapy. Journal of Crohn's & colitis. 2015;9(11):1016-23. | Study design (cost analysis) |
| 17 | Black CM, Yu E, McCann E, Kachroo S. Dose Escalation and Healthcare Resource Use among Ulcerative Colitis Patients Treated with Adalimumab in English Hospitals: An Analysis of Real-World Data. PLoS ONE [Electronic Resource]. 2016;11(2):e0149692. | Study design (cost analysis) |
| 18 | Park T, Griggs SK, Suh DC. Cost Effectiveness of Monoclonal Antibody Therapy for Rare Diseases: A Systematic Review. Biodrugs. 2015;29(4):259-74. | Study design (review – references in study are already identified) |
| 19 | Toor K, Druyts E, Jansen JP, Thorlund K. Cost per remission and cost per response with infliximab, adalimumab, and golimumab for the treatment of moderately-to-severely active ulcerative colitis. Journal of Medical Economics. 2015;18(6):437-46. | Outcome (cost per remission) |
| 20 | Wu N, Lee YC, Shah N, Harrison DJ. Cost of biologics per treated patient across immune-mediated inflammatory disease indications in a pharmacy benefit management setting: a retrospective cohort study. Clinical Therapeutics. 2014;36(8):1231-41, 41.e1-3. | Study design (cost analysis) |
| 21 | Saro C, Ceballos D, Munoz F, De la Coba C, Aguilar MD, Lazaro P, et al. Resources Utilization and Costs the Year Before and After Starting Treatment with Adalimumab in Crohn's Disease Patients. Inflammatory Bowel Diseases. 2015;21(7):1631-40. | Comparator (Before-after analyses) |
| 22 | Xie F. The economics of adalimumab for ulcerative colitis. Expert Review of Pharmacoeconomics & Outcomes Research. 2015;15(3):373-7. | Study design (review – references in study are already identified) |
| 23 | Mandel MD, Balint A, Golovics PA, Vegh Z, Mohas A, Szilagyi B, et al. Decreasing trends in hospitalizations during anti-TNF therapy are associated with time to anti-TNF therapy: Results from two referral centres. Digestive & Liver Disease. 2014;46(11):985-90. | Study design |
| 24 | Brekke KR, Dalen DM, Holmas TH. Diffusion of pharmaceuticals: cross-country evidence of anti-TNF drugs. European Journal of Health Economics. 2014;15(9):937-51. | Study design |
| 25 | Pariente B, Laharie D. Review article: why, when and how to de-escalate therapy in inflammatory bowel diseases. Alimentary Pharmacology & Therapeutics. 2014;40(4):338-53. | Study design (review – references in study are already identified) |
| 26 | Gulacsi L, Rencz F, Pentek M, Brodszky V, Lopert R, Hever NV, et al. Transferability of results of cost utility analyses for biologicals in inflammatory conditions for Central and Eastern European countries. European Journal of Health Economics. 2014;15 Suppl 1:S27-34. | Study design (no primary economic evaluation – references in study are already identified) |
| 27 | Lapadula G, Marchesoni A, Armuzzi A, Blandizzi C, Caporali R, Chimenti S, et al. Adalimumab in the treatment of immune-mediated diseases. International Journal of Immunopathology & Pharmacology. 2014;27(1 Suppl):33-48. | Study design (review – references in study are already identified) |
| 28 | Choi GK, Collins SD, Greer DP, Warren L, Dowson G, Clark T, et al. Costs of adalimumab versus infliximab as first-line biological therapy for luminal Crohn's disease. Journal of Crohn's & colitis. 2014;8(5):375-83. | Study design (cost analysis) |
| 29 | van der Valk ME, Mangen MJ, Leenders M, Dijkstra G, van Bodegraven AA, Fidder HH, et al. Healthcare costs of inflammatory bowel disease have shifted from hospitalisation and surgery towards anti-TNFalpha therapy: results from the COIN study. Gut. 2014;63(1):72-9. | Study design (cost analysis) |
| 30 | Tang DH, Harrington AR, Lee JK, Lin M, Armstrong EP. A systematic review of economic studies on biological agents used to treat Crohn's disease. Inflammatory Bowel Diseases. 2013;19(12):2673-94. | Study design (review – references in study are already identified) |
| 31 | Laki J, Monok G, Palosi M, Gajdacsi JZ. Economical aspect of biological therapy in inflammatory conditions in Hungary. Expert Opinion on Biological Therapy. 2013;13(3):327-37. | Study design |
| 32 | Sussman DA, Kubiliun N, Mulani PM, Chao J, Gillis CA, Yang M, et al. Comparison of medical costs among patients using adalimumab and infliximab: a retrospective study (COMPAIRS). Inflammatory Bowel Diseases. 2012;18(11):2043-55. | Study design (cost analysis) |
| 33 | Thomson AB, Gupta M, Freeman HJ. Use of the tumor necrosis factor-blockers for Crohn's disease. World Journal of Gastroenterology. 2012;18(35):4823-54. | Study design (review – references in study are already identified) |
| 34 | Tang DH, Armstrong EP, Lee JK. Cost-utility analysis of biologic treatments for moderate-to-severe Crohn's disease. Pharmacotherapy:The Journal of Human Pharmacology & Drug Therapy. 2012;32(6):515-26. | Selected |
| 35 | Di Sabatino A, Liberato L, Marchetti M, Biancheri P, Corazza GR. Optimal use and cost-effectiveness of biologic therapies in inflammatory bowel disease. Internal & Emergency Medicine. 2011;6 Suppl 1:17-27. | Study design (review – references in study are already identified) |
| 36 | Binion DG, Louis E, Oldenburg B, Mulani P, Bensimon AG, Yang M, et al. Effect of adalimumab on work productivity and indirect costs in moderate to severe Crohn's disease: a meta-analysis. Canadian Journal of Gastroenterology. 2011;25(9):492-6. | Study design |
| 37 | Symmons DP. Anti-tumour necrosis factor alpha therapy: can we afford it? Annals of the Rheumatic Diseases. 2005;64(7):969-70. | Study design |
| 38 | Wong JB. Cost-effectiveness of anti-tumor necrosis factor agents. Clinical & Experimental Rheumatology. 2004;22(5 Suppl 35):S65-70. | Study design (review – references in study are already identified) |
| 39 | Tappenden P, Ren S, Archer R, Harvey R, James MM, Basarir H, et al. A Model-Based Economic Evaluation of Biologic and Non-Biologic Options for the Treatment of Adults with Moderately-to-Severely Active Ulcerative Colitis after the Failure of Conventional Therapy. Pharmacoeconomics. 2016;34(10):1023-38. | Selected |
| 40 | Archer R, Tappenden P, Ren S, Martyn-St James M, Harvey R, Basarir H, et al. Infliximab, adalimumab and golimumab for treating moderately to severely active ulcerative colitis after the failure of conventional therapy (including a review of TA140 and TA262): clinical effectiveness systematic review and economic model. Health Technology Assessment (Winchester, England). 2016;20(39):1-326. | Selected |
| 41 | Yokomizo L, Limketkai B, Park KT. Cost-effectiveness of adalimumab, infliximab or vedolizumab as first-line biological therapy in moderate-to-severe ulcerative colitis. BMJ Open Gastroenterology. 2016;3(1):e000093. | Outcome (cost per mucosal healing) |
| 42 | Roblin X, Attar A, Lamure M, Savarieau B, Brunel P, Duru G, et al. Cost savings of anti-TNF therapy using a test-based strategy versus an empirical dose escalation in Crohn's disease patients who lose response to infliximab. Journal of Market Access & Health Policy. 2015;3. | Intervention |
| 43 | Degli Esposti L, Sangiorgi D, Perrone V, Radice S, Clementi E, Perone F, et al. Adherence and resource use among patients treated with biologic drugs: findings from BEETLE study. Clinicoeconomics & Outcomes Research. 2014;6:401-7. | Study design (cost analysis) |
| 44 | Gladman DD, Brown RE. Pharmacoeconomics of adalimumab for rheumatoid arthritis, psoriatic arthritis, ankylosing spondylitis and Crohn's disease. Expert Review of Pharmacoeconomics & Outcomes Research. 2008;8(2):111-25. | Study design (review – references in study are already identified) |
| 45 | Sartini A, Di Girolamo M, Bertani A, Villa E. "Blindly" ADA Dose Escalation to 80 mg Weekly in Crohn's Disease Patients with LOR: Is It Cost Effective or Not? Inflammatory Bowel Diseases. 2015;21(11):E27-8. | Study design |
| 46 | Eshuis EJ, Stokkers PC, Bemelman WA. Rectification of miscalculation of adalimumab costs. Expert review of gastroenterology & hepatology. 2011;5(4):437-8. | Study design |
| 47 | Stawowczyk E, Kawalec P, Pilc A. Cost-utility analysis of 1-year treatment with adalimumab/standard care and standard care alone for ulcerative colitis in Poland. European Journal of Clinical Pharmacology. 2016((Stawowczyk E.) StatSoft Polska Sp. z o.o., Krakow, Poland):1-7. | Selected |
| 48 | Suzzoni S, Aubourg A, Pourrat X. Management of Crohn's disease by biologics in France: A retrospective pairwise economic study comparing cost per remitter associated with adalimumab and infliximab treatment. International Journal of Clinical Pharmacy. 2016;38(6):568-9. | Not available |
| 49 | Beilman C, Nguyen T, Ung V, Ma C, Wong K, Kroeker K, et al. Cost-utility analysis shows adalimumab is cost-effective for the management of ulcerative colitis. Canadian Journal of Gastroenterology and Hepatology. 2016. | Abstract |
| 50 | Van Der Valk ME, Mangen MJJ, Severs M, Van Der Have M, Dijkstra G, Van Bodegraven AA, et al. Evolution of costs of inflammatory bowel disease over two years of follow-up. PLoS ONE. 2016;11(4). | Study design (cost analysis) |
| 51 | Beilman CL, Nguyen T, Ung V, Ma C, Wong K, Kroeker K, et al. Cost-utility analysis shows adalimumab is cost-effective for the management of ulcerative colitis. Gastroenterology. 2016;150(4):S631-S2. | Abstract |
| 52 | Travis S, Feagan BG, Peyrin-Biroulet L, Panaccione R, Danese S, Lazar A, et al. The costs of care for patients with ulcerative colitis: Effect of adalimumab on health care resources utilisation in clinical practice from INSPIRADA. Gastroenterology. 2016;150(4):S631. | Study design (cost analysis) |
| 53 | Essat M, Tappenden P, Ren S, Bessey A, Archer R, Wong R, et al. Vedolizumab for the Treatment of Adults with Moderate-to-Severe Active Ulcerative Colitis: An Evidence Review Group Perspective of a NICE Single Technology Appraisal. PharmacoEconomics. 2016;34(3):245-57. | Selected |
| 54 | Huoponen S, Blom M. A systematic review of the cost-effectiveness of biologics for the treatment of inflammatory bowel diseases. PLoS ONE. 2015;10(12). | Study design (review – references in study are already identified) |
| 55 | Sin AT, Damman JL, Ziring DA, Gleghorn EE, Garcia-Careaga MG, Gugig RR, et al. Out-of-pocket cost burden in pediatric inflammatory bowel disease: A cross-sectional cohort analysis. Inflammatory Bowel Diseases. 2015;21(6):1368-77. | Study design (cost analysis) |
| 56 | Burisch J, Vardi H, Pedersen N, Brinar M, Cukovic-Cavka S, Kaimakliotis I, et al. Costs and resource utilization for diagnosis and treatment during the initial year in a european inflammatory bowel disease inception cohort: An ECCO-EpiCom study. Inflammatory Bowel Diseases. 2015;21(1):121-31. | Study design (cost analysis) |
| 57 | Augustine JM, Lee JK, Armstrong EP. Health outcomes and cost-effectiveness of certolizumab pegol in the treatment of Crohn's disease. Expert Review of Pharmacoeconomics and Outcomes Research. 2014;14(5):599-609. | Study design (no primary economic evaluation – references in study are already identified) |
| 58 | Marchetti M, Liberato NL. Biological therapies in Crohn's disease: Are they cost-effective? A critical appraisal of model-based analyses. Expert Review of Pharmacoeconomics and Outcomes Research. 2014;14(6):815-24. | Study design (review – references in study are already identified) |
| 59 | Lee JK, Tang DH, Mollon L, Armstrong EP. Cost-effectiveness of biological agents used in ulcerative colitis. Best Practice and Research: Clinical Gastroenterology. 2013;27(6):949-60. | Study design (review – references in study are already identified) |
| 60 | Rocchi A, Benchimol E, Bernstein CN, Bitton A, Feagan B, Panaccione R, et al. Inflammatory bowel disease: A Canadian burden of illness review. Canadian Journal of Gastroenterology. 2012;26(11):811-7. | Study design |
| 61 | Mobinizadeh M, Oliyaeemanesh A, Doaee S, Nejati M, Aboee P, Azadbakht M, et al. Health technology assessment of infliximab: A rapid review of type 2 studies. Journal of Isfahan Medical School. 2012;30(178). | Language |
| 62 | Buchanan J, Wordsworth S, Ahmad T, Perrin A, Vermeire S, Sans M, et al. Managing the long term care of inflammatory bowel disease patients: The cost to European health care providers. Journal of Crohn's and Colitis. 2011;5(4):301-16. | Study design (cost analysis) |
| 63 | Ghosh S. Estimating benefits of therapy in Crohn's disease in terms of indirect costs. Canadian Journal of Gastroenterology. 2011;25(8):412. | Study design (cost analysis) |
| 64 | Park KT, Bass D. Inflammatory bowel disease-attributable costs and cost-effective strategies in the United States: A review. Inflammatory Bowel Diseases. 2011;17(7):1603-9. | Study design (review – references in study are already identified) |
| 65 | Bodger K. Cost effectiveness of treatments for inflammatory bowel disease. PharmacoEconomics. 2011;29(5):387-401. | Study design (review – references in study are already identified) |
| 66 | Mesterton J, Jönsson L, Almer SHC, Befrits R, Friis-Liby I, Lindgren S. Resource use and societal costs for Crohn's disease in Sweden. Inflammatory Bowel Diseases. 2009;15(12):1882-90. | Study design (cost analysis) |
| 67 | Zisman TL, Cohen RD. Pharmacoeconomics and quality of life of current and emerging biologic therapies for inflammatory bowel disease. Current Treatment Options in Gastroenterology. 2007;10(3):185-94. | Study design (review) |
| Extra references identified through hand searching | | |
| 1 | Infliximab and adalimumab for the treatment of Crohn’s disease. Technology appraisal guidance. Published: 19 May 2010 (<https://www.nice.org.uk/guidance/ta187>) | Selected |
| 2 | CADTH, 2014 (SR0341_Simponi_PE_Report_e)  Canadian Agency for Drugs and Technologies in Health (CADTH). Common Drug Review Pharmacoeconomic Review Report for Simponi. November 2014. | Selected |
| 3 | Essat M, Tappenden P, Ren S, Bessey A, Archer R, Wong R, Hoque S, Lobo A. Vedolizumab for the treatment of adults with moderately to severely active ulcerative colitis: A Single Technology Appraisal. School of Health and Related Research (ScHARR), 2014. | Selected |
| 4 | Rafia R, Scope A, Harnan S, Stevens JW, Stevenson M, Sutton A, Dickinson K, Parkes M, Mayberry J, Lobo A. Vedolizumab for the treatment of adults with moderately to severely active Crohn’s disease: A Single Technology Appraisal. School of Health and Related Research (ScHARR), 2014. | Selected |

Table S2: List of selected economic evaluations

| HTA reports |
| --- |
| 1. Archer R, Tappenden P, Ren S, Martyn-St James M, Harvey R, Basarir H, et al. Infliximab, adalimumab and golimumab for treating moderately to severely active ulcerative colitis after the failure of conventional therapy (including a review of TA140 and TA262): clinical effectiveness systematic review and economic model. Health Technol Assess 2016;20(39). (study 1/16*)  - AbbVie. Adalimumab, golimumab and infliximab, for the treatment of ulcerative colitis (subacute). Submission to NICE; 2014. 🡪 reported on page 149-163 in the report of Archer et al. - MSD. Manufacturer submission of evidence: infliximab (Remicade). Submission to NICE; 2014 and MSD. Manufacturer submission of evidence: golimumab (Simponi). Submission to NICE; 2014. 🡪 reported on page 130-149 in the report of Archer et al. - Tappenden P, Ren S, Archer R, Harvey R, James MM, Basarir H, et al. A model-based economic evaluation of biologic and non-Biologic options for the treatment of adults with moderately-to-severely active ulcerative colitis after the failure of conventional therapy. Pharmacoeconomics. 2016 Oct;34(10):1023-38. (study 2/16)   *Remark: this journal article is based on the full HTA report of Archer et al. and is therefore not included separately in our overview.* |
| 1. Assasi N, Blackhouse G, Xie F, Gaebel K, Marshall J, Irvine EJ, Giacomini M, Robertson D, Campbell K, Hopkins R, Goeree R. Anti-TNF-α drugs for refractory inflammatory bowel disease: Clinical- and cost-effectiveness analyses [Technology report number 120]. Ottawa: Canadian Agency for Drugs and Technologies in Health; 2009. (study 3/16)  - Blackhouse G, Assasi N, Xie F, Marshall J, Irvine EJ, Gaebel K, et al. Canadian cost-utility analysis of initiation and maintenance treatment with anti-TNF-alpha drugs for refractory Crohn's disease. Journal of Crohn's and Colitis. 2012 21 Jul 2012;6(1):77-85. (study 4/16)   *Remark: this journal article is based on the full HTA report of Assasi et al. and is therefore not included separately in our overview.* |
| 1. Canadian Agency for Drugs and Technologies in Health (CADTH). Common drug review pharmacoeconomic review report for Simponi. November 2014. (study 5/16) |
| 1. Dretzke J, Edlin R, Round J, Connock M, Hulme C, Czeczot J, et al. A systematic review and economic evaluation of the use of tumour necrosis factor-alpha (TNF-α) inhibitors, adalimumab and infliximab, for Crohn’s disease. Health Technol Assess 2011;15(6). (study 6/16)  - Critique of the submission on adalimumab by Abbott. 🡪 reported on page 109-120 in the report of Dretzke et al. - National Institute for Health and Care Excellence (NICE). Infliximab and adalimumab for the treatment of Crohn's disease's (TA187); May 2010. ([www.nice.org.uk/guidance/ta187/resources/infliximab-review-and-adalimumab-for-the-treatment-of-crohns-disease-82598501180869](http://www.nice.org.uk/guidance/ta187/resources/infliximab-review-and-adalimumab-for-the-treatment-of-crohns-disease-82598501180869)) (study 7/16)   *Remark: the results mentioned in the economic part of this report are based on the full HTA report of Dretzke et al. and is therefore not included separately in our overview.* |
| 1. Essat M, Tappenden P, Ren S, Bessey A, Archer R, Wong R, Hoque S, Lobo A. Vedolizumab for the treatment of adults with moderately to severely active ulcerative colitis: A Single Technology Appraisal. School of Health and Related Research (ScHARR), 2014. (study 8/16)  - Essat M, Tappenden P, Ren S, Bessey A, Archer R, Wong R, et al. Vedolizumab for the treatment of adults with moderate-to-severe active ulcerative colitis: an evidence review group perspective of a NICE single technology appraisal. PharmacoEconomics. 2016;34(3):245-57. (study 9/16)   *Remark: this journal article is based on the full HTA report of Essat et al. and is therefore not included separately in our overview.* |
| 1. Rafia R, Scope A, Harnan S, Stevens JW, Stevenson M, Sutton A, Dickinson K, Parkes M, Mayberry J, Lobo A. Vedolizumab for the treatment of adults with moderately to severely active Crohn’s disease: A Single Technology Appraisal. School of Health and Related Research (ScHARR), 2014. (study 10/16) |
| Journal articles |
| 1. Bodger K, Kikuchi T, Hughes D. Cost-effectiveness of biological therapy for Crohn's disease: Markov cohort analyses incorporating United Kingdom patient-level cost data. Alimentary Pharmacology and Therapeutics. 2009;30(3):265-74. (study 11/16) |
| 1. Kaplan GG, Hur C, Korzenik J, Sands BE. Infliximab dose escalation vs initiation of adalimumab for loss of response in Crohn's disease: a cost-effectiveness analysis. Alimentary Pharmacology and Therapeutics. 2007;26(11-12):1509-20. (study 12/16) |
| 1. Loftus EV, Johnson SJ, Yu AP, Wu EQ, Chao J, Mulani PM. Cost-effectiveness of adalimumab for the maintenance of remission in patients with Crohn's disease. European Journal of Gastroenterology and Hepatology. 2009;21(11):1302-9. (study 13/16)  - Remark: Dretzke et al. refer to this study that was funded and supported by the Abbott Laboratories. However, since this article reports somewhat different results, we also include it in our overview. |
| 1. Stawowczyk E, Kawalec P, Pilc A. Cost-utility analysis of 1-year treatment with adalimumab/standard care and standard care alone for ulcerative colitis in Poland. European Journal of Clinical Pharmacology. 2016. StatSoft Polska Sp. z o.o., Krakow, Poland):1-7. (study 14/16) |
| 1. Tang DH, Armstrong EP, Lee JK. Cost-utility analysis of biologic treatments for moderate-to-severe Crohn's disease. Pharmacotherapy:The Journal of Human Pharmacology & Drug Therapy. 2012;32(6):515-26. (study 15/16) |
| 1. Yu AP, Johnson S, Wang ST, Atanasov P, Tang J, Wu E, et al. Cost utility of adalimumab versus infliximab maintenance therapies in the United States for moderately to severely active Crohn's disease. PharmacoEconomics. 2009;27(7):609-21. (study 16/16) |

** The 16 studies identified in our search strategy are numbered in this table (study x/16). Finally, 12 original economic evaluations are included (due to an overlap between full HTA reports and journal articles).*
