# Supplementary material for: Identifying Health Economic Considerations to Include in the Research Protocol of a Randomized Controlled Trial (the REDUCE-RISK Trial): Systematic Literature Review and Assessment
Source: JMIR Form Res. 2021 Jan 25;5(1):e13888. doi: 10.2196/13888 (PMC7870354; doi:10.2196/13888)
Supplement: Multimedia Appendix 3 [file formative_v5i1e13888_app3.doc]

| **Section/topic** | **#** | **Checklist item** | **Reported on page #** |
| --- | --- | --- | --- |
| **TITLE** | | |  |
| Title | 1 | Identify the report as a systematic review, meta-analysis, or both. | Page 1 |
|  |  | “*The REDUCE-RISK trial: a systematic review of the economic literature to provide input to the research protocol from a health economic point of view*” |  |
| **ABSTRACT** | | |  |
| Structured summary | 2 | Provide a structured summary including, as applicable: background; objectives; data sources; study eligibility criteria, participants, and interventions; study appraisal and synthesis methods; results; limitations; conclusions and implications of key findings; systematic review registration number. | Page 3 |
|  |  | “*Background: The REDUCE-RISK trial is set up to compare the effectiveness of weekly subcutaneously administered methotrexate with daily oral azathioprine/6-mercaptopurine in low risk Crohn’s disease (CD) or subcutaneously administered adalimumab (ADA) in high risk CD in a paediatric population (age 6-17).*  *Objective: A systematic economic literature review was performed to provide input to the research protocol in order to gather the necessary information to allow the performance of an evidence-based economic evaluation when the trial is finished.*  *Methods: A) Data sources: The Centre for Reviews and Dissemination (CRD) Health Technology Assessment (HTA) database, websites of HTA institutes, CRD’s National Health Service Economic Evaluation Database (NHS EED), Medline (OVID), and EMBASE databases were consulted to retrieve (reviews of) relevant economic evaluations. B) Eligibility criteria: Studies were eligible if they included a paediatric or adult population with inflammatory bowel diseases (CD and ulcerative colitis (UC)) treated with ADA (Humira®). There were no restrictions for the comparator. Only economic evaluations expressing outcomes in life-years gained or quality-adjusted life years gained were selected.*  *Results: Twelve primary studies were identified. None of these studies included a paediatric population due to a lack of supporting trials. The economic evaluations identified in our systematic review indicate ADA is an appropriate intervention for inclusion in such a trial. From a health economic point of view, it is important to make an incremental analysis comparing such an intervention with standard care, and not immediately versus another (expensive) biological treatment. Information on the impact of children’s school attendance and parents’ productivity is currently lacking in the economic evaluations and none of the underlying trials measured QoL with a generic utility instrument.*  *Conclusions: The review of the economic literature of ADA for the treatment of patients with CD supports the performance of a trial with biologicals in paediatric patients, including making a distinction according to severity of disease. Carrying out an economic literature review has allowed us to decide which variables should be added to the research protocol from an economic point of view. Measurements for children’s and parents’ quality of life (EQ-5D questionnaires), children’s school attendance and parents’ productivity (WPAI-CD-CG questionnaire) were added to the research protocol. This will provide support for the calculation of the cost-effectiveness of the interventions evaluated in the REDUCE-RISK trial.*  *Registration REDUCE-RISK trial: ClinicalTrials.gov NCT02852694, https://clinicaltrials.gov/ct2/show/NCT02852694*” |  |
| **INTRODUCTION** | | |  |
| Rationale | 3 | Describe the rationale for the review in the context of what is already known. | Page 5 |
|  |  | “*A review of the literature to provide input to the research protocol*  *In preparation of a future economic evaluation, we determine which are the most important incremental elements. The ISPOR (International Society for Pharmacoeconomics and Outcomes Research) guidelines state that “assessing relative value is rarely the primary purpose of an experimental study. Nevertheless, when the decision is made to conduct an economic evaluation alongside a clinical trial, it is important that the economic investigator contributes to the design of the study to ensure that the trial will provide the data necessary for a high-quality economic evaluation”[4]. Our research question is “which additional elements should we include in the research protocol of the REDUCE-RISK trial to provide support to a high-quality economic evaluation?”. Therefore, a systematic search for economic literature about the cost-effectiveness of ADA (Humira®) for the treatment of inflammatory bowel disease (IBD) was performed.*  *The purpose of this systematic review is to get more useful insights and knowledge from previous economic studies [5]. These previous economic evaluations guide us in finding the key variables which enables us to provide well-directed input for the research protocol. In this paper the review of the economic literature is transparently presented. No official review protocol was set up for the systematic review. The findings are used to provide input for the research protocol from a health economic point of view (e.g. to decide which questionnaires should be added to the research protocol). On the other hand, we also want to avoid overloading the research protocol and only focus on the incremental elements which influence an intervention’s cost-effectiveness. The results of this systematic review help us to focus on gathering the right information in the REDUCE-RISK trial, which will support researchers at the end of the trial to make a high-quality economic evaluation.*” |  |
| Objectives | 4 | Provide an explicit statement of questions being addressed with reference to participants, interventions, comparisons, outcomes, and study design (PICOS). | Page 5 |
|  |  | “*Our research question is “which additional elements should we include in the research protocol of the REDUCE-RISK trial to provide support to a high-quality economic evaluation?”. Therefore, a systematic search for economic literature about the cost-effectiveness of ADA (Humira®) for the treatment of inflammatory bowel disease (IBD) was performed.*” |  |
| **METHODS** | | |  |
| Protocol and registration | 5 | Indicate if a review protocol exists, if and where it can be accessed (e.g., Web address), and, if available, provide registration information including registration number. | Page 5 |
|  |  | “*No official review protocol was set up for the systematic review.*” |  |
| Eligibility criteria | 6 | Specify study characteristics (e.g., PICOS, length of follow-up) and report characteristics (e.g., years considered, language, publication status) used as criteria for eligibility, giving rationale. | Page 6 |
|  |  | **“***Methods*  *A systematic review of the literature was conducted using pre-defined selection criteria that included considerations of population, intervention, comparator, and design. Since the goal of this literature review was to provide input for the research protocol, applied selection criteria were not too restrictive. Studies were included if 1) the population included children or adults with IBD (Crohn’s disease (CD) and ulcerative colitis (UC)); 2) ADA was one of the included interventions; and 3) the design reflected a full economic evaluation, i.e. studies comparing at least two alternative treatments in terms of costs and outcomes, expressing outcomes in life-years gained (LYG) or quality-adjusted life years (QALYs) gained. No restrictions were applied for the comparator. Studies were excluded if they only considered other treatments then ADA at the moment of randomization. Studies that only included switching to ADA in case of no response to the interventions under consideration (i.e. not including ADA at the moment of randomization) are not selected. Cost analysis or cost-of-illness studies do not fulfil the above definition of an economic evaluation and were excluded. As summarized in a EUnetHTA guideline providing an overview of national guidelines for 25 countries [3], “all countries except four specify that the preferred outcome measure is QALYs or both QALYs and life years. Of the four countries with guidelines that do not announce QALYs as a preferred method, at least three accept QALYs in special circumstances or in complementary analyses.” Also in this overview we focus on these preferred outcomes. Studies expressing results in disease-specific outcomes (e.g. cost per remission [6, 7], cost per responder [8], or cost per mucosal healing [9]) are thus excluded. ‘Before-after’ analyses [10] comparing the costs before and after the start of treatment with ADA were also excluded since they also do not fulfil the definition of an economic evaluation (i.e. lack of a comparative intervention). Abstracts were excluded due to a lack of sufficient details to allow a proper evaluation. No time restriction was imposed. English, French, German and Dutch articles were eligible.*” |  |
| Information sources | 7 | Describe all information sources (e.g., databases with dates of coverage, contact with study authors to identify additional studies) in the search and date last searched. | Page 6 |
|  |  | “*Various databases were consulted. In first instance (February 2016), before the final protocol was set up, reviews on this topic were searched by consulting the Centre for Reviews and Dissemination (CRD) Health Technology Assessment (HTA) database and websites of HTA institutes listed on the International Network of Agencies for Health Technology Assessment (INAHTA) website. Websites of ex- or non-member HTA institutes such as the National Institute for Health and Care Excellence (NICE) were also consulted. In September 2016, CRD’s National Health Service Economic Evaluation Database (NHS EED), Medline (OVID), and EMBASE databases were searched to retrieve both full economic evaluations and reviews of full economic evaluations of ADA for IBD treatment. To assure reproducibility, further details of the search strategy are provided in appendix 1 of the supplementary report.*” |  |
| Search | 8 | Present full electronic search strategy for at least one database, including any limits used, such that it could be repeated. | Page 6 |
|  |  | To assure reproducibility, these details are provided for all databases in appendix 1 of the supplementary report. Appendix 1 ‘search strategy economic evaluations’ (p93-99) provides a transparent overview of the search strategy including an overview of the INAHTA websites and databases searched, the date of the search strategy, the search terms and number of hits per search term, number of identified references per database, and the total number of identified references excluding duplicates.  The following in mentioned in the text: “*To assure reproducibility, further details of the search strategy are provided in appendix 1 of the supplementary report.*” |  |
| Study selection | 9 | State the process for selecting studies (i.e., screening, eligibility, included in systematic review, and, if applicable, included in the meta-analysis). | Page 6 |
|  |  | “*The selection of relevant articles was performed in a two-step procedure: initial assessment of the title, abstract, and keywords, followed by a full-text assessment of the selected references. When no abstract was available and the citation was unclear or ambiguous, consideration of the citation was directly made on the basis of a full-text assessment. Reference lists of the selected studies were checked for additional relevant citations. The procedure was performed by a health economist (MN) and in case of doubt for medical reasons, a medical specialist (GV) provided support.*” |  |
| Data collection process | 10 | Describe method of data extraction from reports (e.g., piloted forms, independently, in duplicate) and any processes for obtaining and confirming data from investigators. | Page 6 |
|  |  | “*The primary full economic evaluations were summarized in an in-house data extraction sheet listing all variables (e.g. population, intervention, comparator(s), quality of life (QoL) input, etc.) and summary measures (e.g. ICERs and results of sensitivity analyses) for which data were sought (see appendix 2 of the supplementary report). The gathered information in these sheets reflects the elements that are usually reported in an economic evaluation (e.g. according to the CHEERS (Consolidated Health Economic Evaluation Reporting Standards) guidelines [11, 12]). This information is used to set up the summary tables which forms the basis for a further critical assessment. Based on the results of this assessment, we judge whether from an HTA and economic perspective, elements in the research protocol of the REDUCE-RISK trial could be added.*” |  |
| Data items | 11 | List and define all variables for which data were sought (e.g., PICOS, funding sources) and any assumptions and simplifications made. | Page 6 |
|  |  | The information that was extracted from the identified economic evaluations is presented in the supplementary report: appendix 2 ‘data extraction sheet’. This table includes the 19 elements that were extracted from the original economic evaluation. We refer to this appendix in the text (see above). |  |
| Risk of bias in individual studies | 12 | Describe methods used for assessing risk of bias of individual studies (including specification of whether this was done at the study or outcome level), and how this information is to be used in any data synthesis. | NA |
|  |  | This item is not relevant for this systematic review of economic evaluations. |  |
| Summary measures | 13 | State the principal summary measures (e.g., risk ratio, difference in means). | Page 6 |
|  |  | “*As summarized in a EUnetHTA guideline providing an overview of national guidelines for 25 countries [3], “all countries except four specify that the preferred outcome measure is QALYs or both QALYs and life years. Of the four countries with guidelines that do not announce QALYs as a preferred method, at least three accept QALYs in special circumstances or in complementary analyses.” Also in this overview we focus on these preferred outcomes. Studies expressing results in disease-specific outcomes (e.g. cost per remission [6, 7], cost per responder [8], or cost per mucosal healing [9]) are thus excluded. ‘Before-after’ analyses [10] comparing the costs before and after the start of treatment with ADA were also excluded since they also do not fulfil the definition of an economic evaluation (i.e. lack of a comparative intervention).*” |  |
| Synthesis of results | 14 | Describe the methods of handling data and combining results of studies, if done, including measures of consistency (e.g., I^2^) for each meta-analysis. | NA |
|  |  | This item is not relevant for this systematic review of economic evaluations. At the end of the methods section we mention the following: “*Based on the results of this assessment, we judge whether from an HTA and economic perspective, elements in the research protocol of the REDUCE-RISK trial could be added.*” |  |

Page 1 of 2

| **Section/topic** | **#** | **Checklist item** | **Reported on page #** |
| --- | --- | --- | --- |
| Risk of bias across studies | 15 | Specify any assessment of risk of bias that may affect the cumulative evidence (e.g., publication bias, selective reporting within studies). | NA |
|  |  | This item is not relevant for this systematic review of economic evaluations. |  |
| Additional analyses | 16 | Describe methods of additional analyses (e.g., sensitivity or subgroup analyses, meta-regression), if done, indicating which were pre-specified. | NA |
|  |  | This item is not relevant for this systematic review of economic evaluations. |  |
| **RESULTS** | | |  |
| Study selection | 17 | Give numbers of studies screened, assessed for eligibility, and included in the review, with reasons for exclusions at each stage, ideally with a flow diagram. | Page 7 |
|  |  | “*Figure 1 presents the flow chart of the selection process. Twelve articles were identified in electronic databases. Four additional references were identified through searching websites of HTA institutes. Information from three journal articles [13-15] and one report [16] were already published in an HTA report. To avoid overlap, only the 12 primary studies [17-28] will be further discussed. The list of the 16 identified economic evaluations and information on duplicates is provided in Table 2 of the supplementary report.*  *Figure 1: Selection of relevant articles*    ** Databases searched: Centre for Reviews and Dissemination (CRD) databases (NHS Economic Evaluation Database (NHS EED) and Health Technology Assessments (HTA)), Medline (OVID), and Embase.*”  In the supplementary files for review, we also included an overview of the reasons for exclusion for the 67 ‘full-text articles assessed for eligibility’. This file is available upon request. |  |
| Study characteristics | 18 | For each study, present characteristics for which data were extracted (e.g., study size, PICOS, follow-up period) and provide the citations. | Page 8-10 |
|  |  | In the text, we provide a summary of ‘general information’ and ‘population and compared interventions’. It is not possible to include all these overview tables in this article. For further details, therefore, we prefer to refer to these overview tables in the supplementary report.  “***General information***  *Half of the studies were performed for the UK (n=6) (Table 1). Two studies made an analysis for Canada, another three for the US, and one for Poland. All but one of the studies declared explicitly the presence or absence of conflicts of interest . All studies are cost-utility analyses. Most short-term models (1 year) applied a decision tree, while long-term analyses (5, 10, 30 years or lifetime) are Markov models or a combination of an initial decision tree and a Markov component. For the long-term models, applied discount rates are in agreement with national recommendations in all but one of the analyses. In this study, the manufacturer assumed an annual discount rate of 3% for both health and cost outcomes, although the CADTH guidelines recommend a 5% discount rate [19]. However, a 5% discount was applied in a sensitivity analysis. For further details we refer to part 2.3.1 and Table 3 of the supplementary report.*  Table 1 General information on the identified economic evaluations   | **Study** | **Country** | **CoI** | **Analytic technique** | **Design** | **Time horizon** | **Discount rate***** | | --- | --- | --- | --- | --- | --- | --- | | **Archer et al., 2016** [17] | UK | No | CUA | Markov | lifetime | 3.5% | | AbbVie submission |  | Yes | CUA | Markov | 10y | 3.5% | | MSD submission |  | Yes | CUA | Decision tree + Markov | 10y | 3.5% | | **Assasi et al., 2009** [18] | Canada | No | CUA | Markov | 5y | 5% | | **Bodger et al., 2009** [23] | UK | No | CUA | Markov | lifetime (60y) | 3.5% | | **CADTH, 2014** [19] | Canada | Yes/No* | CUA | Markov | 10y | 3% | | **Dretzke et al., 2011** [20] | UK | No | CUA | Markov | 1y | / | | Abbott submission |  | Yes | CUA | Markov | 1y (56w) | / | | **Essat et al., 2014** [21] | UK | Yes/ No** | CUA | Decision tree + Markov | 10y | 3.5% | | **Kaplan et al., 2007** [24] | US | Yes | CUA | Decision tree | 1y | / | | **Loftus et al., 2009** [25] | UK | Yes | CUA | Regression model | 1y | 3.5% | | **Rafia et al., 2014** [22] | UK | Yes/ No** | CUA | Decision tree + Markov | 10y | 3.5% | | **Stawowczyk et al., 2016** [26] | Poland | No | CUA | Markov | 30y | C: 5%;  E: 3.5% | | **Tang et al., 2012** [27] | US | Not declared | CUA | Decision tree | 1y (54w) | / | | **Yu et al., 2009** [28] | US | Yes | CUA | Decision tree | 1y (56w) | / |   *C: costs; CoI: conflict of interest; CUA: cost-utility analysis; E: effects. w: weeks; y: year(s). * Submission by manufacturer reviewed by CADTH team (Common Drug Review Analyses) ** The manufacturer submitted a model-based health economic analysis as part of their submission, which was then evaluated by a team of researchers from ScHARR (School of Health and Related Research (ScHARR)).  *** Discount rate for both costs and effects, unless otherwise mentioned.*  ***Population and compared interventions***  *None of the studies included a paediatric population. The primary economic evaluations investigated treatment strategies for adult patients (average age 35-40 years and average weight 69-77kg) with moderate to severe UC or CD. In two studies, a secondary analysis is considered for the paediatric population [17, 20]. The authors consider this as an exploratory analysis as the efficacy data are drawn from trials undertaken within an adult UC population [17].*  *Most studies explicitly mention patients failed (intolerance, inadequate response or loss of response) to respond to standard therapy before ADA is considered. In all but three studies [24, 27, 28] and the MSD submission [17], conventional non-biological therapy is considered as a comparator. This usually exists of a mix of 5-aminosalicylates (5-ASAs), corticosteroids and immunosuppressants. In two studies, only biologicals are included [24, 28]. The study of Kaplan et al.[24] considered whether dose escalation of infliximab (IFX) (to 10 mg/kg every 8 weeks) is a cost-effective strategy compared with ADA initiation after loss of response to 5mg/kg of IFX. Also the study of Yu et al.[28] compares IFX and ADA. This study was also part of the Abbott submission, which contained two models: one comparing the cost-effectiveness of ADA as a maintenance therapy against standard care (SC) and one comparing the cost-effectiveness of ADA and IFX as maintenance therapies [20]. The report of Dretzke et al.[20] which made a critical assessment of Abbott’s submission concentrates on the model including standard care as a treatment option (see discussion).*  *In two studies [17, 21] and the MSD submission [17], surgery (colectomy) is taken into account as an initial treatment option. In these studies, surgery is included both as one of the alternative treatment strategies as well as a downstream component of the pathway for patients in the other treatment strategies. In other models, like the models discussed in the CADTH report [19] and from the AbbVie submission [17], surgery is not considered a direct comparator but only included as a treatment for patients who failed both biological and non-biological drug treatments.*  *Next to ADA, the most frequently included biological treatments are IFX, golimumab and vedolizumab. Certolizumab pegol and natalizumab are also included in individual studies. In most studies, ADA is administered as follows: induction: 160mg (week 0), 80mg (week 2); maintenance: 40mg every other week (starting from week 4) [17-19, 21, 24, 26-28]. In other studies the following treatment schedule is applied: induction: 80mg (week 0), 40mg (week 2); maintenance: 40mg every other week [20, 22, 23, 25]. The duration of treatment might also be different but is not always clearly stated. Bodger et al.[23] include 1 or 2 years of treatment with ADA or IFX after which patients return to standard care. For further information on the treatment schedule of the other interventions and dose escalation, we refer to part 2.3.2 and Table 4 of the supplementary report.*” |  |
| Risk of bias within studies | 19 | Present data on risk of bias of each study and, if available, any outcome level assessment (see item 12). | NA |
|  |  | This item is not relevant for this systematic review of economic evaluations. |  |
| Results of individual studies | 20 | For all outcomes considered (benefits or harms), present, for each study: (a) simple summary data for each intervention group (b) effect estimates and confidence intervals, ideally with a forest plot. | Page 10-17 |
|  |  | Summary information is provided for the following elements: ‘Costs’ (‘Costs of biological treatments’, ‘Costs of standard care’, ‘Costs of colectomy/surgery’), ‘Incremental costs related to adverse events’, ‘Quality of life’, ‘Treatment effect’, ‘Incremental cost-effectiveness ratios’, and ‘Uncertainty’. Table 1 (part 1-3) provides an overview of the ‘Results presented in the identified economic evaluations’. |  |
| Synthesis of results | 21 | Present results of each meta-analysis done, including confidence intervals and measures of consistency. | NA |
|  |  | This item is not relevant for this systematic review of economic evaluations. |  |
| Risk of bias across studies | 22 | Present results of any assessment of risk of bias across studies (see Item 15). | NA |
|  |  | This item is not relevant for this systematic review of economic evaluations. |  |
| Additional analysis | 23 | Give results of additional analyses, if done (e.g., sensitivity or subgroup analyses, meta-regression [see Item 16]). | NA |
|  |  | No specific additional analyses were performed. |  |
| **DISCUSSION** | | |  |
| Summary of evidence | 24 | Summarize the main findings including the strength of evidence for each main outcome; consider their relevance to key groups (e.g., healthcare providers, users, and policy makers). | Page 18-23 |
|  |  | Given the purpose of our research (i.e. perform a systematic review of the economic literature to provide input to the research protocol from a health economic point of view), we focussed our discussion on important issues that are linked from an economic point of view to the final research protocol of the REDUCE-RISK trial.  We mention the following at the beginning of our discussion: “*The overview of the economic literature allows us to identify important issues related to (the calculation of) the cost-effectiveness of ADA. A major strength is that this exercise was performed before the trial was started. In this way we avoid that important information was not measured in the trial. In this part, some of the identified issues are discussed and provide us support to provide input for the protocol from a health economic point of view.*”  The following elements are then discussed: ‘Paediatric population’, ‘Severity of disease’, ‘Adalimumab versus other biological treatment options’, ‘Treatment effect’, ‘Quality of life’, ‘Indirect costs’, and finally ‘Added elements in the research protocol’.  The research question was the following: “which additional elements should we include in the research protocol of the REDUCE-RISK trial to provide support to a high-quality economic evaluation?” In the last part of the discussion (“Added elements in the research protocol”), three separate elements that were added to the research protocol are described: ‘EQ-5D’ (page 21), ‘School attendance’ (page 22) and ‘Parents’ productivity’ (page 23). |  |
| Limitations | 25 | Discuss limitations at study and outcome level (e.g., risk of bias), and at review-level (e.g., incomplete retrieval of identified research, reporting bias). |  |
|  |  | Limitations at study and outcome level and at review level are not relevant in the context of this systematic review with a purpose to provide input from a health economic point of view to the research protocol of a trial.  One of the limitations is that we did not identify a suited questionnaire to measure the impact on school attendance. Therefore, we had to set up a new questionnaire, which is unfortunately a non-validated instrument. The following is mentioned in the discussion: “*No well suited questionnaire is thus identified that can be used for the international REDUCE-RISK trial. Therefore, a de novo school attendance questionnaire is set up. A limitation is that this is a non-validated questionnaire and that we cannot rely on the experience of other researchers with this questionnaire. Nevertheless, the choice is made to use this new instrument since we preferred to take the initiative to try to measure the impact with a non-validated instrument instead of not trying to measure this important aspect. The school attendance questionnaire exists of a version that is used at the start of the research and a version to be used at the follow-up visits.*” |  |
| Conclusions | 26 | Provide a general interpretation of the results in the context of other evidence, and implications for future research. | Page 23 |
|  |  | “*This paper addresses an important and progressive issue: including health economic considerations into the design of clinical trials. At the end of the trial, when all information on the intervention’s efficacy and safety has been gathered, the important incremental variables will be combined in a trial-based economic evaluation calculating the intervention’s incremental costs, effects and ICERs, both from a health care payer and societal perspective. Guidelines for performing economic evaluations will be followed. For example, parameter uncertainty will be included by performing probabilistic sensitivity analysis. Following the ISPOR guidelines, reporting of the methods and results of the economic evaluation will be performed according to the CHEERS (Consolidated Health Economic Evaluation Reporting Standards) guidelines [11, 12].*  *In conclusion, we are of the opinion that performing a systematic literature review supports researchers in setting up research protocol. In our case, the results of the literature review helped us to identify important variables for which evidence should be gathered in the REDUCE-RISK trial to allow the performance of a high-quality economic evaluation.*” |  |
| **FUNDING** | | |  |
| Funding | 27 | Describe sources of funding for the systematic review and other support (e.g., supply of data); role of funders for the systematic review. | Page 4 |
|  |  | In the introduction, we mention the following: “*The REDUCE-RISK trial, an international multicentre open-label prospective randomised controlled trial, has received funding from the European Union’s Horizon 2020 research and innovation programme under grant agreement No 668023. This trial has been reviewed and approved by national Ethics Services of participating countries and is prospectively registered (ClinicalTrials.gov Identifier: NCT02852694, date of registration: 09/06/2016, EudraCT Number: 2016-000522-18).*” |  |

*From:*  Moher D, Liberati A, Tetzlaff J, Altman DG, The PRISMA Group (2009). Preferred Reporting Items for Systematic Reviews and Meta-Analyses: The PRISMA Statement. PLoS Med 6(6): e1000097. doi:10.1371/journal.pmed1000097

For more information, visit: **www.prisma-statement.org**.

Page 2 of 2
